# Microenvironment-engineered electrified membrane for reactive separation of ammonia from wastewater

**DOI:** 10.1126/sciadv.aef5699

**Published:** 2026-07-15

**Authors:** Jianan Gao, Qingquan Ma, Shuaijie Zhao, Qing Li, Qingzhi Liu, Yanbiao Liu, Chuyang Y. Tang

**Affiliations:** ^1^ Membrane-based Environmental and Sustainable Technology (MembEST) Group, Department of Civil Engineering, The University of Hong Kong, Pokfulam, Hong Kong SAR 999077, China.; ^2^ Department of Civil, Architectural and Environmental Engineering, North Carolina Agricultural and Technical State University, Greensboro, NC 27411, USA.; ^3^ School of Environmental Science and Technology, Key Laboratory of Industrial Ecology and Environmental Engineering (Ministry of Education), Dalian University of Technology, Dalian 116024, China.; ^4^ College of Chemistry and Chemical Engineering, Dezhou University, Dezhou 253023, China.; ^5^ Department of Civil & Environmental Engineering, National University of Singapore, Singapore 117576, Singapore.

## Abstract

Source-separated recovery of nitrogen pollutants can enable nitrogen circularity and reduce the burden on wastewater infrastructure, yet most electrochemical systems struggle to couple high flux with low energy consumption. Here, we report a reagent-free electrified membrane that generates interfacial alkalinity via water dissociation at the membrane-wastewater boundary, drives ammonium (NH_4_^+^) to ammonia (NH_3_) conversion, and continuously extracts NH_3_ across the membrane as a high-purity stream. An integrated microenvironment-engineered design increases interfacial alkalinity and reduces gas-transport resistance, thereby optimizing NH_4_^+^/NH_3_ speciation and transmembrane flux with low energy consumption. The electrified membrane achieves the highest reported NH_3_ separation rate among electrochemical recovery systems and averages 2.9 times that of conventional structure while retaining 87.5% of its initial performance after 500 hours of flow-type operation. Preliminary building-scale life-cycle assessment and techno-economic analysis indicate notable environmental benefits and economic potential, with 2.34 square meters of membrane achieving a 95% NH_3_ recovery target for a 440-resident apartment. This work sets a framework for electrochemical NH_3_ separation and outlines a pathway to practical, decentralized deployment.

## Teaser

A reagent-free electrified membrane enables decentralized recovery of high-purity ammonia from source-separated urine.

## INTRODUCTION

Global NH_3_ production is ∼190 million tonnes year^−1^ in 2024, with most produced by the Haber-Bosch process, which underpins modern agriculture but entails substantial energy use and carbon emissions (*1*, *2*). About 30% of this nitrogen ultimately enters sanitation systems as ammoniacal nitrogen (*3*). In sewers, urea and proteins hydrolyze to NH_3_, which is protonated to NH_4_^+^ and conveyed to wastewater treatment plants, where dilution increases the energy and chemical requirements for removal (*4*). Conventional biological treatment converts NH_4_^+^ to dinitrogen and accounts for roughly 5% of global nitrous oxide and 17% of total methane emissions (*5*–*7*). Growing populations are expected to increase demand for NH_3_ synthesis and ammoniacal nitrogen treatment by 2.3 and 3.6% per year, respectively (*8*, *9*). Expansion of centralized plants is costly and space limited, and it misses the efficiencies available from collecting and recovering resource-rich streams (*4*). Source separation at the point of generation, for example, urine, could reduce the burden on wastewater treatment plants and support low-carbon NH_3_ supply, with the potential to meet a meaningful share (14%) of fertilizer demand if fully recovered (*10*–*16*). Modular and smart systems make decentralized operation increasingly plausible, although field performance shows that purpose-built small-scale technologies are needed rather than miniaturized versions of conventional plants (*17*–*20*).

Among recovery-oriented options, ion exchange adsorbents are poorly suited to decentralized deployment because frequent regeneration creates spent brines that must be collected and transported off-site (*21*, *22*). Physical membrane routes [reverse osmosis (*23*), forward osmosis (*24*), membrane distillation (*25*), and gas-permeable contactors (*26*)] concentrate wastewater or transfer NH_3_ after feed alkalization, which leaves NH_4_^+^ for downstream capture or requires continuous base/acid handling that complicates deployment at small sites. Electrochemical processes, potentially powered by renewable electricity, provide a reagent-free route to compact NH_3_ separation (*27*, *28*). In electrochemical stripping, NH_4_^+^ first transports across ion exchange membranes by electromigration and is then deprotonated in alkaline bulk solution generated by cathodic hydrogen evolution reaction (*29*). NH_3_ is subsequently removed by air stripping (*30*, *31*) or gas-permeable membranes (*32*–*35*). The average energy intensity is 13.9 kilowatt-hours (kWh) kg^−1^-N (table S1), comparable to that of industrial biological treatment at 11.7–12.5 kWh kg^−1^-N when credit is given for avoided chemical use and recovered NH_3_. However, alkalinity and generated NH_3_ disperse in the bulk solution, leading to a low NH_3_ fraction and uniform NH_3_ concentration under a specific current density. The current-normalized NH_3_ flux is merely 10.3 (μM cm^−2^ hour^−1^)/(mA cm^−2^), which limits area-specific throughput. Complex stack design and costly ion exchange membranes further add capital and operational burden.

**Fig. 1.**
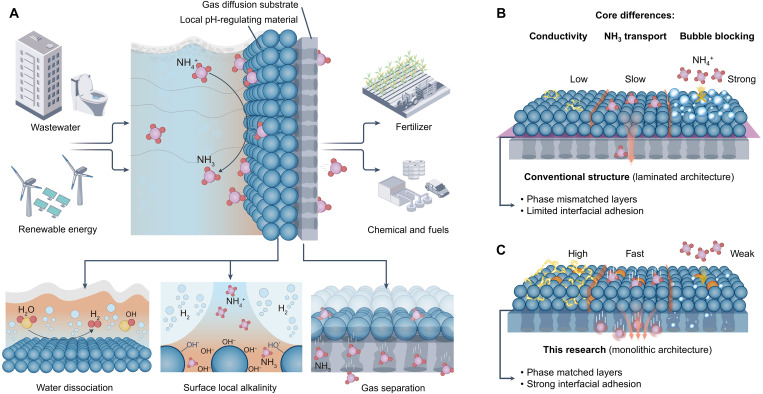
Schematic illustration of the continuous NH_3_ separation using an electrified membrane. (**A**) Illustration of a renewable energy–powered electrified membrane for low-carbon ammonia recovery from wastewater. The NH_4_^+^ ions near the membrane surface are first deprotonated to NH_3_ and then transported across the electrified membrane. The obtained pure NH_3_ could be further converted or used as a fertilizer, chemical, or fuel. The electrified membrane consists of a local pH-regulating material in contact with wastewater and a gas diffusion layer. The local pH-regulating material is responsible for electrical water dissociation and creates surface local alkalinity. The gas diffusion layer is responsible for NH_3_ separation. (**B** and **C**) Illustration of the core differences between a conventional electrified membrane (B) and the structure presented in this study (C).

Electrified membranes, comprising a local pH-regulating layer and a gas diffusion substrate, offer a more attractive alternative (Fig. 1A) (*36*–*40*). The alkalinity is confined to the membrane interface, where it converts electroadsorption accumulated NH_4_^+^ to NH_3_ and extracts it along a selective transport pathway. This approach delivers an average separation rate of 27.4 (μM cm^−2^ hour^−1^)/(mA cm^−2^) (table S1). Electrified membranes must resist wastewater flooding, maintain a stable three-phase boundary, and minimize resistance for both charge and gas transport. Conventional electrified membrane structures pair an intrinsically hydrophobic polytetrafluoroethylene (PTFE)– or polyvinylidene difluoride–based gas diffusion substrate with a separate conductive overlayer of carbon particles and/or carbon nanotubes (CNTs) (Fig. 1B) (*36*–*40*). Mismatches in surface chemistry, stiffness, and pore architecture at this interface promote delamination and contact resistance, adding barriers to NH_3_ transfer. To minimize the NH_3_ transport distances, we typically restricted the thickness of the conductive layer to a few micrometers, which increases in-plane resistance and causes nonuniform current distribution. Hydrogen evolution can cover active sites with bubbles, impede NH_4_^+^ delivery, and compete for gas pathways, further depressing flux and stability (*41*). These disadvantages elevate the energy intensity to 24.2 kWh kg^−1^-N. Therefore, an effective design should adopt a fused and monolithic architecture that eliminates weak interfaces, offers high in-plane conductivity, maintains low bubble coverage at the catalytic surface, and provides low-resistance gas channels.

In this study, we report an electrified membrane that delivers high NH_3_ separation rate and reduces energy consumption (Fig. 1C). The local pH-regulating material uses nickel phthalocyanine (NiPc) dispersed on multiwalled CNTs to accelerate water dissociation for rapid generation of local alkalinity at lower cathodic potential. The gas-side support is a PTFE-strengthened conductive carbon fiber network with intrinsically high in-plane conductivity, wetting resistance, and gas permeability, maintaining a stable three-phase region and a membrane surface with low bubble coverage under working conditions. A thin gas adsorption layer inserted between the pH-regulating layer and the gas diffusion substrate shifts NH_3_ transport from passive vapor partitioning to adsorption-mediated transfer, thereby increasing flux at a given current density. In the multilayer architecture, robust integration of composition-matched layers eliminates weak interfaces and preserves functional coordination across the layers. Building-scale life-cycle assessment (LCA) and techno-economic analysis (TEA) indicate strong potential for decentralized deployment of electrified membrane reactors, with benefits from avoided reagent logistics, reduced downstream treatment costs, and low-carbon NH_3_ production.

## RESULTS

### Fabrication and characterization of local pH-regulating material

**Fig. 2.**
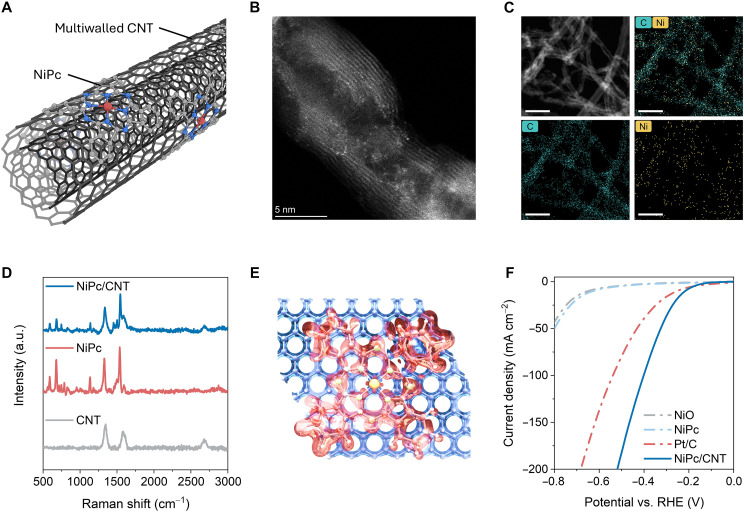
Structure characterization and water dissociation performance of NiPc/CNT. (**A**) A schematic illustration of the NiPc/CNT with NiPc molecules anchored on the sidewalls of CNTs. (**B** and **C**) High-resolution high-angle annular dark-field scanning transmission electron microscopy (HAADF-STEM) images and corresponding energy-dispersive x-ray spectroscopy (EDS) elemental mapping of NiPc/CNT. The high-contrast (white) dots in (B) are likely Ni atoms. In (C), the blue and yellow dots are C and Ni elements, respectively. Scale bars, 50 nm [all in (C)]. (**D**) Raman spectra of NiPc/CNT, NiPc, and CNT. a.u., arbitrary units. (**E**) Differential charge diagrams of NiPc/CNT. NiPc and CNT are placed at the top and bottom, respectively. Red and blue represent the accumulation and depletion of electrons, respectively. (**F**) Polarization curves of NiO, NiPc, Pt/C, and NiPc/CNT in simulated ammonium containing wastewater after activation.

For practical implementation, the pH-regulating material should be inexpensive and abundant. Beyond intrinsic activity, catalyst dispersion and interactions with the support strongly influence apparent performance (*42*). Molecular catalysts provide atomically defined, ligand-tunable sites with low overpotentials (*43*). CNTs offer high surface area and conductivity that promote uniform dispersion and rapid charge transport (*44*). Guided by these criteria, we selected NiPc supported on CNTs for screening (Fig. 2A) and benchmarked it against reported nickel oxide (NiO) and platinum (Pt) catalysts for interfacial alkalinity generation (*36*, *45*). Density functional theory indicates favorable free energies for water adsorption and dissociation and for H adsorption on NiPc, consistent with efficient hydrogen evolution activity (figs. S1 to S4).

NiPc was immobilized on the surface of multiwalled CNTs via π-π interactions, yielding NiPc/CNT (*46*). Inductively coupled plasma mass spectrometry (ICP-MS) indicated ∼2.8 wt % NiPc loading, equivalent to ∼14 μg cm^−2^ on the working electrode. Cyclic voltammetry evidenced effective anchoring (fig. S5), and integration of the reductive feature gave an electrochemically accessible NiPc loading of ∼6.1 μg cm^−2^, corresponding to ∼44% of the total mass obtained by ICP-MS (*47*). The high electrochemically active fraction indicates the molecular-level dispersion of NiPc on the CNT surface (*48*). X-ray diffraction (XRD) and x-ray photoelectron spectroscopy (XPS) confirmed that NiPc retained its original chemical states on CNTs (figs. S6 and S7). Aberration-corrected high-angle annular dark-field scanning transmission electron microscopy (HAADF-STEM) with energy-dispersive x-ray spectroscopy (EDS) mapped Ni at the single-molecule level on individual CNTs (Fig. 2, B and C, and figs. S8 to S10). Raman spectra showed attenuated bands below 1000 cm^−1^ upon supporting, consistent with electronic coupling between NiPc and CNTs (Fig. 2D) (*49*). Crystal orbital Hamilton population analysis and partial density of states revealed pronounced C─Ni(3d) bonding around 0.71 eV below the Fermi level, indicating strong electronic coupling between the CNTs and NiPc (figs. S11 and S12). The charge density difference maps indicated electron transfer from CNTs to NiPc (Fig. 2E and fig. S13). Electrochemical tests in simulated ammonium wastewater (0.25 M NH_4_^+^ in 0.1 M Na_2_SO_4_, pH 9.0; table S2) (*33*) showed high activity of NiPc/CNT and highlighted the importance of molecular dispersion, as reflected in the polarization behavior (Fig. 2F and fig. S14). On this basis, NiPc/CNT was selected as the local pH-regulating material for all electrified membranes investigated in this study.

### Quantification of surface local alkalinity

**Fig. 3.**
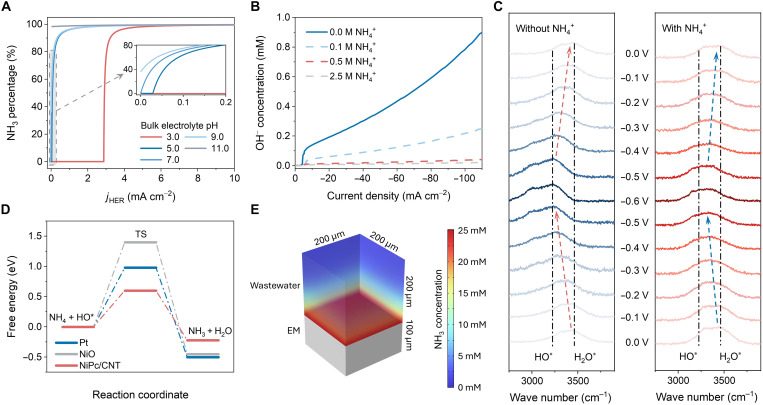
Investigation of local alkalinity at catalyst surface and its effect on NH_4_^+^-to-NH_3_ conversion. (**A**) Theoretical calculation of interfacial pH in relation to hydrogen evolution reaction current density (*j*_HER_). (**B**) Measured OH^−^ concentration with or without the presence of NH_4_^+^ ions. (**C**) In situ surface-enhanced IR spectra of NiPc/CNT at various potentials in different simulated wastewaters [pH 9.0, 0.1 M Na_2_SO_4_ with or without 1.25 M (NH_4_)_2_SO_4_]. (**D**) Free energy calculation of the transformation of NH_4_^+^ and HO^*^ to NH_3_ and H_2_O on the surface of Pt, NiO, and NiPc/CNT. (**E**) FEM modeling of the NH_3_ concentration gradients near the electrified membrane surface (current density: 10 mA cm^−2^, reaction time: 300 ms). TS, transition state; EM, electrified membrane.

Interfacial alkalinity consists of free hydroxyl anions (OH^−^) in the diffusion layer and surface-bound hydroxyls (M-OH*) on the catalyst (*50*, *51*). We first quantified the kinetic role of interfacial alkalinity by relating the cathodic pH gradient to current density using transport balances for H^+^ and OH^−^ within the Nernst layer (fig. S15). Because ammoniacal wastewaters are weakly buffered, with only low carbonate levels, buffering was neglected. Assuming that both free OH^−^ and surface-bound OH* contribute to NH_4_^+^ deprotonation, we modeled the NH_3_ fraction as a function of interfacial pH. The analysis was used to estimate the minimum current density required for NH_4_^+^-to-NH_3_ conversion under quiescent conditions. Across common wastewater pH conditions (5.0 to 9.0), an NH_3_ fraction of ∼99% is achieved at about 3.9 mA cm^−2^ (Fig. 3A). We then probed interfacial alkalinity experimentally using an iridium oxide (IrO*_x_*)–modified rotating ring-disk electrode (RRDE), which quantitatively reflects local OH^−^ concentration via the ring open-circuit potential (figs. S16 and S17) (*52*). The pH-regulating layer tested here, comprising NiPc/CNT, carbon particles, and perfluorosulfonic acid (PFSA), is identical in composition to that used in the electrified membranes evaluated in this study. Increasing the NH_4_^+^ concentration from 0.1 to 2.5 M markedly reduced the measured local OH^−^ signal (Fig. 3B), underscoring the central role of OH^−^ in NH_4_^+^ deprotonation. In situ infrared (IR) reflection spectroscopy showed a band at 3460 cm^−2^ assigned to adsorbed water (H_2_O*) (*53*), which shifted toward OH* features as the potential was stepped from 0.0 to −0.6 V versus reversible hydrogen electrode (RHE; Fig. 3C and fig. S18). Upon adding NH_4_^+^, these OH*-related signatures were attenuated, consistent with the consumption of OH* and its desorption to OH^−^ at high coverage (*54*, *55*). Energetic considerations suggest a possible complementary pathway in which NH_4_^+^ reacts with OH* to yield NH_3_ and H_2_O (Fig. 3D). Together, these results indicate that both OH^−^ and OH* participate in the NH_4_^+^-to-NH_3_ conversion, with OH^−^ making the dominant contribution. Finite element method (FEM) modeling under working conditions further mapped the transient microenvironment (fig. S19). At 10 mA cm^−2^, the interfacial NH_3_ concentration exceeded 25 mM within 300 ms, creating the partial-pressure gradient required for transmembrane transfer (Fig. 3E and fig. S20). These measurements and simulations reveal a quantitative link between applied current, interfacial alkalinity, and NH_3_ fraction, thereby defining operating windows for the electrified membranes.

### Architecture and interface of electrified membrane

**Fig. 4.**
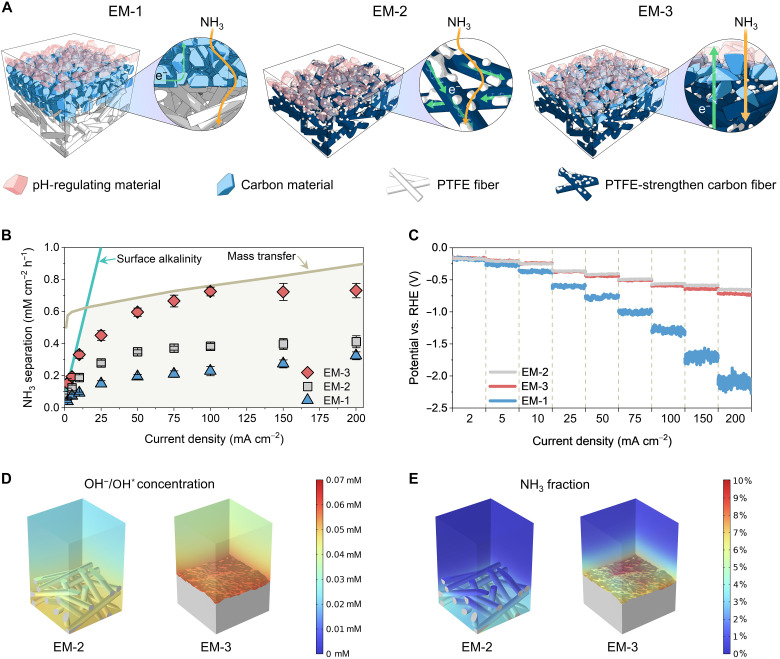
Interfacial architecture of electrified membranes for NH_3_ separation. (**A**) Schematic diagrams of the architectures of EM-1, EM-2, and EM-3. (**B**) Comparison of theoretical maximum NH_3_ separation rate and measured NH_3_ separation rate of EM-1, EM-2, and EM-3. The blue and brown lines represent theoretical values based on surface alkalinity and mass transfer limitation (free diffusion and electromigration) at different current densities, respectively. The experimental data are presented as the mean ± SD from at least three independent experiments. h, hours. (**C**) The recorded cathodic potentials of EM-1, EM-2, and EM-3 during the operation of NH_3_ separation. The total NH_4_^+^/NH_3_ concentration was 0.25 M with 0.1 M Na_2_SO_4_ aqueous electrolyte (pH 9.0) to simulate the ionic strength of environmentally relevant wastewater conditions. (**D** and **E**) FEM modeling of the OH^−^/OH* concentration gradients (D) and the relevant NH_3_ fraction percentage (E) near the electrified membrane surface of a two-dimensional rough surface and a three-dimensional fiber network (current density: 10 mA cm^−2^, reaction time: 300 ms).

Beyond interfacial alkalinity, an effective electrified membrane must balance hydrophobicity, electronic conductivity, and gas permeability. To benchmark the architecture, we fabricated a conventional structure consisting of a PTFE-based gas diffusion substrate coated with a ∼5.5-μm-thick conductive overlayer composed of carbon particles and CNTs (named EM-1; Fig. 4A). Cross-sectional HAADF-STEM revealed a sharp interface between the layers (fig. S21), consistent with interfacial stratification that favors hydrophobicity but penalizes conductivity and gas transport. We then constructed a monolithic, intrinsically conductive carbon fiber network reinforced with a PTFE hydrophobic treatment (named EM-2). Last, to transform the membrane-water contact from a three-dimensional network to an approximately two-dimensional interface, we introduced a thin hydrophobized carbon microporous layer (named EM-3). The blurred layer boundaries in EM-3 indicate improved structural integrity (fig. S22). All three variants exhibited similar surface roughness and high water contact angles (148.2° to 170.7°), confirming comparable wetting resistance (figs. S23 to S26 and table S3).

To isolate transport effects from electrochemical alkalization, we first imposed interfacial pH by adjusting the bulk electrolyte pH rather than relying on water dissociation. Under these conditions, the NH_3_ separation rate followed EM-3 > EM-1 > EM-2 (fig. S27), indicating that a quasi–two-dimensional, rough interface promotes faster NH_3_ transfer in the absence of hydrogen (H_2_) bubble interference. For subsequent tests, all membranes used the NiPc/CNT pH-regulating layer (0.5 mg cm^−2^) deposited by nitrogen-gas spray with Vulcan XC-72 carbon particles and PFSA and were evaluated in a scalable flow-by configuration (figs. S28 to S33).

For the electrochemically driven NH_3_ separation, we used a predictive model that combines interfacial alkalinity with diffusion and electromigration limitations to estimate the upper bound of the NH_3_ flux (blue and brown lines in Fig. 4B and fig. S34). The model assumes that all locally generated alkalinity drives NH_4_^+^ to NH_3_ conversion and that the formed NH_3_ is immediately extracted. Deviations between the predicted and experimental values suggest the presence of gas-phase transport limitations under operating conditions. EM-3 exhibited the highest measured NH_3_ separation rate (figs. S35 and S36). The measured NH_3_ separation rates of EM-1, EM-2, and EM-3 corresponded to ∼28, 48, and 85% of the theoretical limit, respectively (fig. S37 and table S4).

Hydrogen evolution further differentiates architectures. Bubble nucleation covers active sites, impedes NH_4_^+^ delivery, competes for gas pathways, and increases overpotential (*41*). EM-2 and EM-3 exhibited lower surface bubble coverage than EM-1 (fig. S38 and movies S1 and S2), and separated H_2_ was observed on the gas diffusion side (fig. S39). These effects, together with the higher in-plane resistance of EM-1, explain the higher cathodic potential required for this membrane. The average operating potential of EM-1 was 2.1 times that of EM-3 (Fig. 4C and table S5). Although EM-2 benefits from a conductive and integrated backbone that expedites bubble removal, its pH-regulating sites are embedded within a three-dimensional fiber network. The resulting alkalinity diffuses through a larger volume, yielding a lower interfacial NH_3_ fraction than that in EM-3 (Fig. 4, D and E, and figs. S40 and S41). Together, these results identify EM-3, an approximately two-dimensional distributed pH-regulating layer integrated with an intrinsically conductive gas diffusion network, as the most effective structure for NH_3_ separation. This finding provides quantitative targets for further improvement in lateral conductivity and gas phase transport of the electrified membranes.

### Local microenvironment regulation

**Fig. 5.**
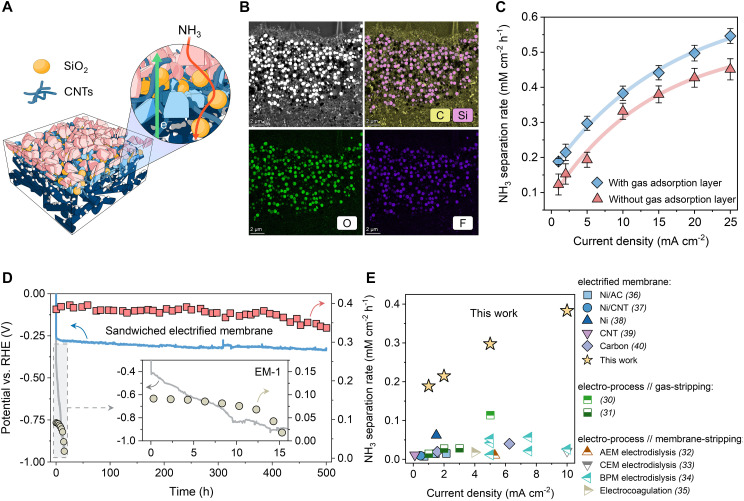
Local microenvironment regulation to enhance NH_3_ separation. (**A**) Schematic diagram of local microenvironment regulation by introducing a gas adsorption layer into the electrified membrane. (**B**) HAADF-STEM and EDS elemental mapping image of the gas adsorption layer of the sandwiched electrified membrane. (**C**) The NH_3_ separation rates with or without gas adsorption layer. The experimental data are presented as the mean ± SD from at least three independent experiments. (**D**) Long-term stability test of the sandwiched electrified membrane and EM-1 for comparison. (**E**) Comparison of NH_3_ separation rate with reported state-of-the-art electrochemical processes (*31**–**40*).

In electrified membranes, NH_3_ transfer is driven by the interfacial partial-pressure gradient. Thus, strengthening the local microenvironment to raise the interfacial NH_3_ concentration could increase flux. We developed a sandwiched architecture in which a thin gas adsorption layer is placed between the pH-regulating layer and the carbon fiber network (Fig. 5A and fig. S42). The gas adsorption layer comprises siloxane-modified silicon dioxide (SiO_2_) microspheres dispersed within a conductive network of carbon particles and CNTs (Fig. 5B and figs. S43 to S45). SiO_2_ microspheres were selected for dispersibility and chemical stability. Random packed SiO_2_ microparticles generate hydrophobic interstitial capillaries that collect and remove NH_3_ and H_2_ gasses from the pH-regulating layer. Adding the gas adsorption layer increased the NH_3_ separation rate for EM-1, EM-2, and EM-3, while EM-3 still delivered the highest performance (fig. S46). On this basis, EM-3 was selected for subsequent investigation. Particle size (0.08 ± 0.01, 0.42 ± 0.05, and 0.90 ± 0.07 μm) and loading density (2 to 10 mg cm^−2^) of SiO_2_ microspheres were tuned to set channel geometry and transport length (figs. S47 to S55).

The gas adsorption layer enhanced NH_3_ transport, increasing the separation rate by up to 32% (fig. S56) and yielding an average ∼25% improvement across the operating range of 1 to 25 mA cm^−2^ (Fig. 5C). The carbon network in the adsorption layer contributed negligibly to NH_3_ separation (fig. S57). Molecular dynamics simulations corroborated enhanced transport, with the mean square displacement of NH_3_ through the gas pathway increasing from 26.3 to 34.3 Å^2^ ps^−1^ upon adding SiO_2_ (figs. S58 to S61). The nonconductive and hydrophobic SiO_2_ microspheres also improved antiflooding property and resistance to electrowetting, while electrochemical impedance spectroscopy (EIS) and cathodic potential monitoring confirmed that careful control of SiO_2_ loading preserved membrane conductivity (figs. S62 and S63).

During operation, repeated H_2_ bubble generation and NH_3_ transport through the membrane imposed interfacial peeling stresses. Because EM-1 consists of phase-mismatched layers with limited interfacial adhesion, partial detachment of conductive materials and pH-regulating materials from the gas exchange substrate was observed. The resulting structure disruption caused an 80.3% decline in the NH_3_ separation rate and a 113.2% increase in the operating potential after 15.9 hours (Fig. 5D). The sandwiched electrified membrane showed sustained operation, retaining 87.5% of its initial NH_3_ separation rate after 500 hours, with a 29.4% rise in cathodic potential and no detectable Ni leaching by ICP-MS. The improved durability arises from its integrated architecture, which eliminates poorly bonded laminated interfaces, preserves continuous electronic and gas transport pathways, and reduces bubble-induced mechanical damage during operation. Across 1 to 10 mA cm^−2^, the sandwiched electrified membrane achieved NH_3_ separation rate ranging from 0.19 to 0.38 mM cm^−2^ hour^−1^, outperforming reported electrified membranes (*36*–*40*), electro-process/air-stripping (*30*, *31*), and electro-process/membrane-stripping systems (*32*–*35*) (Fig. 5E). These results confirm the effectiveness of local microenvironment regulation via a gas adsorption layer to raise NH_3_ flux while maintaining long-term stability.

### Potential economic and decarbonization benefits of source separation

On the basis of the long-term flow-type experiment at 10 mA cm^−2^, an NH_3_ separation rate of 559 kg m^−2^ year^−1^ was achieved, indicating strong potential for practical applications. We evaluated sustainability and cost via LCA and TEA (tables S6 to S14). Because the electrified membrane process is reagent free and electricity driven, CO_2_ emissions were estimated using the US average grid intensity (0.348 kg-CO_2_ kWh^−1^). Electrically driven source NH_3_ recovery also avoids downstream N_2_O formation (global warming potential 273 times that of CO_2_) (*56*), yielding the lowest CO_2_ emissions among traditional NH_3_ removal or synthesis options (fig. S64A).

TEA indicates attractive unit economics at high-strength points of generation, such as urine, which carries 80 to 90% of household nitrogen in around 1% of the volume (*57*). Considering the presence of chloride ions in urine, we implemented a paired anodic chlorine evolution and separation module to corecover NH_3_ and Cl_2_ (fig. S65). The electricity cost was $0.37 to $0.52 per kg-NH_3_, with Cl_2_ coproduced at 72 to 107% of the NH_3_ mass (fig. S66 and table S15). Building-scale analysis for 440 residents projects a required electrified membrane area of 2.34 m^2^ to achieve an NH_3_ recovery efficiency of 95% (fig. S67 and table S16 to S19). Under the conventional pathway, where urine is conveyed to a wastewater treatment plant, the annual cost for centralized treatment is $3384 (fig. S64B). For the source separation alternative, annual revenues from recovered chemicals ($979) are sufficient to offset electricity costs ($516) and downstream wastewater treatment plant costs for residual ammonium treatment ($169). Source NH_3_ separation based on the electrified membrane could potentially lower private costs and climate-related damage while producing useful chemical products for sale or on-site use. It can defer investments in sewers and centralized treatment while enabling local water reuse, supporting system-wide decarbonization of ammonia and wastewater services (*18*). However, the present LCA/TEA is intended as a preliminary assessment of operational and system-level potential. A full analysis will require future incorporation of capital cost, membrane scale-up and replacement, module fabrication, and installation at practical scales.

## DISCUSSION

We establish an integrated electrified membrane framework for source ammonia recovery that is systematic from end to end: a molecularly defined pH-regulating material, a conductive and hydrophobic electrode scaffold, and deliberate regulation of the reaction microenvironment. By coupling NiPc/CNT-driven interfacial alkalinity with a monolithic, PTFE-strengthened carbon fiber support and a SiO_2_ adsorption layer that accelerates gas transfer, we link mechanism to architecture and translate that linkage into performance. The result is a high NH_3_ separation rate that surpasses all published electrochemical recovery–related studies while maintaining a simple, modular construction.

This concept reframes ammoniacal wastewater as a local resource. It enables decentralized operation compatible with renewable electricity, reduces downstream treatment burdens, and provides a route to on-site product use. Equally important, the work offers a coherent framework: (i) quantitative ties between interfacial pH, NH_4_^+^/NH_3_ speciation, and flux; (ii) design rules that balance hydrophobicity, conductivity, and permeability; and (iii) a microenvironment design that boosts transfer without bulk chemical dosing.

Future work should extend this framework toward scalable module architecture, stability test of various real wastewaters, and advanced manufacturing strategies for electrified membranes. More standardized LCA and TEA studies will further support practical implementation and policy evaluation.

## MATERIALS AND METHODS

### Materials and chemicals

*N*,*N*′-dimethylformamide (DMF; 99.5%), sodium hydroxide (98.0%), salicylic acid (99.0%), sodium citrate dihydrate (99.0%), sodium hypochlorite solution (0.1 M), sodium nitroferricyanide dihydrate (99.0%), ammonium chloride (99.8%), sodium sulfate (99.0%), ammonia solution (25 to 28%), tetraethyl orthosilicate (99%), and dodecyltrimethoxysilane (93%) were purchased from Aladdin Scientific. Multiwalled CNTs (inner diameter: 70 to 80 nm, outer diameter: 90 to 100 nm, and length <10 μm) and trimethoxy(octadecyl)silane (95%) were obtained from Macklin Biochemical. NiPc (98%) was obtained from Bidepharm. Nafion D521 solution was obtained from DuPont. Ethanol (99.8%) was obtained from VWR Chemicals. Deionized water was used throughout. Unless otherwise noted, all reagents were used as received.

### Preparation of NiPc/CNT

NiPc was supported on CNTs via noncovalent π-π interactions following a reported procedure (*46*). As-received multiwalled CNTs were first calcined in air at 500°C for 5 hours at 1°C min^−1^ and then sonicated in 5 wt % HCl for 30 min and stirred overnight. The solids were washed with water to neutral pH and freeze dried to yield purified CNTs. Separately, purified CNTs (30.0 mg in 30 ml of DMF) and NiPc (1.5 mg in 15 ml of DMF) were sonicated for 1 hour to form a CNT dispersion and a NiPc dispersion. The two dispersions were then mixed, sonicated for another 1 hour, and stirred at room temperature for 24 hours. The mixture was centrifuged, and the supernatant was discarded. The precipitate was washed sequentially with DMF, ethanol, and water (centrifuged twice at each step) until the supernatant was colorless and then freeze dried to give NiPc/CNT. ICP-MS indicated a NiPc loading of ∼2.8 wt %.

### Characterization

ICP-MS was performed on an Agilent 720-ES (optical emission spectrometry) instrument. XRD used a Rigaku Ultima IV diffractometer. XPS was carried out on a Thermo Fisher Scientific ESCALAB QXi (Al Kα, 1486.6 eV) with charge neutralization. Spectra were referenced to C 1s at 284.6 eV. High-resolution SEM used a LEO 1530 field-emission instrument (Schottky emitter). HAADF-STEM images were acquired on a Thermo Fisher Scientific Spectra 300 TEM or a Talos F200X microscope. Raman spectra were collected on a LabRAM HR Evolution (HORIBA Jobin Yvon) with a 532-nm laser. Static water contact angles were measured on a Biolin Scientific Attension Theta goniometer. Surface topography of electrified membranes was obtained by noncontact optical profilometry (Bruker ContourGT-K 3D).

### Electrochemical measurements

Hydrogen evolution activity of local pH-regulating materials was evaluated in a three-electrode cell using a Zahner Zennium potentiostat. A home-made electrode, Ag/AgCl in saturated KCl, and a graphite plate served as the working electrode, reference electrode, and counter electrode, respectively. Measurements were conducted in simulated ammonium-containing wastewater. Solution resistance (*R*_u_) was determined by EIS (9.5 ± 0.3 ohm), and 85% *iR* compensation was applied (*58*). All potentials are reported versus the RHE.

The measurement of surface pH was carried out by an RRDE system. The potential of IrO*_x_* is sensitive to proton activity and serves as a local pH indicator (*52*, *59*). Interfacial pH at the catalyst surface was tracked using the RRDE with an IrO*_x_*-functionalized ring. IrO*_x_* was electrodeposited on the ring by cyclic voltammetry and calibrated in simulated ammonium-containing wastewater by recording the ring open-circuit potential (*E*_oc_) at known pH. A linear relation was obtained

$${\text{pH}}_{\text{ring}}=a\times \left(E_{\text{oc}}+b\right)$$ (1)

where *a* and *b* were obtained from least-squares regression. To probe interfacial pH under bias, a disk coated with the same NiPc/CNT ink and loading as used in the electrified membranes was swept by linear sweep voltammetry, while the IrO*_x_* ring was left at open circuit and recorded continuously. Instantaneous *E*_oc_ values were converted to pH_ring_. The catalyst-surface pH at the disk was inferred from the ring measurement using

$$\begin{array}{c} C_{H^{+},\text{ring}}-C_{{\text{OH}}^{-},\text{ring}}=N_{D}\times \left(C_{H^{+},\text{disk}}-C_{{\text{OH}}^{-},\text{disk}}\right)\\ +\left(1-N_{D}\right)\times \left(C_{H^{+},\text{bulk}}-C_{{\text{OH}}^{-},\text{bulk}}\right) \end{array}$$ (2)

where $C_{H^{+},\text{ring}}$, $C_{{\text{OH}}^{-},\text{ring}}$ and $C_{H^{+},\text{disk}}$, $C_{{\text{OH}}^{-},\text{disk}}$ are the concentrations of H^+^ and OH^−^ at the ring and the disk, respectively; $C_{H^{+},\text{bulk}}$ and $C_{{\text{OH}}^{-},\text{bulk}}$ are the concentrations of H^+^ and OH^−^ in bulk wastewater, respectively; and *N*_D_ = 0.27 is the ring detection efficiency.

In situ surface-enhanced IR spectra were collected on a Nicolet iS50 FTIR spectrometer (Thermo Fisher Scientific). A NiPc/CNT ink (identical in composition and loading to that used on the electrified membranes) was deposited onto a gold-coated silicon prism (working electrode). Spectra were acquired in a two-compartment H cell separated by a Nafion 117 membrane, with a platinum foil counter electrode and a Ag/AgCl reference electrode. Measurements were carried out in simulated wastewater, with and without ammonium ions, at a spectral resolution of 0.48 cm^−1^.

### Finite-element multiphysics (FEM)

A coupled FEM model (electric currents, transport of diluted species with electromigration, and bubbly two-phase flow) was used to compute interfacial OH^−^/OH*, NH_4_^+^, and NH_3_ fraction at the membrane-wastewater boundary (*60*).

### Preparation of electrified membranes

Unless otherwise noted, all electrified membranes comprise a gas diffusion substrate onto which the pH-regulating ink was spray coated. Spraying was performed using a spray gun (Blue Brand LP-186) with a nitrogen gas flow at 25 psi. For EM-1, a hydrophobic laminate consisting of polyethylene terephthalate nonwoven fabric support and a PTFE fiber layer (0.45-μm nominal pore size; 160 μm thick) served as the gas diffusion substrate. A conductive layer was prepared by dispersing 20 mg of CNTs in 10 ml of anhydrous ethanol and 120 μl of Nafion, sonicating for 1 hour. Then, 60 mg of carbon particles were added to the solution and sonicated for another 1 hour. A total carbon material loading of 20 mg cm^−2^ was sprayed to the PTFE side. The substrate was dried 24 hours at room temperature.

For EM-2 and EM-3, carbon fiber paper (Toray H-060; 30% hydrophobic treatment, 190 μm thick) and carbon fiber paper with a hydrophobic carbon microporous layer (AvCarb GDS2230, 260 μm thick) were used for EM-2 and EM-3, respectively. To enhance hydrophobicity and mechanical robustness, the carbon fiber face of each substrate was spray coated with 10 wt % PTFE (diluted from a 60 wt % dispersion; Daikin D-210C) and calcined at 270°C for 10 min. This cycle was repeated three times to reach a PTFE coverage of 10 ± 0.1 mg cm^−2^.

For EM-1 to EM-3, 10 mg of NiPc/CNT and 10 mg of Vulcan XC-72 were dispersed in 10 ml of anhydrous ethanol and 30 μl of Nafion by sonicating for 1 hour to form the spray ink. The ink was applied to the conductive side of EM-1 and to the non–PTFE-treated face of EM-2 and EM-3 over an active area of 4 cm^2^ to achieve 0.5 mg-NiPc/CNT cm^−2^. The obtained electrified membranes were dried for 24 hours at room temperature before tests.

### Preparation of sandwiched electrified membranes

The sandwiched electrified membrane incorporates a gas adsorption layer between the gas diffusion substrate and the pH-regulating layer. The gas adsorption layer comprises siloxane-modified SiO_2_ microspheres, carbon particles, and CNTs. A dispersion of 5 mg of CNTs in 10 ml of ethanol and 30 μl of Nafion was sonicated for 1 hour. Then, 15 mg of carbon black and 150 mg of SiO_2_ were added and sonicated for another 1 hour. The ink was sprayed onto the microporous layer side of the PTFE-strengthened EM-3 substrate to achieve SiO_2_ loadings of 2, 5, or 10 mg cm^−2^. Substrates were dried for 24 hours and then coated with the pH-regulating layer as above.

### Flow-type NH_3_ separation experiments

Figure S33 shows the flow-type electrolyzer, comprising a wastewater channel and a trap channel. Both channels expose a 15 mm–by–15 mm active window. Silicone gaskets ensure leak-tight seals. Inlets and outlets (4-mm outer diameter, 2-mm inside diameter) were used for flow. The electrified membrane separated the channels, with the pH-regulating layer facing the wastewater and the PTFE-treated gas diffusion side facing the trap channel to enable NH_3_ extraction. A platinum plate immersed in the wastewater channel served as the anode. Simulated ammonium containing wastewater was pumped through the wastewater channel at 6 ml min^−1^ using a peristaltic pump. For simultaneous separation tests, gaseous products were collected from the trap channel and quantified at fixed intervals.

## Supplementary Materials

### The PDF file includes:

Supplementary Text Figs. S1 to S69 Tables S1 to S22 Legends for movies S1 and S2 References
