# Supplementary material for: Microenvironment-engineered electrified membrane for reactive separation of ammonia from wastewater
Source: Sci Adv. 2026 Jul 15;12(29):eaef5699. doi: 10.1126/sciadv.aef5699 (PMC13371912; doi:10.1126/sciadv.aef5699)
Supplement: Supplementary file 1 — Supplementary Text Figs. S1 to S69 Tables S1 to S22 Legends for movies S1 and S2 References [file sciadv.aef5699_sm.pdf]

Science Advances
AAAS

# Supplementary Materials for

## Microenvironment-engineered electrified membrane for reactive separation of ammonia from wastewater

Jianan Gao *et al.*

Corresponding author: Yanbiao Liu, yanbiaoliu@dlut.edu.cn; Chuyang Y. Tang, cy_tang@nus.edu.sg

*Sci. Adv.* **12**, eaef5699 (2026)
DOI: 10.1126/sciadv.aef5699

**The PDF file includes:**

Supplementary Text
Figs. S1 to S69
Tables S1 to S22
Legends for movies S1 and S2
References

**Other Supplementary Material for this manuscript includes the following:**

Movies S1 and S2

## Supplementary Text

### Text S1. Quantifying electrochemically active NiPc on CNTs surface

We quantified the electrochemically active NiPc on CNTs surface by cyclic voltammetry curves. According to the literature,(*50, 61*) the negative cathodic current is mainly the reduction current of transition metal ions and does not include the Faradaic current. Therefore, we assume that the number of electrons associated with Nickel ion reduction ($Ni^{2+} + e^{-} \rightarrow Ni^{+}$) is proportional to the number of surface-active Ni sites on NiPc/CNT.

To make sure that all the surface Ni sites have been oxidized in the anodic scanning and can be reduced in the cathodic scanning, we measured the cyclic voltammetry curves of the two samples at different potential intervals (from 0.8 to 1.5, 1.6, 1.7, 1.8, 1.9, 2.0, and 2.1 V vs. RHE). As shown in **Fig. S1a**, the negative reductive currents during 0.8~2.0 V vs. RHE and 0.8~2.1 V vs. RHE almost overlap. Therefore, the cyclic voltammetry curves at the potential interval of 0.8~2.0 V vs. RHE were used for quantifying Ni sites on CNTs surface.

### Text S2. Theoretical calculation of interfacial pH and $NH_3$ fraction

During hydrogen evolution, $OH^{-}$ is generated at the cathode and a pH gradient develops such that $pH^{*} \geq pH^{b}$. At steady state, $H^{+}$ and $OH^{-}$ transport are treated as Fickian fluxes across a hydrodynamic diffusion layer. The total current density is the charge weight sum of these fluxes (*62*):

$$\begin{aligned} j = &(1000 \times F \times \frac{D_{H^{+}}}{\delta_{H^{+}}} \times 10^{-pH^{b}}) \times (1 - 10^{pH^{b} - pH^{*}}) \\ &+ (1000 \times F \times \frac{D_{OH^{-}}}{\delta_{OH^{-}}} \times 10^{pH^{b} - pKw}) \times (10^{pH^{*} - pH^{b}} - 1) \end{aligned} \tag{S1}$$

where $j$ is the current density, $pH^{*}$ and $pH^{b}$ are the pH of the electrified membrane interface and bulk wastewater, F is the Faraday constant, $D_{H^{+}}$ and $D_{OH^{-}}$ are the diffusion coefficients of $H^{+}$ and $OH^{-}$, $\delta_{H^{+}}$ and $\delta_{OH^{-}}$ are the diffusion layer thicknesses of $H^{+}$ and $OH^{-}$ as calculated by Levich equation.*(62)* The water ion product is expressed as $pK_{w}$.

The interfacial $NH_3$ fraction is calculated as (*27-28*):

$$\frac{[NH_3]}{[NH_3 + NH_4^{+}]} = \frac{10^{pH}}{e^{21.3} + 10^{pH}} \tag{S2}$$

At steady state, the $NH_3$ vapor flux ($j_{NH_3}$) across the membrane is driven by the $NH_3$ vapor pressure difference between the $NH_3$-rich interfacial region and the back side of the gas diffusion substrate (*63*):

$$j_{NH_3} = L_p(P_{\mathrm{NH_3,\,high}} - P_{\mathrm{NH_3,\,low}}) \tag{S3}$$

$$P_{\mathrm{NH_3}} = C_{\mathrm{NH_3}} \times k_{\mathrm{H}} \tag{S4}$$

where $L_p$ is the $NH_3$ permeability, $P_{\mathrm{NH_3,\,high}}$ and $P_{\mathrm{NH_3,\,low}}$ are the local $NH_3$ vapor pressures in the interfacial region and on the permeate side, $C_{\mathrm{NH_3}}$ is the local $NH_3$ concentration, and $k_H$ is the Henry's constant for $NH_3$ at room temperature (~25 °C). The simulation parameters are listed in **Table S20**.

**Text S3. $NH_3$ separation model**

We modelled $NH_3$ separation with two bounding regimes. (i) Current-controlled limit: with non-limiting $NH_4^+$ supply and instantaneous $NH_3$ removal. $OH^-$ from hydrogen evolution reaction drives the formation of $NH_3$, so $NH_3$ separation scales directly with applied current (assume the hydrogen evolution reaction Faradaic efficiency is 100%). (ii) Transport-controlled limit: $NH_4^+$ delivery to the cathode is limited by mass transfer across the flow boundary layer, combining diffusion and electromigration (Nernst-Planck). The achievable $NH_3$ flux at any operating point is the minimum of the two rates and is used for reactor sizing and energy/revenue estimates (*39*):

$$J_{\mathrm{current}} = \frac{I}{e \cdot N_{\mathrm{A}} \cdot A} \cdot \left[ \frac{\mathrm{NH_3}}{\mathrm{NH_3{+}NH_4^+}} \right] \tag{S5}$$

$$J_{\mathrm{mass}} = -D \times \frac{C_{\mathrm{bulk}} - C_{\mathrm{surface}}}{\delta} - z \times u_{\mathrm{NH_4^+}} \times C_{\mathrm{lm}} \times E \tag{S6}$$

where $I$ is the cathodic current, $e$ is the elementary charge, $N_A$ is Avogadro's constant, $A$ is the geometric area of electrified membrane, $D$ is the diffusion coefficient of $NH_4^+$, $C_{bulk}$ is the $NH_4^+$ concentration in bulk wastewater, $C_{surface}$ is the $NH_4^+$ concentration on the electrified membrane surface, $\delta$ is the thickness of diffusion layer, $z$ is the charge of $NH_4^+$, $u_{\mathrm{NH_4^+}}$ is the ionic mobility of $NH_4^+$, $C_{lm}$ is the logarithmic mean concentration accounts for the nonlinear $NH_4^+$ profile between the bulk solution and cathode surface, E is average electric field strength across the diffusion layer. The parameters used for calculation are listed in **Table S22**. The details of the formula derivation are listed below:

Current-controlled limitation. During the electrochemical $NH_3$ stripping process, the $NH_4^+$ ion first diffused to cathode surface, then converted to $NH_3$ due to the in-situ generated $OH^-$ from hydrogen evolution reaction on catalyst layer and finally separated from bulk solution crossing the gas diffusion layer of electrified membrane module. The calculation is based on the

assumption that: (1) $NH_4^+$ in the bulk solution is sufficiently abundant, such that its transport to the cathode is not rate-limiting. (2) all $NH_3$ formed at the catalyst/electrolyte interface is immediately removed across the gas diffusion layer, such that no product accumulation occurs near the interface. (3) the cathodic current is fully devoted to the hydrogen evolution reaction, i.e., the Faradaic efficiency for hydrogen evolution is 100%.

At the cathode, hydrogen evolution reaction in aqueous solution leads to $e^-$:$OH^-$ ration of 1:1, which means that one mole of electrons produces one mole of $OH^-$. Therefore, according to Faraday's law, the molar flux of $OH^-$ generated per unit membrane area is equal to current values per unit membrane area (current density). The generated $OH^-$ deprotonates ammonium-to-ammonia at a mole ratio of 1:1. Under the ideal assumptions above, the maximum molar $NH_3$ separation flux is therefore equal to the $OH^-$ generation flux, which is equal to the current density. This gives the theoretical current-controlled $NH_3$ separation rate in molar form. These relations indicate that, under the current-controlled limit, the $NH_3$ separation rate is linearly proportional to the applied current density as listed below:

$$J_{\text{current}} = \frac{I}{e \cdot N_{\text{A}} \cdot A} \tag{S7}$$

where $J_{\text{current}}$ is the highest $NH_3$ separation rate based on cathodic current density (mmol $cm^{-2}$ $h^{-1}$), I is the cathodic current (A), e is the elementary charge, $N_A$ is Avogadro's constant, A is the exposed geometric area of the electrified membrane. All calculation parameters are listed in Supplementary Table 21.

<u>Transport-controlled limitation.</u> In a flow-type electrochemical reactor where ammonium ions ($NH_4^+$) are reduced at the cathode to produce gaseous ammonia, the transport of $NH_4^+$ from the bulk solution to the cathode surface is governed by a combination of convection, diffusion, and electromigration. This process is modeled using the Nernst-Planck equation with flow-based boundary layer considerations.

$$J_{mass} = J_{diff} + J_{elec} \tag{S8}$$

where $J_{total}$ is the total molar flux of $NH_4^+$ (mmol $cm^{-2}$ $h^{-1}$) from the bulk solution to the cathode surface, $J_{diff}$ is diffusive flux driven by the $NH_4^+$ concentration gradient, $J_{elec}$ is electromigration flux driven by the applied electric field across the diffusion boundary layer.

The diffusive flux is calculated by:

$$J_{\text{diff}} = -D\frac{dC}{dx} = -D\frac{C_{\text{bulk}} - C_{\text{surface}}}{\delta} \tag{S9}$$

where $D$ is the diffusion coefficient of $NH_4^+$, $C_{\text{bulk}}$ is the $NH_4^+$ concentration in bulk solution, $C_{\text{surface}}$ is the $NH_4^+$ concentration on the electrified membrane surface (we assume complete consumption of $NH_4^+$ at the cathode, so a nonzero value was used for numerical stability), $\delta$ is the thickness of the diffusion layer.

The electromigration flux is calculated by:

$$J_{\text{elec}} = -z \cdot u_{\text{NH}_4^+} \cdot C_{\text{lm}} \cdot E \tag{S10}$$

where $z$ is the charge of $NH_4^+$, $u_{NH_4^+}$ is the ionic mobility of $NH_4^+$, $C_{\text{lm}}$ is the logarithmic mean concentration accounts for the nonlinear $NH_4^+$ profile between the bulk solution and cathode surface, $E$ is average electric field strength across the diffusion layer.

The Reynolds number is defined as:

$$\text{Re} = \frac{\text{Inertial force}}{\text{Viscous force}} = \frac{u \cdot Lc}{v} \tag{S11}$$

where u is the characteristic velocity, Lc is characteristic length (depending on geometry), ν is the kinematic viscosity (ν=μ/ρ). The form of Reynolds number calculation depends on the system geometry. The key is in understanding the definition of characteristic length Lc, depending entirely on the type of flow and geometry, in Reynolds number.

Case 1: Flow over a flat plate.

- Characteristic length: L length of the plate
- Velocity: free-stream velocity over the plate
- Used when analyzing boundary layer development in open/external flow
- Appropriate to use: $\text{Re} = \frac{\rho \cdot u \cdot L_c}{\mu}$

Case 2: Flow inside a pipe or channel.

- Characteristic length: hydraulic diameter
- Velocity: average (cross-sectional) velocity
- Used for internal (confined) laminar or turbulent flow
- Appropriate to use: $\text{Re} = \frac{\rho \cdot u \cdot d_h}{\mu}$

We are modeling a flow-type electrochemical reactor with defined channel dimensions, where flow is bound on all sides (internal flow). This matches pipe/channel flow, not flat-plate flow. Using L instead of $d_h$ would incorrectly model the wrong physical behavior — it would treat your system like a boundary layer forming on a single surface instead of a fully developed internal flow with resistance on all sides. So, we use $\text{Re} = \frac{\rho \cdot u \cdot d_h}{\mu}$, where $d_h$ is the hydraulic diameter ($d_h = \frac{4A}{P}$), u is the mean velocity of the electrolyte in the channel.

Schmidt number calculation:

$$S_c = \frac{\mu}{\rho D} \tag{S12}$$

Sherwood number calculation (laminar Graetz approximation):

$$S_h = 1.86 \cdot \left( \frac{R_e \cdot S_c \cdot d_h}{L} \right)^{1/3} \tag{S13}$$

Mass transfer coefficient calculation:

$$K_m = \frac{Sh \cdot D}{d_h} \tag{S14}$$

where $k_m$ is the mass transfer coefficient (m $s^{-1}$), $d_h$ is the hydraulic diameter, D is the diffusivity of the wastewater.

Diffusion layer thickness, which represents effective mass transfer resistance in the flow system, was calculated using:

$$\delta = \frac{D}{k_m} \tag{S15}$$

This combined model appropriately describes ion transport under strong electric fields and steep concentration gradients near reactive interfaces, offering a more physically accurate representation than purely diffusive models. This framework supports quantitative analysis of $NH_4^+$ conversion and ammonia gas evolution in electrified membrane or cathodic systems under flow conditions.

## Text S4. RHE calibration of reference electrode

The Ag/AgCl reference electrode was calibrated for simulated ammonium containing wastewater at room temperature (25 °C). It was calibrated with respect to reversible hydrogen electrode (RHE). The calibration was performed in the high purity hydrogen saturated electrolyte with Pt wires as the working electrode and counter electrode.(*64-66*) Cyclic voltammetry curves were run at a scan rate of 1 mV·$s^{-1}$, and the average of the two potentials at which the current crossed zero was taken to be the thermodynamic potential for the hydrogen electrode reactions.

$$E_{\mathrm{RHE}} = E_{\mathrm{AgCl}} + 0.743\ \mathrm{V} \tag{S16}$$

## Text S5. Calculation of detection efficiency of RD

The RRDE used in this research consisted of a glassy carbon disk with radius ($r_1$) of 2.5 mm,

and a Pt ring with inner ($r_2$) and outer radius ($r_3$) of 3.0 mm and 3.75 mm, respectively. In addition, the detection efficiency (ND) of $IrO_x$ ring electrode was calculated by the following equation, which is 0.27 in this work. All measurements were conducted at rotation speed of 1600 rpm. The detailed calculation was (*52*):

$$N_D = 1 - \frac{1}{6}F\left[\left(\frac{r_2}{r_1}\right)^3 - 1\right] - \frac{2}{3}F\left[\left(\frac{r_2 + r_3}{2r_1}\right)^3 - 1\right] - \frac{1}{6}F\left[\left(\frac{r_3}{r_1}\right)^3 - 1\right] \quad \text{(S17)}$$

$$F[\theta] = \frac{3^{\frac{1}{2}}}{4\pi}\ln\left[\frac{\left(1+\theta^{\frac{1}{3}}\right)^3}{1+\theta}\right] - \frac{3}{2\pi}\tan^{-1}\left(\frac{2\theta^{\frac{1}{3}} - 1}{3^{\frac{1}{2}}}\right) + \frac{1}{4} \quad \text{(S18)}$$

**Text S6. Finite-element multiphysics (FEM)**

Electric currents. Charge conservation and constitutive relations were written as:

$$\nabla \cdot J = Q_{j,v} \quad \text{(S19)}$$

$$J = \sigma E + \frac{\partial D}{\partial t} + J_e \quad \text{(S20)}$$

$$E = -\nabla v \quad \text{(S21)}$$

where $J$ is current density, $Q$ is volume charge density, $\sigma$ is electrical conductivity, $D$ is electric displacement, and $v$ is electric potential.

Transport of diluted species. Ionic transport followed the advection-diffusion-migration (Nernst-Planck) statement:

$$\frac{\partial c_i}{\partial t} + \nabla \cdot J_i = R_i \quad \text{(S22)}$$

$$J_i = -D_i \nabla c_i - z_i u_{m,i} F c_i \nabla V + c_i u \quad \text{(S23)}$$

where $c_i$ is the concentration of species $i$, $J_i$ is the flux including diffusion flux and migration flux, $u$ is the fluid velocity; $R_i$ is the reaction rate, $D_i$ is diffusion coefficient, $z_i$ is charge number, $u_{m,i}$ is ionic mobility, F is Faraday's constant, and $V$ is electrical potential.

Bubbly flow. Gas-liquid hydrodynamics were modeled via volume averaged mass and

momentum balances for liquid and gas phase fractions:

$$\rho_l \frac{\partial u_l}{\partial t} + \rho_l (u_l \cdot \nabla) u_l = \nabla \cdot [-pI + K] + \varphi_l \rho_l g + F \tag{S24}$$

$$K = u_l (\nabla u_l + (\nabla u_l)^T) \tag{S25}$$

$$\frac{\partial \varphi_g \rho_g}{\partial t} + \nabla \cdot N_{\rho_g \varphi_g} = -m_{gl} \tag{S26}$$

$$\varphi_g \rho_g = rho_{g,eff} \tag{S27}$$

$$N_{\rho_g \varphi_g} = \varphi_g \rho_g u_g \tag{S28}$$

$$u_g = u_l + u_{slip} \tag{S29}$$

where $\rho_l$ is liquid phase density, $u_l$ is liquid phase velocity, $\rho_l \nabla \cdot u_l = 0$, $u_l = u$ , $p$ is pressure, $I$ is unit tensor, $K$ is deviatoric stress tensor, $\varphi_l$ is liquid phase volume fraction, $g$ is gravitational acceleration, $F$ is volume force, $u_l$ is dynamic viscosity of liquid phase, $\varphi_g$ is bubble phase volume fraction, $\rho_g$ is bubble phase density, $m_{gl}$ is interphase mass transfer term, $rho_{g,eff}$ is effective density of bubble phase, $N_{\rho_g \varphi_g}$ is bubble phase mass flux, $u_g$ is bubble phase velocity, $u_{slip}$ is slip velocity (the relative velocity of the bubble relative to the liquid phase). The detailed parameters are listed in **Table S21**.

## <u>Text S7. DFT calculation</u>

We employed the Vienna Ab Initio Simulation Package (VASP) to perform the DFT calculations and applied the generalized gradient approximation (GGA) using the Perdew-BurkeErnzerhof (PBE) formulation. The projector augmented wave (PAW) pseudopotentials were used to describe the ionic cores with a plane wave basis set and kinetic energy cutoff of 600 eV to account for the valence electrons. Partial occupancies of the Kohn−Sham orbitals were allowed using the Gaussian smearing method and a width of 0.05 eV. The energy was considered self-consistent when the energy change was smaller than $10^{-6}$ eV. Geometry optimization was considered converged when the force change was smaller than 0.02 eV·Å-1.

Grimme's DFT-D3 methodology was used to describe dispersion interactions. To perform crystal orbital Hamilton population (COHP) calculations and summarized bonding information, we used the LobsterPy package that automatically analyzes LOBSTER ICOHPLIST output files. During the relaxation, the Brillouin zone with a 2 × 2 × 1 Gamma centered grid was used. The 20 Å vacuum layer was normally added to the surface to in order to separate the surface slab from its periodic duplicates. Spin polarized calculations were performed for this calculation. The Gibbs free energy change (ΔG) was evaluated based on the computational hydrogen electrode model, which takes one-half of the chemical potential of gaseous hydrogen under standard conditions as the free energy of the proton−electron pairs. ΔG was calculated using the following equation: $\Delta G = \Delta E + \Delta E_{ZPE} - T\Delta S$ where $\Delta E$, $\Delta E_{ZPE}$, and $\Delta S$ are the reaction energy obtained from the DFT calculation, the correction of the zero-point energy, and the change in the simulated entropy, respectively; $T$ is the temperature (T=298.15K).

**Text S8. Fabrication and hydrophobic treatment of $SiO_2$ microspheres**

Synthesis of small sized $SiO_2$ microspheres (0.08±0.01 μm). The $SiO_2$ microspheres were synthesized by the Stöber method.(*67-69*) In a 250 mL round-bottom flask equipped with a PTFE stir bar, ethanol (pre-measured to bring the total reaction volume to 200 mL after all additions), deionized water (7.2 mL), and ammonia solution (Aladdin, 25%-28%, AR, 13.8 mL) were combined at 25 °C and stirred at 300 rpm for 5 min. Tetraethyl orthosilicate (TEOS, Aladdin, >99%, GC, 8.9 mL) was then added rapidly (within 30 s) under continuous stirring to initiate hydrolysis and condensation. The mixture was allowed to react without further intervention for 12 h at 25 °C. The resulting colloidal silica was collected by centrifugation, redispersed in ethanol, and washed three times to remove residual ammonia and oligomers. The powder was dried at 50 °C overnight.

Synthesis of middle sized $SiO_2$ microspheres (0.42±0.05 μm). Ethanol, deionized water (27.0 mL) and ammonia solution (6.9 mL) were combined in a 250 mL flask at 25 °C with stirring (300-600 rpm). TEOS (11.1 mL) was added in one portion under stirring. The suspension was aged for 12 h at 25 °C. Particles were isolated by centrifugation, washed three times with ethanol, and dried at 50 °C overnight.

Synthesis of large sized $SiO_2$ microspheres (0.90±0.07 μm). Seed particles prepared as above were retained in their mother liquor containing ammonia. At 25 °C under stirring, a growth solution consisting of TEOS and water (molar ratio 1:2) in ethanol was added slowly (dropwise over 10 min) to the seed dispersion while maintaining the presence of ammonia solution. After each addition, the mixture was allowed to react to completion (typically 2 h) before the next dose. Additions were repeated until the desired diameter was achieved, avoiding large single doses that can cause secondary nucleation. Products were purified and dried as described above.

After washing, particles were stored as dry powders. All steps were carried out at room temperature (25 °C) unless otherwise stated.

Hydrophobic treatment of $SiO_2$ microspheres. The obtained $SiO_2$ microspheres with different

sizes were all coupled with silane for hydrophobic treatment.*(69)* In detail, dried $SiO_2$ microspheres (1.0 g) were resuspended in 20 mL of anhydrous ethanol containing 2 mL of ammonia solution. A salinization solution, 100 mL ethanol with 1 vol% dodecyltrimethoxysilane (Aladdin, >93%, GC) and 9 vol% trimethoxy(octadecyl)silane (Macklin, >95%), was then introduced to the dispersion. The mixture was stirred magnetically at 500 rpm at ambient temperature for 6 h to allow surface coupling. The hydrophobized $SiO_2$ was isolated by centrifugation, washed three times with ethanol, and dried under vacuum overnight.

## Text S9. Scope, functional unit, and system boundaries

Framework selection criteria: (i) comparability across nitrogen removal/recovery routes—hence FU-A (1 kg-N recovered as $NH_3$); (ii) engineering traceability from performance (areal rate) to capacity and capital cost (rate → area → capital expenditure); (iii) separation of reactor versus auxiliary electricity to avoid optimistic energy accounting; (iv) cradle-to-gate LCA with substitution credit for displaced fossil $NH_3$ to reflect circular-nitrogen benefits; (v) sensitivity-ready structure (lifetime, prices, grid mixes) to generalize across sites.

The assessment was conducted using FU-A: 1 kg-N recovered as $NH_3$ as the functional unit (Ammonia yield per unit nitrogen is determined by molecular weight ratio: $m_{NH_3} = \frac{17}{14} m_N = 1.214\ kg\ NH_3\ per\ kg\text{-}N$). The system boundary includes wastewater influent, ion transport and reduction at the cathode, and recovery of ammonia as a pure gas. No chemical dosing is required in the base case, and the process is fully driven by electrical energy. Material contributions from membrane and electrode manufacture are incorporated through lifetime-based amortization. A cradle-to-gate perspective is adopted, with displacement credit for avoided fossil $NH_3$ production.

The electrified-membrane process is electricity-driven, modular, and areal-rate limitedly matched to a rate-to-area sizing methodology. FU-A aligns with fertilizer and nutrient-circularity objectives, enabling revenue and avoided-burden accounting in both TEA and LCA. Cradle-to-gate boundaries capture the dominant contributions (electricity, materials) while allowing optional additions (chemicals, logistics) where relevant.

System boundaries include (i) influent handling; (ii) electrified-membrane module (ion transport and cathodic $NH_4^+$→$NH_3$) and $NH_3$ gas recovery; (iii) balance-of-plant (BOP) electricity (pumps & gas handling); (iv) periodic membrane/electrode replacement; (v) avoided fossil $NH_3$ credit.

## Text S10. Life-Cycle Assessment (LCA)

Framework selection criteria: (i) attributional cradle-to-gate scope to quantify operational and material burdens at the point of product $NH_3$; (ii) avoided-burden (substitution) credit for

displaced fossil $NH_3$ recognizing commodity equivalence; (iii) explicit electricity-related greenhouse gas emissions with location-dependent grid intensity; (iv) lifetime-scaling of embodied materials; (v) extensibility to eutrophication and acidification once inventories are available.

The product is $NH_3$, a fungible commodity; substitution is appropriate and conservative under standard consequential interpretations. Electricity is the dominant operating driver for electro-membrane systems; modeling grid intensity and auxiliary loads is necessary for accurate greenhouse gas emissions estimates. Lifetime scaling captures durability impacts without overfitting vendor-specific life cycle inventories.

Calculation Equations:

Electricity greenhouse gas emissions: $CO_{2,\text{ elec}} = SEC_N \times CI_{\text{grid}}$

Avoided fossil $NH_3$: $CO_{2,\text{ avoided}} = 1.214 \times EF_{NH_3,\text{ fossil}}$

where $EF_{NH_3,\text{ fossil}} \approx 2.4$ t-$CO_2$ t$^{-1}$-$NH_3$.

Net greenhouse gas emissions balance: $\Delta CO_2 = CO_{2,\text{ elec}} - CO_{2,\text{ avoided}}$

## Text S11. Techno-Economic Analysis (TEA)

Framework selection criteria: (i) use of measured $SEC_N$ and explicit auxiliary power to represent operational reality; (ii) rate-to-area scaling to map laboratory areal productivity to commercial module size and capital intensity; (iii) explicit separation of variable costs (electricity) and lifetime-dependent operating expenditure (*OPEX*) to expose durability risks; (iv) levelization via capital recovery factor (*CRF*) to compare scales and financing assumptions; (v) break-even product pricing to enable market feasibility checks under uncertainty.

Electro-membrane modules scale primarily with active area and duty factor, not with reactor volume alone. The TEA ties performance directly to capital expenditure and operating expenditure, capturing the physics of transport-limited recovery and the economics of modular deployments. The approach is location-agnostic regarding power source and can integrate grid tariffs or PPA structures.

Calculation Equations:

Electricity cost: $\text{Cost}_{\text{elec}} = SEC_{\text{N}} \times p_{\text{elec}}$

where $SEC_{\text{N}}$ is the specific energy consumption for the separated nitrogen, $p_{\text{elec}}$ is the price of electricity.

Revenue (pure $NH_3$ gas): $\text{Rev}_{NH_3} = 1.214 \times p_{NH_3}$

where $\text{p}_{NH_3}$ is the market price of $NH_3$.

Net operating margin (NOM): $\mathrm{NOM} = \mathrm{Rev}_{NH_3} - \mathrm{Cost}_{\mathrm{elec}}$

Capacity sizing (linking performance to capital expenditure (*CapEx*)):

$$A_{\mathrm{act}} = \frac{Q_N}{24 r_N CF}$$

$$CapEx_{\mathrm{module}} = A_{act} \times C_A$$

<u>Levelized cost of recovered nitrogen (*LCN*) and $NH_3$.</u>

$$LCN = \frac{CRF \times CapEx_{\mathrm{total}} + OPEX_{\mathrm{annual}}}{M_{N,\mathrm{annual}}}$$

$$LCO - NH_3 = \frac{LCN}{1/1.214}$$

<u>Break-even $NH_3$ price (without tax, without logistics).</u>

$$p_{NH_3,BE} = \frac{\mathrm{Cost}_{\mathrm{elec}} + (CapEx \text{ amort. per kg-N})}{1.214}$$

**<u>Text S12. Demo at residential community</u>**

<u>Representative community (Base case).</u> This document provides complete calculation instructions and parameters for a single-building deployment of an electrified membrane reactor that recovers ammonia and generates chlorine as a co-product. Two ammonia recovery efficiencies are considered: (1) 54.8% ammonia recovery, (2) 95.1% ammonia recovery. Results are reported in two complementary views: (i) the target-recovery view (using the specified recoveries), and (ii) the area-limited view (what the fixed membrane areas can deliver in 24 hours).

<u>Equations (daily, then annual).</u>

1) Urine flow ($m^3$ $day^{-1}$) = population × 1.5 L $person^{-1}$ $day^{-1}$ × (1 $m^3$/1000 L).

2) N load (kg-N $day^{-1}$) = urine flow (L $day^{-1}$) × TAN (g-N $L^{-1}$) × (1 kg/1000 g).

3) Theoretical $NH_3$ (kg $day^{-1}$) = N load × (17/14).

4) Target-recovery $NH_3$ (kg $day^{-1}$) = Theoretical $NH_3$ × recovery%.

5) Area-limited $NH_3$ (kg $day^{-1}$) = Areal rate (kg-$NH_3$ $m^{-2}$ $h^{-1}$) × area ($m^2$) × 24 h.

6) Chlorine (kg $day^{-1}$) = [$NH_3$ mass (kg $day^{-1}$) / (17 g $mol^{-1}$)] × $Cl_2$:$NH_3$ × 70.906 g $mol^{-1}$.

7) Reactor energy (kWh $day^{-1}$) = SEC (kWh $kg^{-1}$-$NH_3$) × $NH_3$ mass (kg $day^{-1}$) + pumps (kWh $day^{-1}$).

8) Annualize by multiplying daily values by 365.

9) Revenue (\$ year$^{-1}$) = ($NH_3$ t year$^{-1}$ × price) + ($Cl_2$ t year$^{-1}$ × price).

10) Electricity cost (\$ year$^{-1}$) = kWh year$^{-1}$ × \$ kWh$^{-1}$.

11) Gross margin (\$ year$^{-1}$) = revenue - electricity cost.

12) Net after membrane (\$ year$^{-1}$) = gross margin - (capital expenditure / lifetime (years)).

Notes.

• Safety & permitting: Prefer on-site scrubbing of chlorine to sodium hypochlorite for storage and use; install gas detection and dedicated ventilation per local code.

• Operations: Batch scheduling can follow building diurnal load; include a small buffer tank to smooth weekday/weekend variability (for offices).

• Integration benefits not monetized here: avoided nitrogen surcharges to the utility; avoided purchase of disinfectants if NaOCl is generated; avoided odor controls.

## Text S13. Product quantification

The separated $NH_3$ was collected and dissolved in acid solution to form ammonium ions, diluted to the appropriate detection range, and detected by indophenol blue method. In detail, 2 mL of electrolyte was removed from the cathodic chamber. Then, 2 mL of a 1 M NaOH solution containing 5 wt% of salicylic acid ($C_7H_6O_3$) and 5 wt% of sodium citrate dihydrate ($C_6H_5Na_3O_7 \cdot 2H_2O$) was added into this solution, followed by further addition into 1 mL of 0.05 M NaClO and 0.2 mL of 1 wt% Sodium nitroferricyanide ($C_5FeN_6Na_2O \cdot 2H_2O$) solution. The UV-Vis absorption spectrum was measured at λ=655 nm after standing at room temperature for 2 h. The concentration-absorbance curve was calibrated using standard $NH_4Cl$ solution with a series of concentrations. The fitting curve ($y = 0.1397\,x + 0.0025$, $R^2 = 0.999$) shows good linear relation of absorbance value with $NH_3$ concentration under three independent calibrations (**Fig. S68**).

The separated $Cl_2$ was collected and dissolved in base solution to form free chlorine (hypochlorous acid and hypochlorite ion), diluted to the appropriate detection range, and measured by USEPA DPD Method (HACH Method 8021). In detail, DPD free chlorine reagent powder pillow was added to a 10 mL diluted water sample and swirled in the sample cell for 20 seconds to mix. The UV-Vis absorption spectrum was measured at λ=530 nm within 60 seconds of the reagent addition. The concentration-absorbance curve was calibrated using a series of standard free chlorine solutions. The fitting curve ($y = 0.1775x - 0.0057$, $R^2 = 0.999$) shows good linear relation of absorbance value with $NH_3$ concentration under three independent calibrations (**Fig. S69**).

## Supplementary Figures

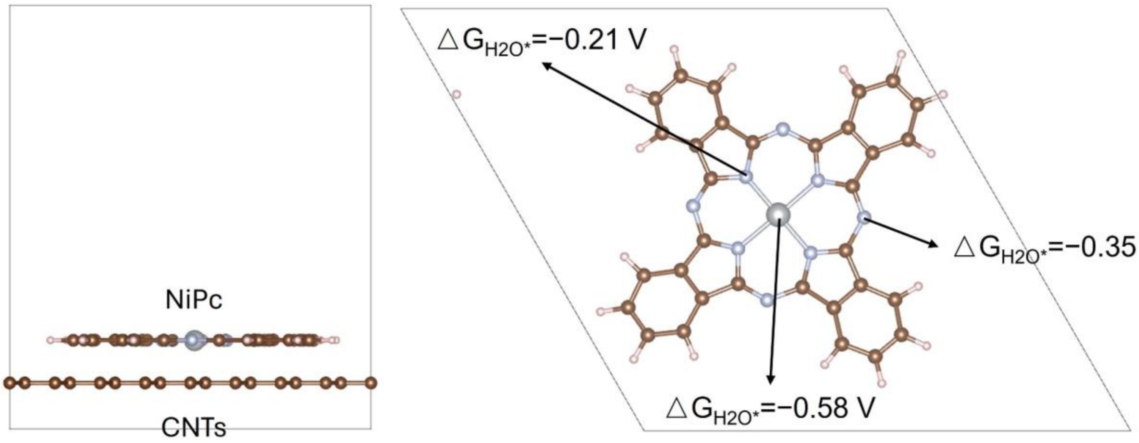


**Fig. S1. The free energy of $H_2O$ adsorption on NiPc/CNT.** The NiPc was placed above CNT.

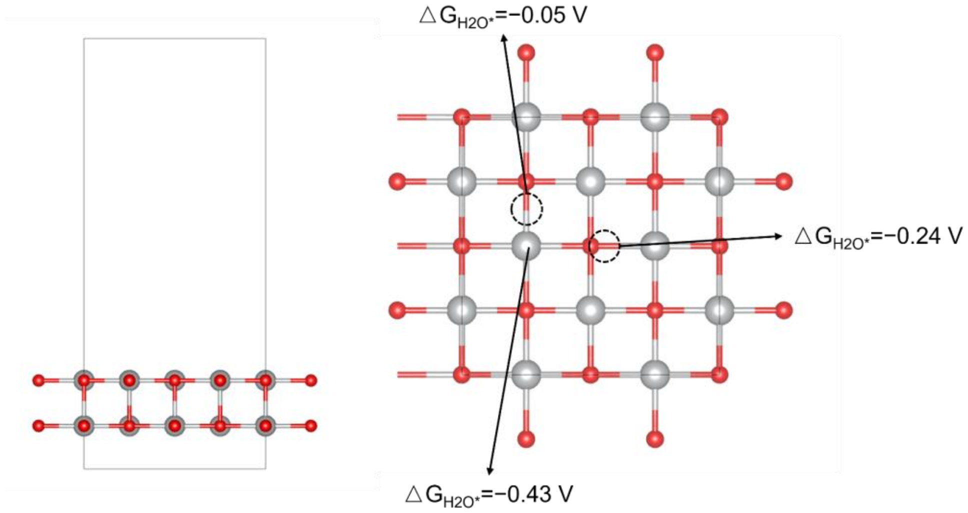


**Fig. S2. The free energy of $H_2O$ adsorption on NiO.** The NiO calculated here is [200], which is consistent with the main exposed crystal plane of NiO used in the subsequent experiments.

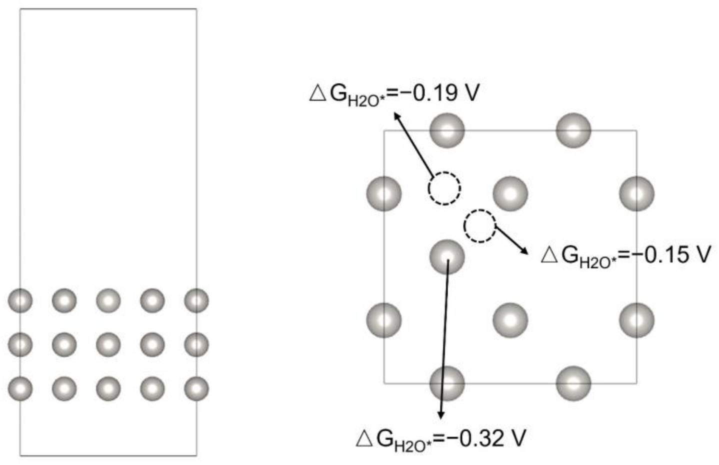


**Fig. S3. The free energy of $H_2O$ adsorption on Pt.** The Pt calculated here is [111], which is consistent with the main exposed crystal plane of Pt used in the subsequent experiments.

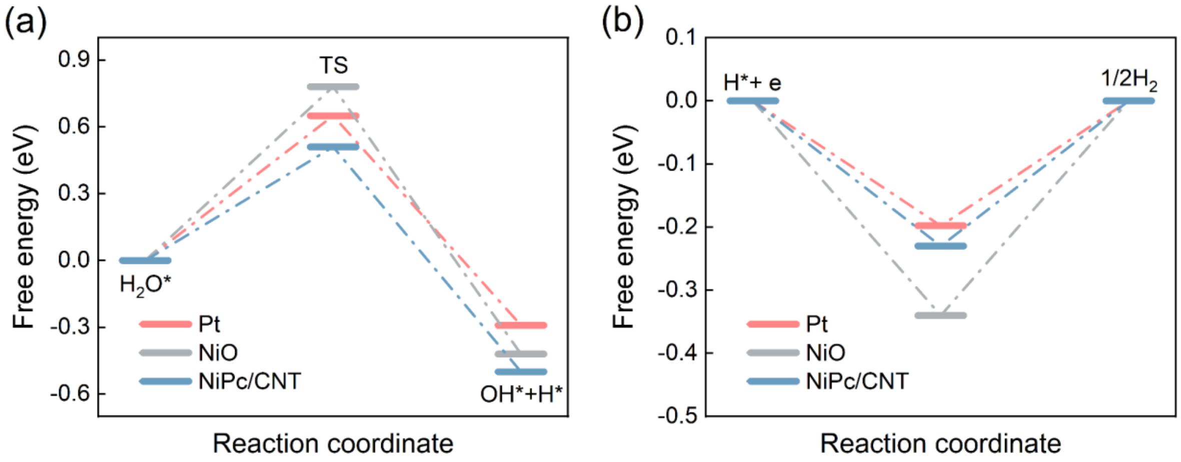


**Fig. S4. Gibbs free energy calculation.** The Gibbs free energy of NiPc/CNT, NiO, and Pt towards water dissociation (a) and hydrogen adsorption (b).

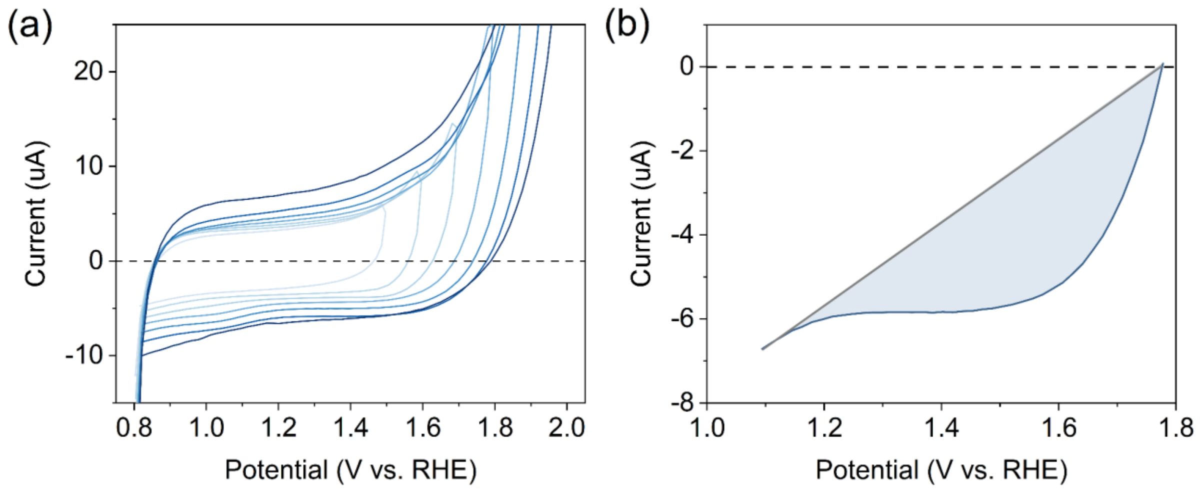


**Fig. S5. Active sites quantification by cyclic voltammetry tests.** (**a**) cyclic voltammetry curves of 2.8wt% NiPc/CNT under different potential intervals. Mass loading of NiPc/CNT is 0.5 $mg_{cat}$ $cm^{-2}$. Scan rate is 5 mV $s^{-1}$. (**b**) Reductive current of cyclic voltammetry curve within a potential interval of 0.8-2.0 V vs. RHE.

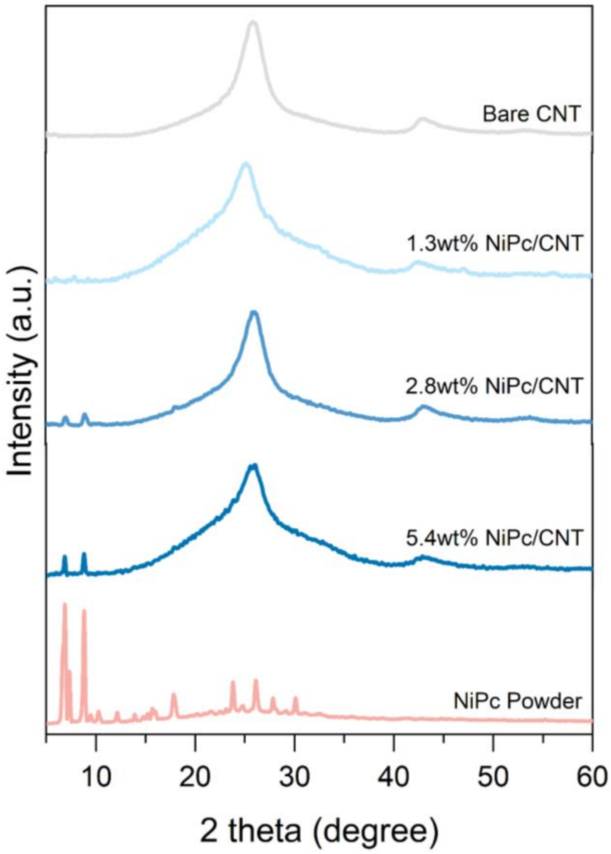


**Fig. S6. XRD characterizations.** XRD spectra of bare CNT powder, NiPc powder, and NiPc distributed on CNT (NiPc/CNT) with different loading weights.

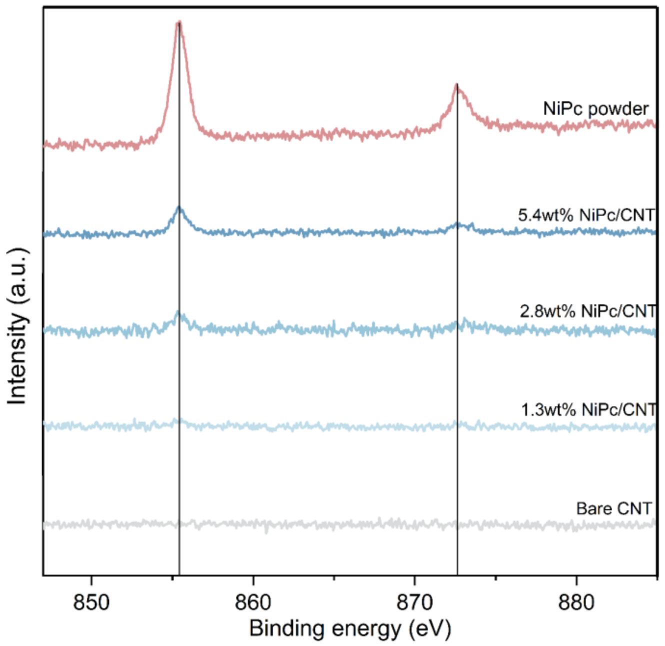


**Fig. S7. XPS characterizations.** Ni 2p XPS spectra of bare CNT powder, NiPc powder, and NiPc distributed on CNT (NiPc/CNT) with different loading weights.

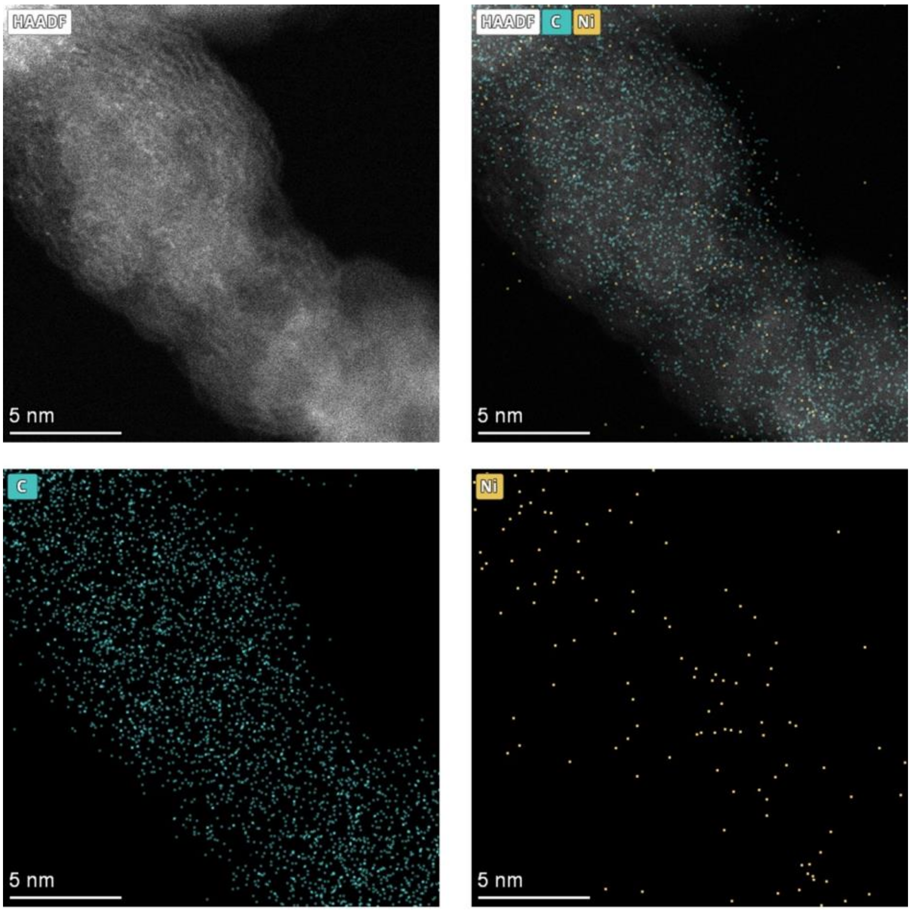


**Fig. S8. NiPc/CNT structure.** High-resolution HAADF-STEM image and corresponding EDX elemental mapping of NiPc/CNT. This figure corresponds to **Fig. 2B**.

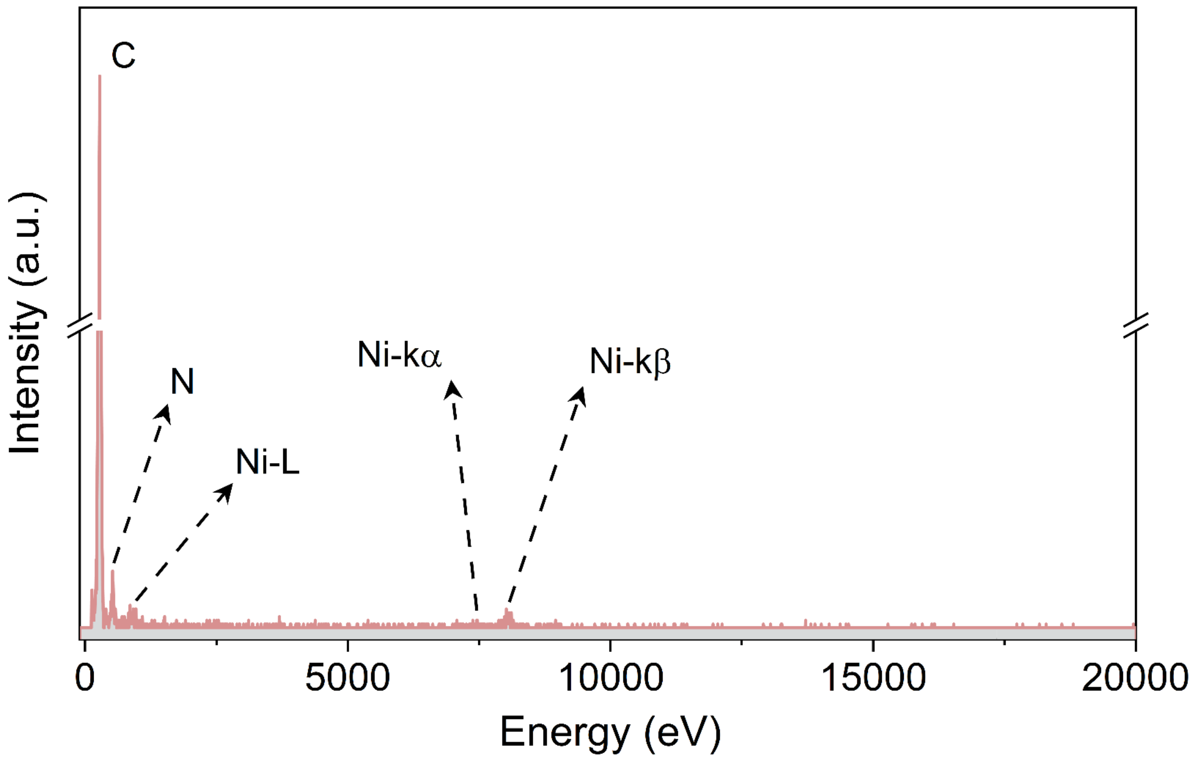


**Fig. S9. EDS characterization of the individual NiPc/CNT region.** Corresponding EDS spectrum acquired from the individual NiPc/CNT region in **Fig. 2B**.

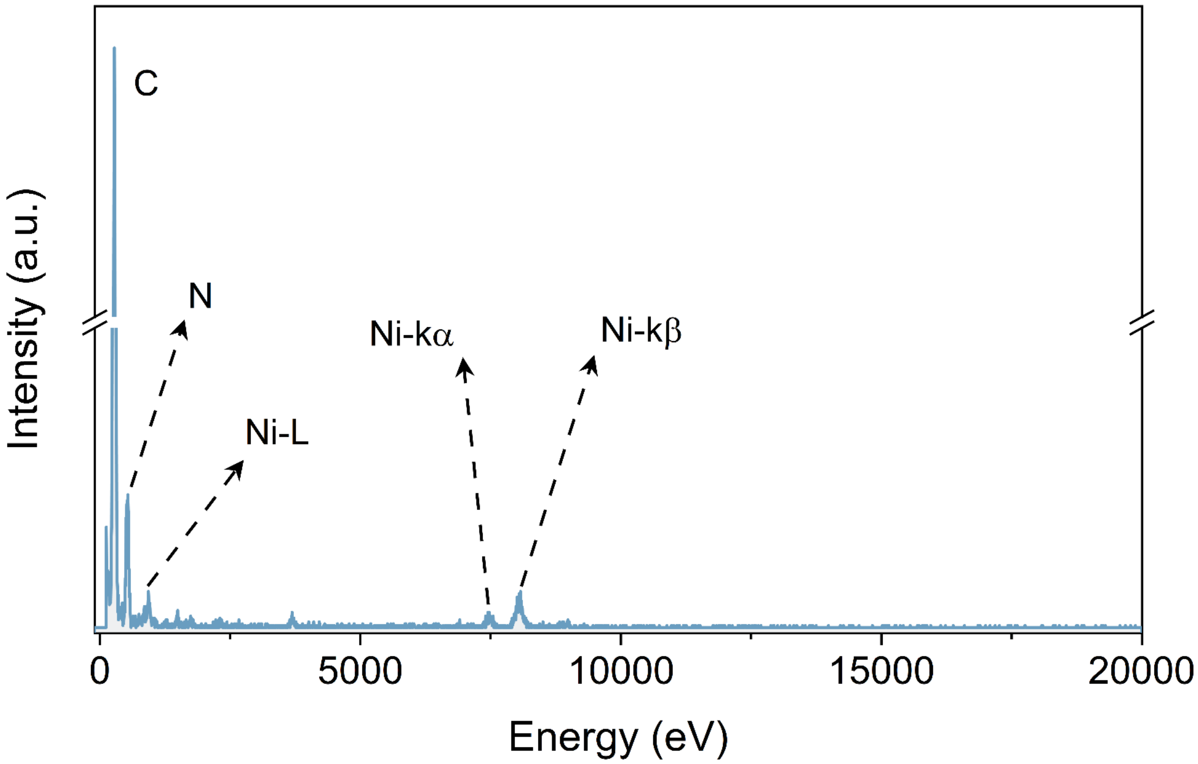


**Fig. S10. EDS characterization of the multiple NiPc/CNT regions.** Corresponding EDS spectrum acquired from the multiple NiPc/CNTs region in **Fig. 2C**.

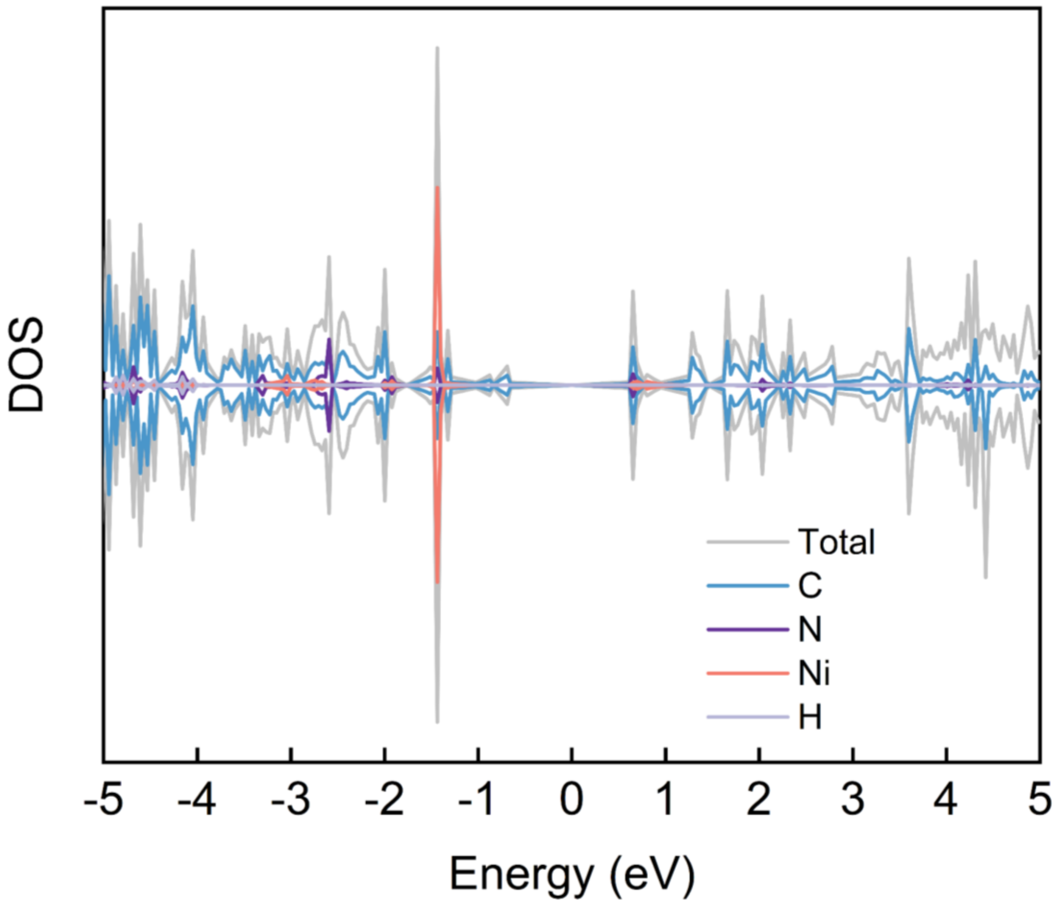


**Fig. S11. Electronic structure of NiPc/CNT.** Calculated density of states (DOS) of NiPc/CNT, showing the electronic structure of the hybrid NiPc/CNT system.

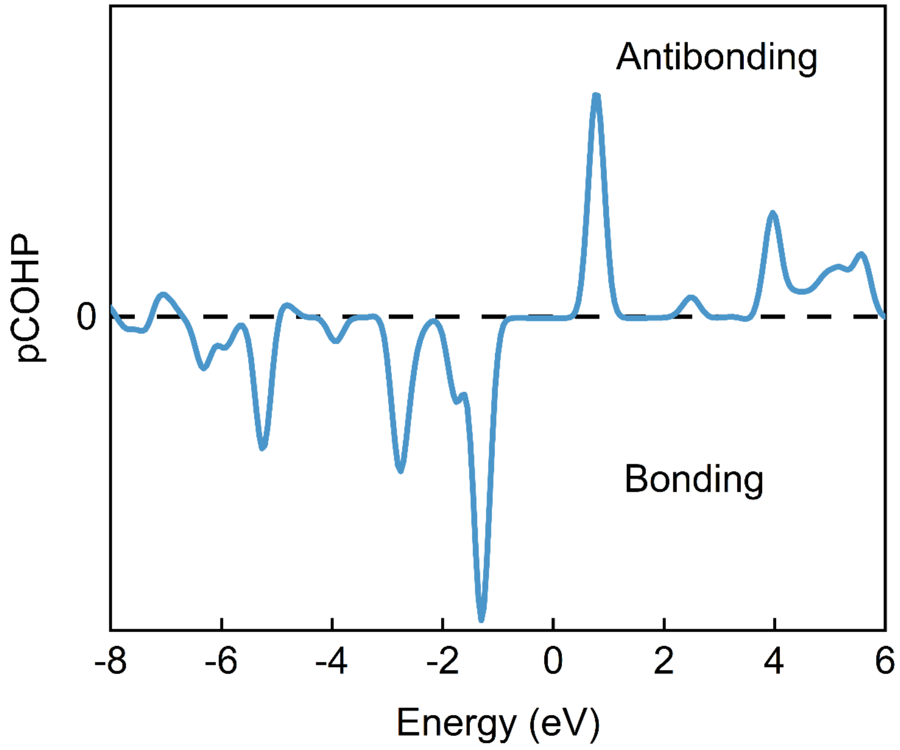


**Fig. S12. Bonding interactions in NiPc/CNT.** Predicted projected crystal orbital Hamiltonian population (pCOHP) analysis of NiPc/CNT, used to evaluate the interfacial interactions between NiPc and CNT.

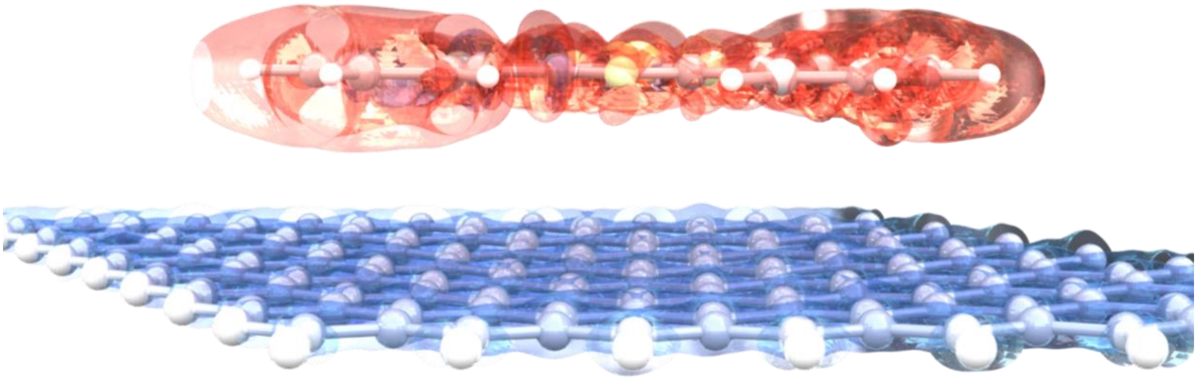

**Fig. S13. Differential charge diagram of NiPc/CNT.** Blue and red represent the depletion and accumulation of electrons, respectively.

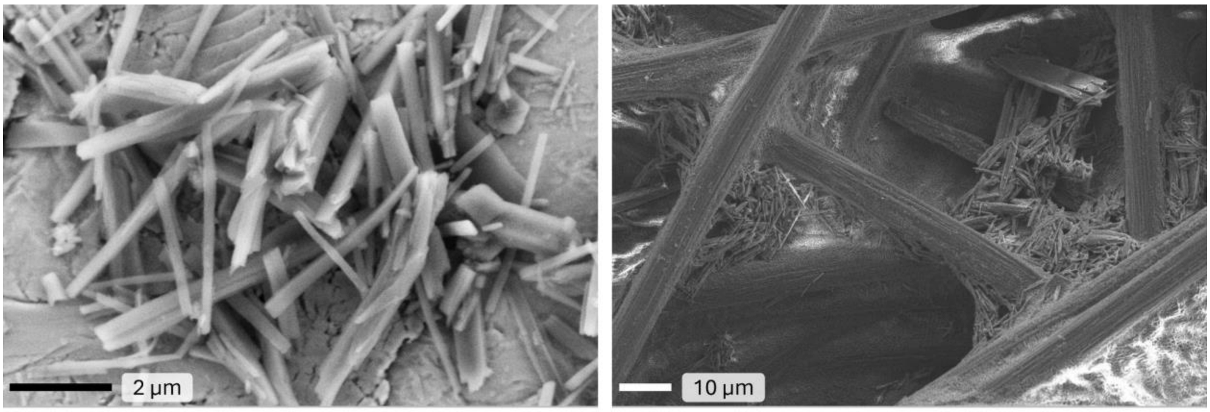


**Fig S14. SEM characterizations of NiPc particles.** SEM images of the NiPc particles (not distributed on CNT) loaded on the titanium foil (left) and the carbon fiber paper (right).

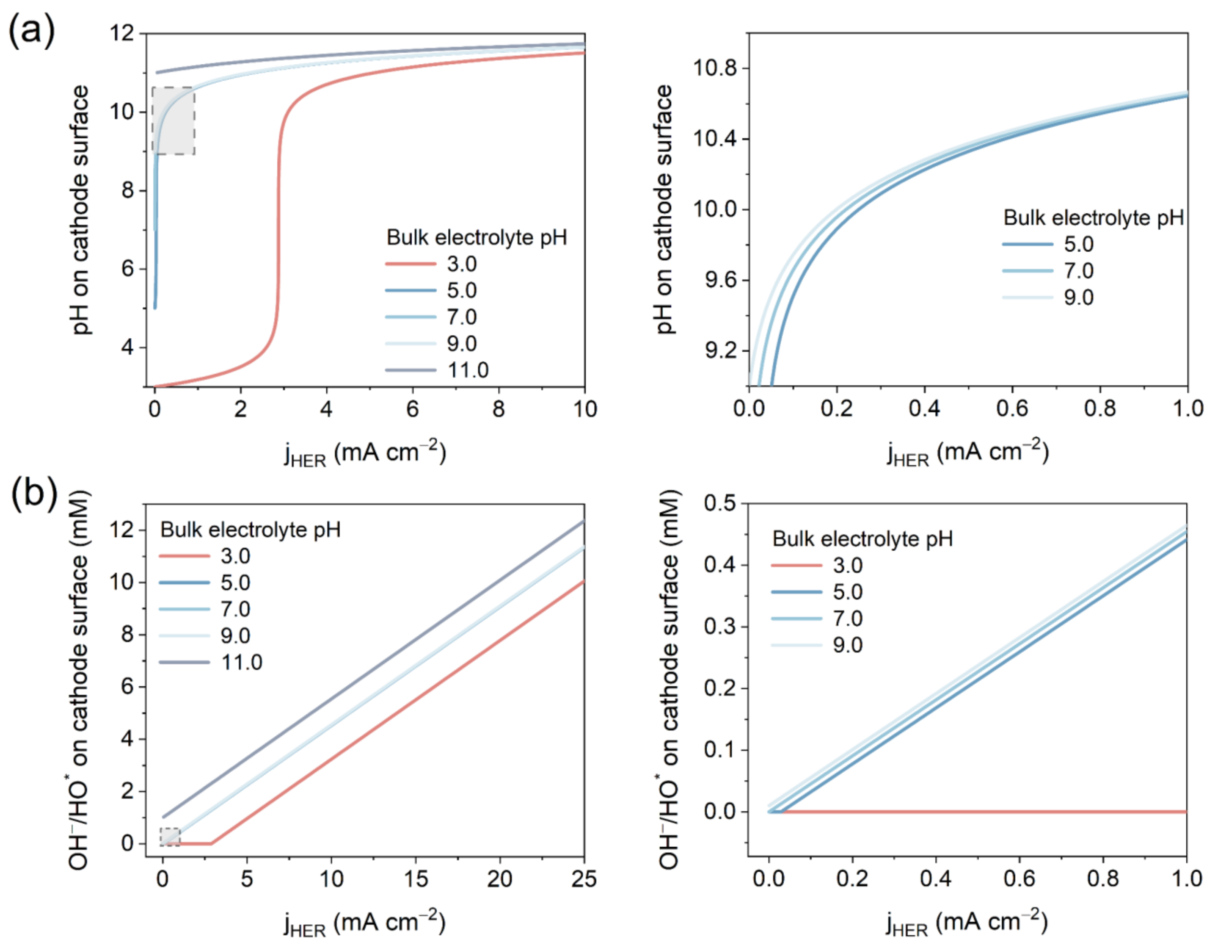


**Fig. S15. Interfacial theoretical calculations.** The interfacial pH (**a**) and the $OH^-/HO^*$ concentrations (**b**) on the cathode surface in relation to hydrogen evolution reaction current density ($j_{HER}$).

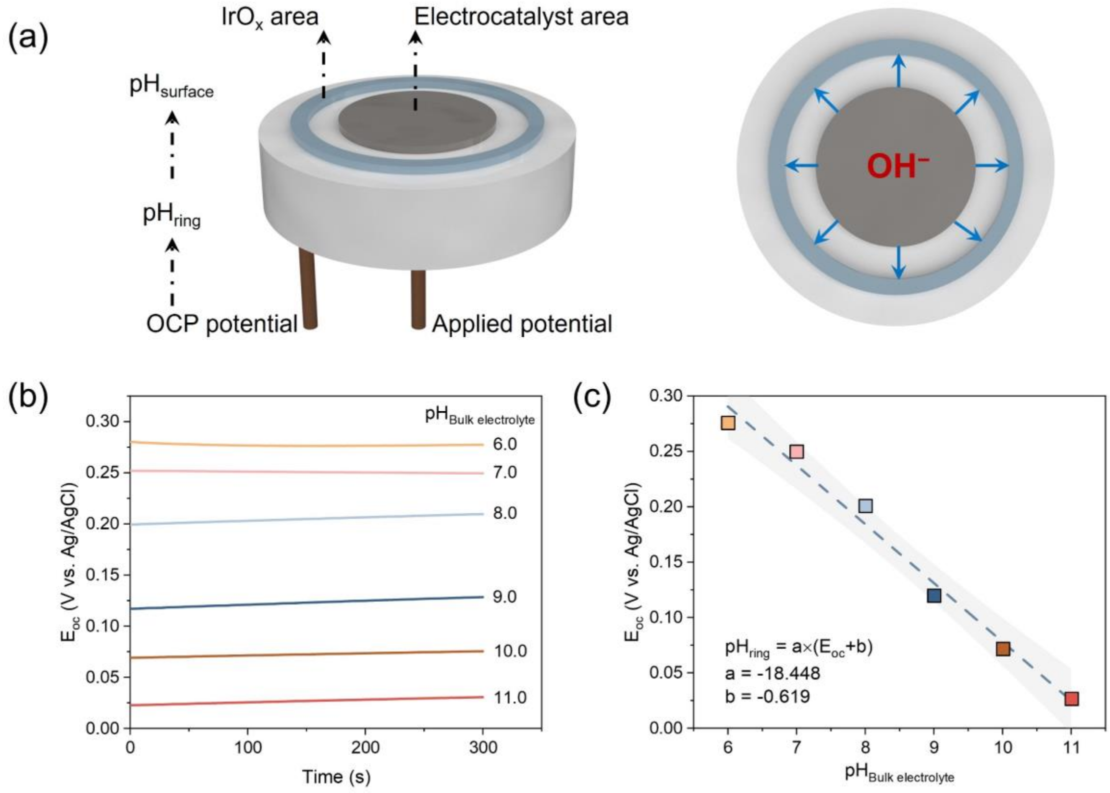


**Fig. S16. RRDE illustration and fitting curve.** (**a**) Schematic diagram for monitoring pH on the electrode surface ($pH_{surface}$) using an $IrO_x$-modified RRDE. During test, $OH^-$ generated on the catalyst surface ($C_{OH-,disk}$) was carried away by radial electrolyte flow and detected by the ring electrode. (**b**) Time and (**c**) pH dependence of open circuit potential ($E_{oc}$) for $IrO_x$ electrodeposited Pt-ring electrode. The measurement was performed in synthetic electrolyte, and the pH of the bulk electrolyte ($pH_{bulk\ electrolyte}$) was changed by adding $H_2SO_4$ or NaOH.

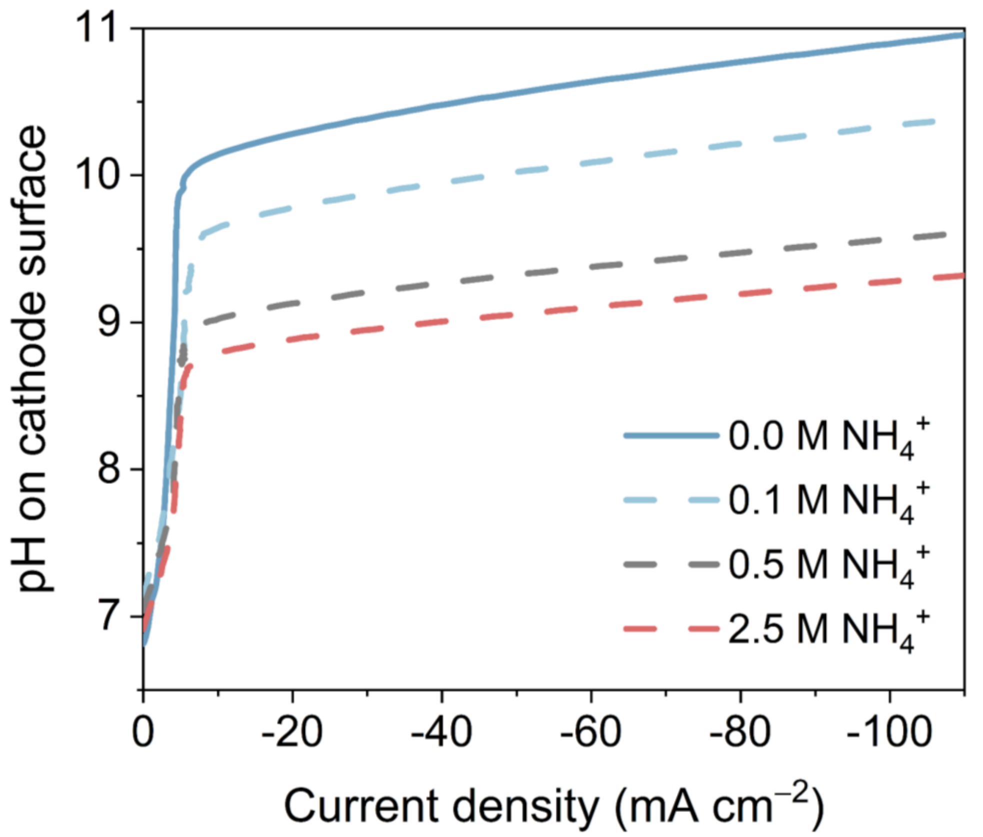


**Fig. S17. Cathode-surface pH.** Measured pH on the cathode surface with and without $NH_4^+$ ions, used to evaluate the effect of $NH_4^+$ on cathode-surface alkalinity under electrochemical operation.

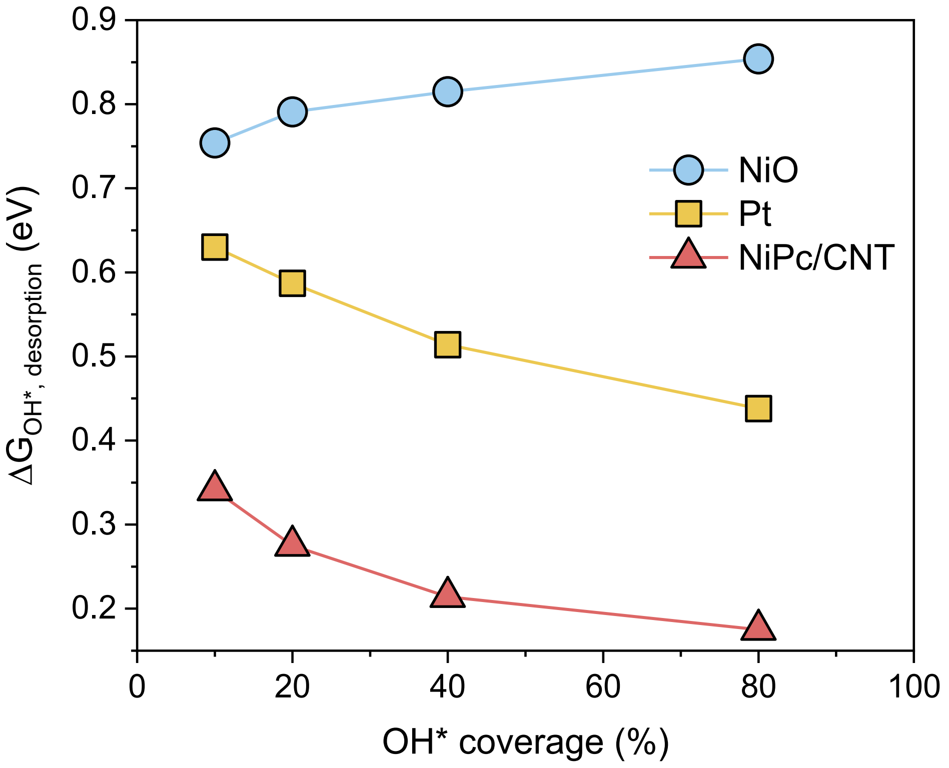


**Fig. S18. OH$^*$ desorption free energy.** Calculated OH$^*$ desorption free energy ($\Delta G_{OH^*,\ desorption}$) on the surface of NiO, Pt, and NiPc/CNT with different OH$^*$ coverage.

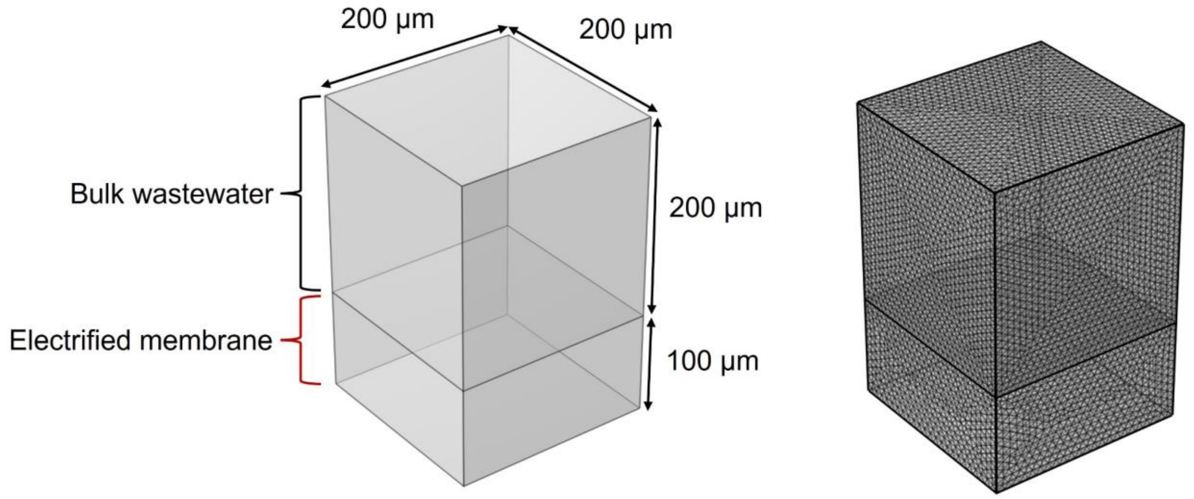


**Fig. S19. FEM model and mesh.** Schematic diagrams of the modelling (left) and meshing (right) for finite-element method (FEM) simulation.

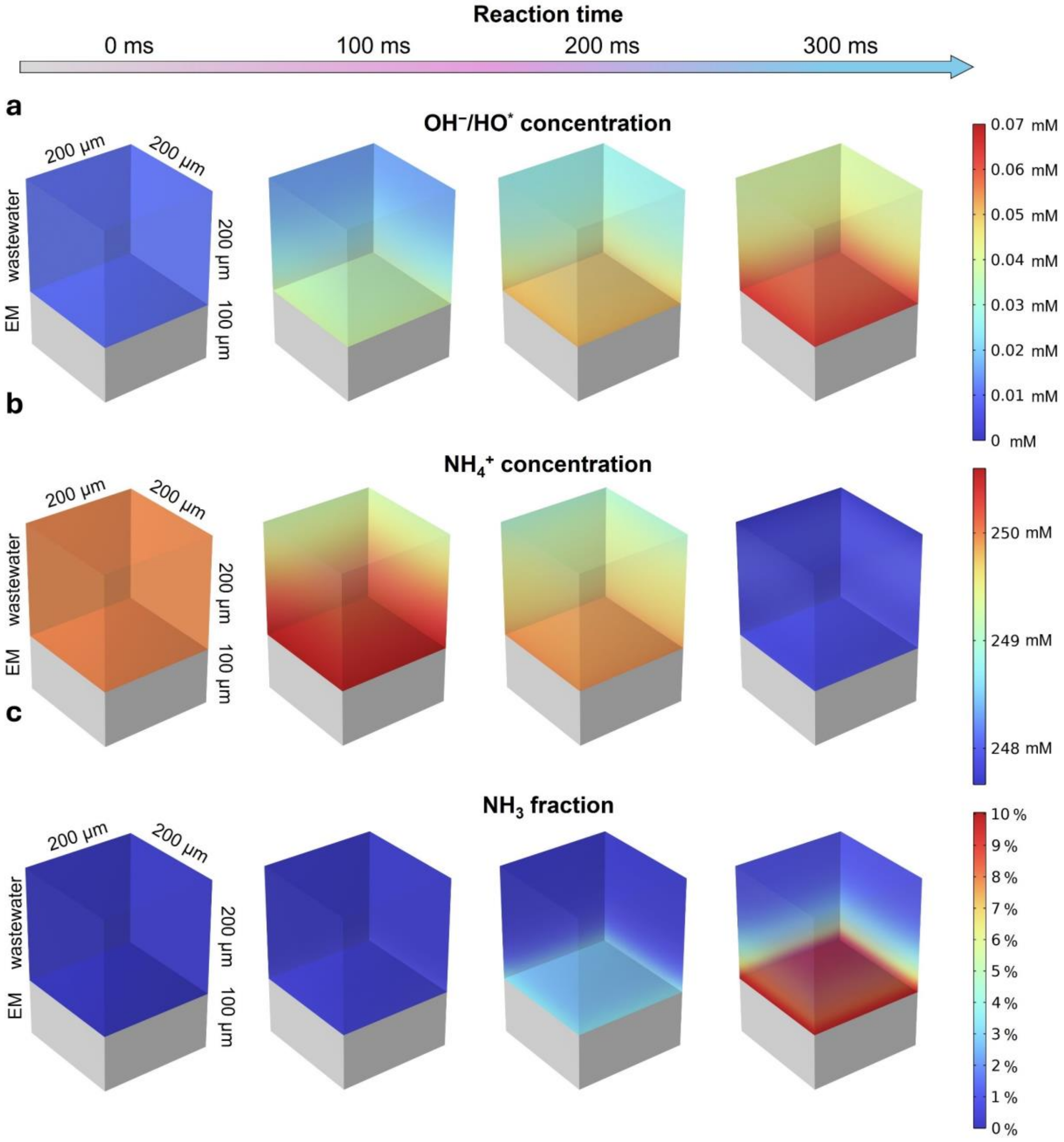


**Fig. S20. FEM simulations of plate electrified membrane surface.** (**a**) $OH^{-}/HO^{*}$ concentration, (**b**) $NH_4^+$ concentration, (**c**) $NH_3$ fraction at different distances from the membrane surface and reaction times (current density: 10 mA $cm^{-2}$). The initial $NH_4^+$ concentration in the simulated ammoniacal wastewater was 0.25 M.

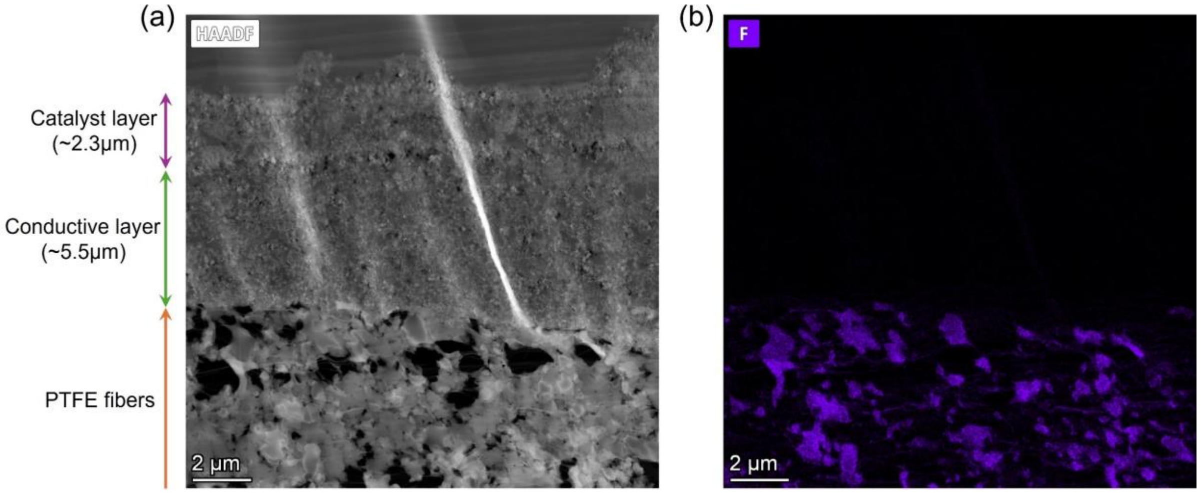


**Fig. S21. Cross-sectional view of EM-1.** HAADF-STEM (**a**) and EDS elemental mapping (**b**) images of EM-1.

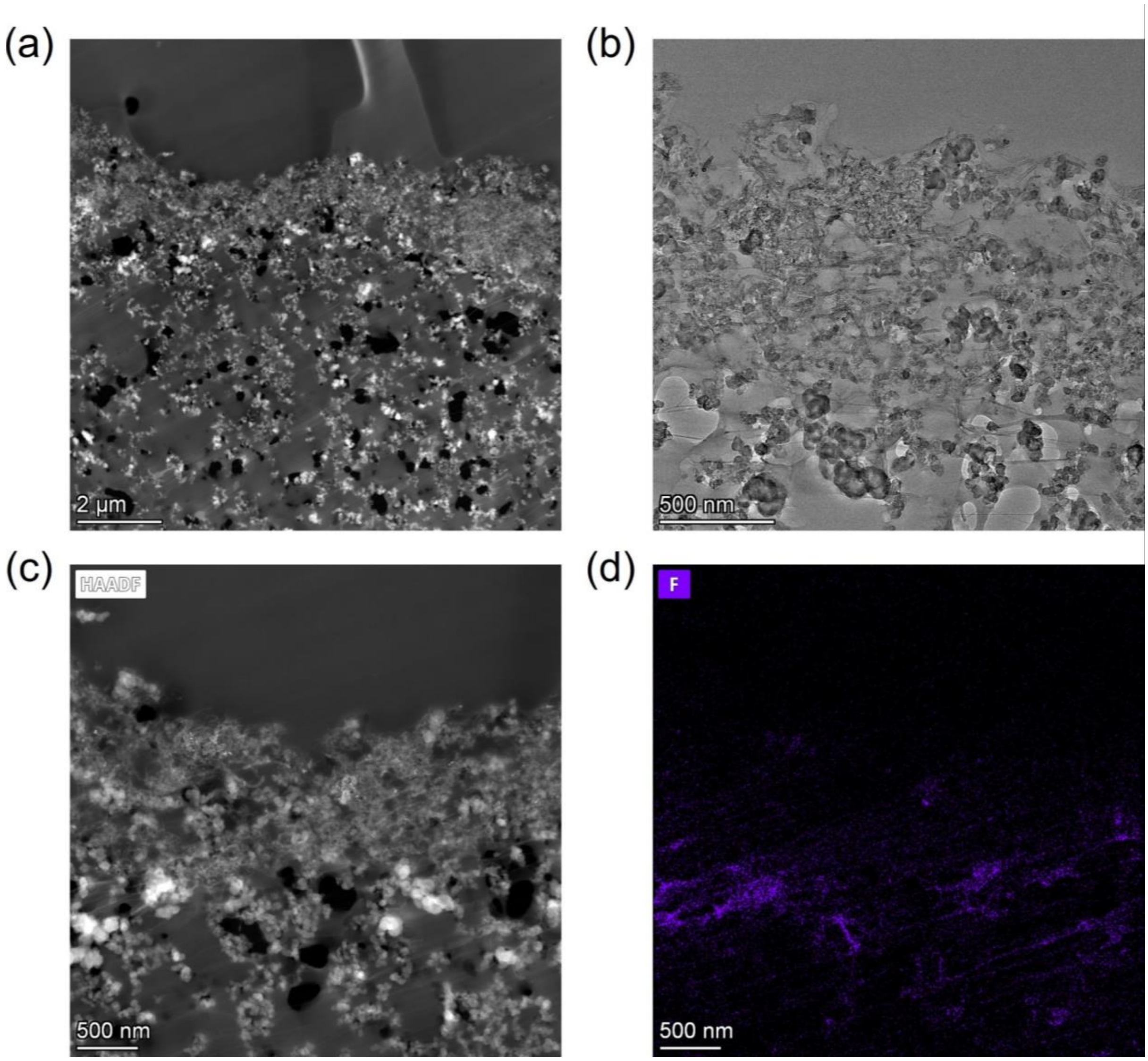


**Fig. S22. Cross-sectional view of EM-3.** HAADF-STEM images (**a-c**) and corresponding EDS elemental mapping image (**d**) of EM-3.

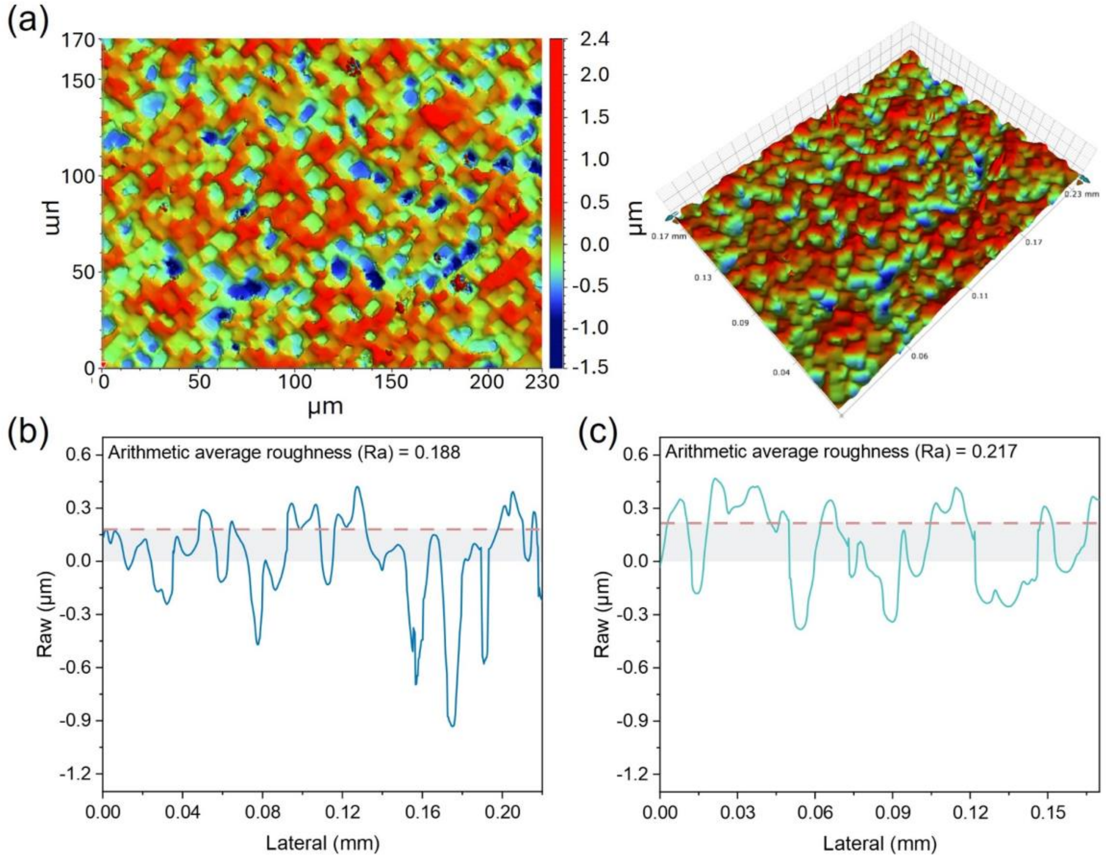


**Fig S23. Optical surface profiler measurement of EM-1.** (**a**) Two-dimensional and three-dimensional reconstructions. Raw values along the x-axis (**b**) and y-axis (**c**).

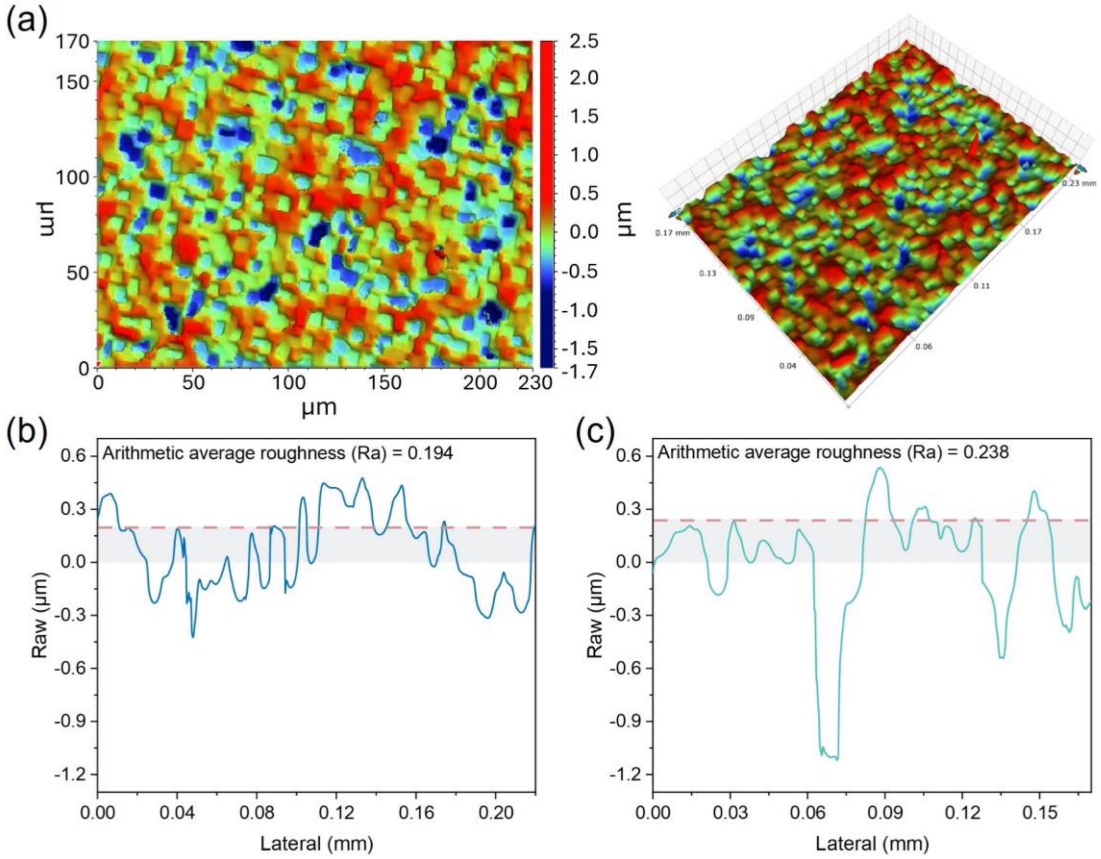


**Fig. S24. Optical surface profiler measurement of EM-2.** (**a**) Two-dimensional and three-dimensional reconstructions. Raw values along the x-axis (**b**) and y-axis (**c**).

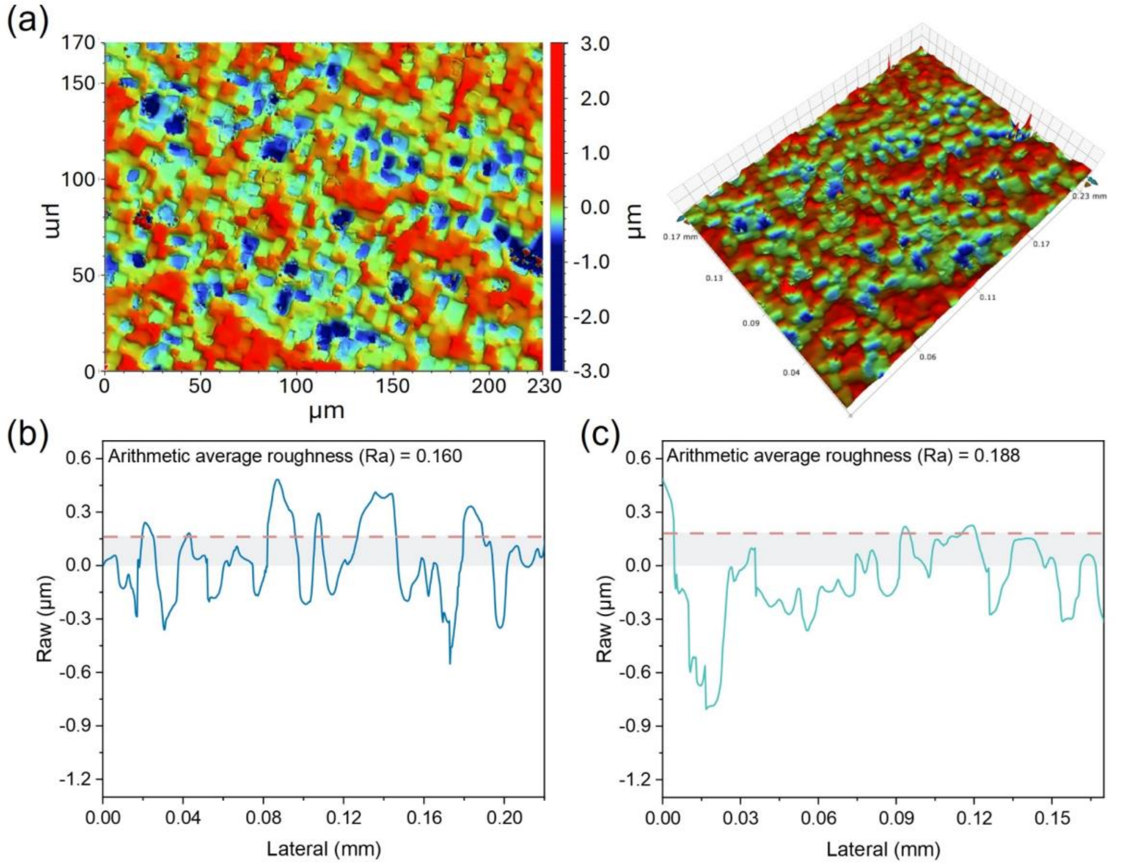


**Fig. S25. Optical surface profiler measurement of EM-3.** (**a**) Two-dimensional and three-dimensional reconstructions. Raw values along the x-axis (**b**) and y-axis (**c**).

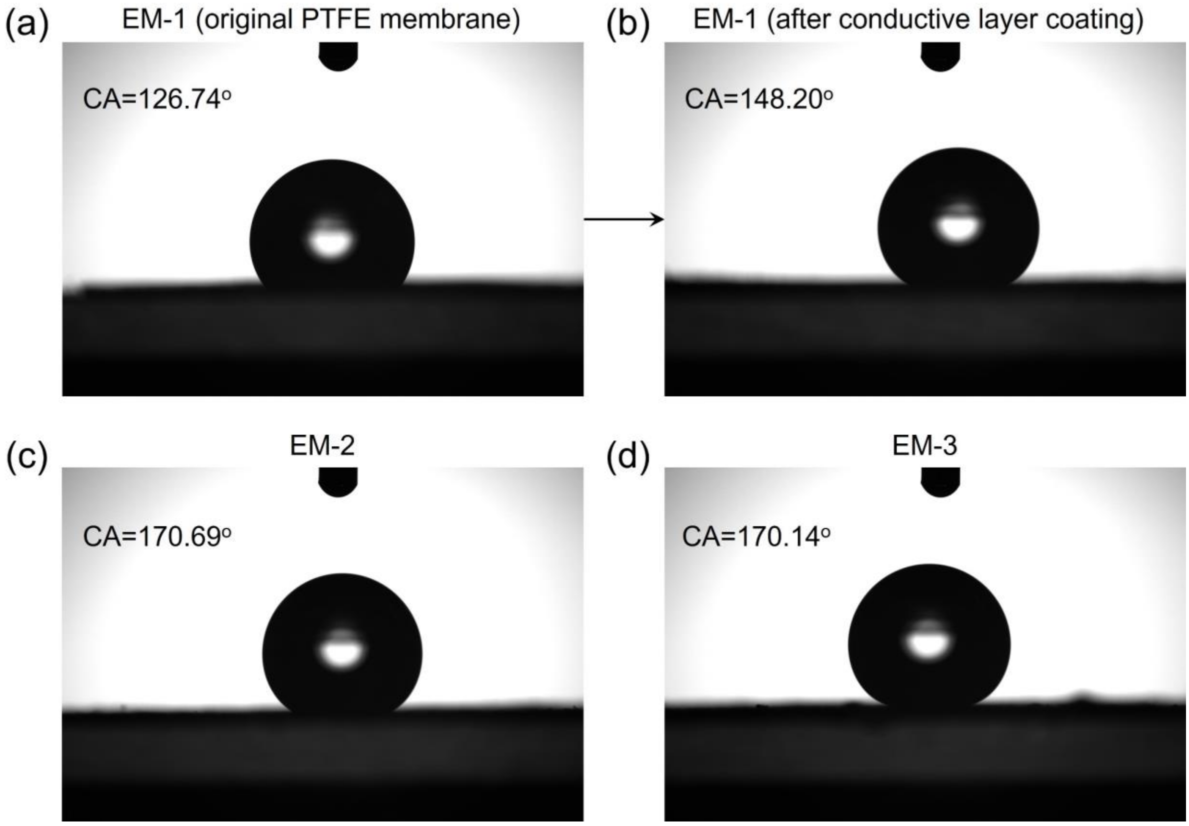


**Fig. S26. Water contact angle tests.** (**a** and **b**) EM-1, (**c**) EM-2, and (**d**) EM-3. The test electrolyte was simulated ammonium containing wastewater (0.25 M $(NH_4)_2SO_4$, 0.1 M $Na_2SO_4$). The drop value of electrolyte was 15 μL.

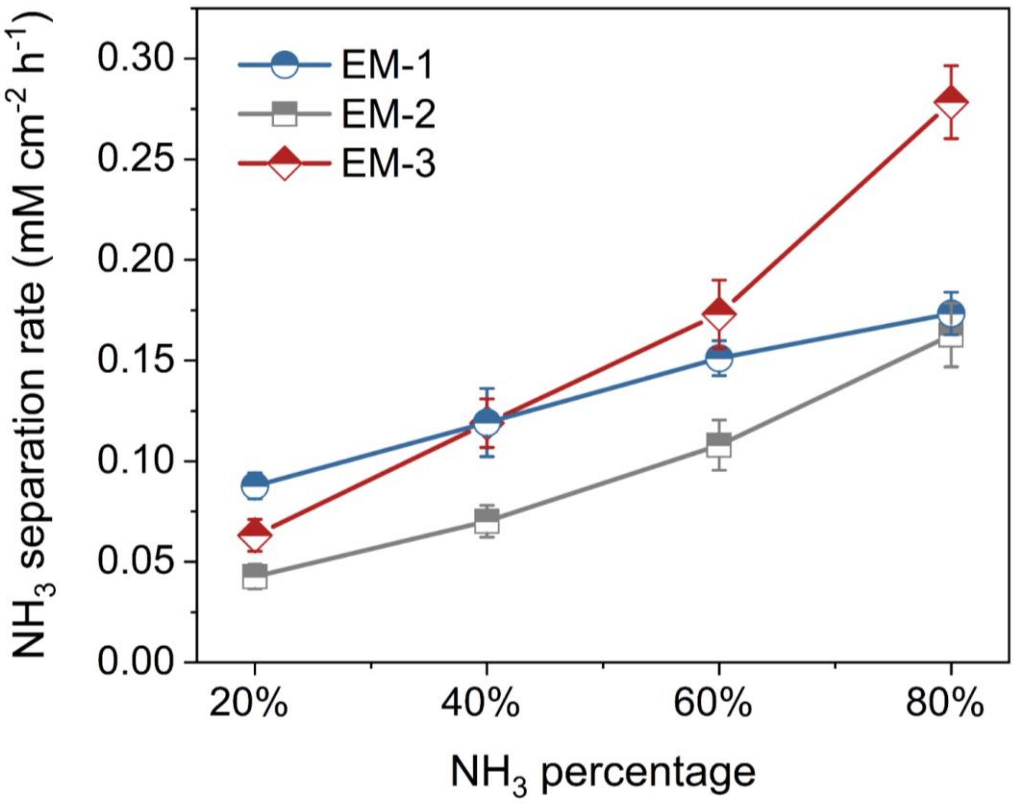


**Fig. S27. $NH_3$ separation rates of EM-1, EM-2, and EM-3 at different $NH_3$ percentages.** The $NH_3$ percentages were adjusted by varying the pH of simulated ammonium containing wastewater (0.25 M $NH_4^+$, 0.1 M $Na_2SO_4$). The electrolyte pH was adjusted with NaOH. The experimental data are presented as the mean ± standard deviation (SD) from at least three independent experiments.

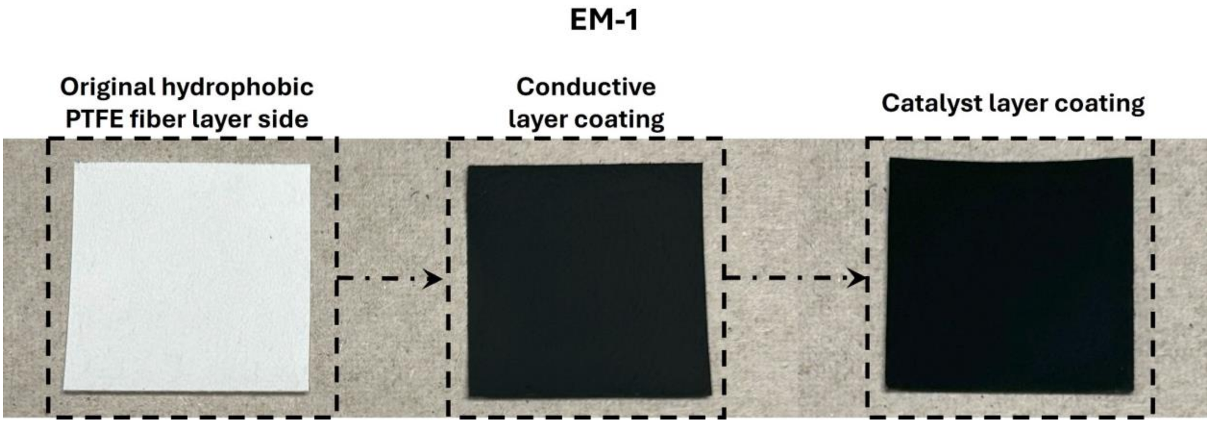


**Fig S28. Illustration of the construction of EM-1.** The hydrophobic PTFE fiber layer side of original PTFE membrane was first coated with a conductive layer consisting of carbon particles and CNTs, followed by coating of the catalyst layer.

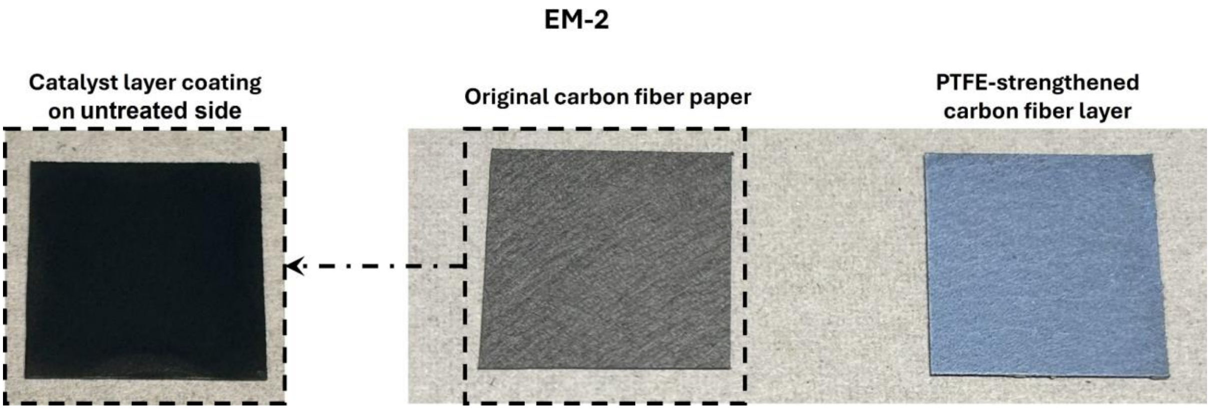


**Fig. S29. Illustration of the construction of EM-2.** One side of the carbon fiber paper was first treated by PTFE solution spray and calcination. Then, a catalyst layer was coated on the other untreated side.

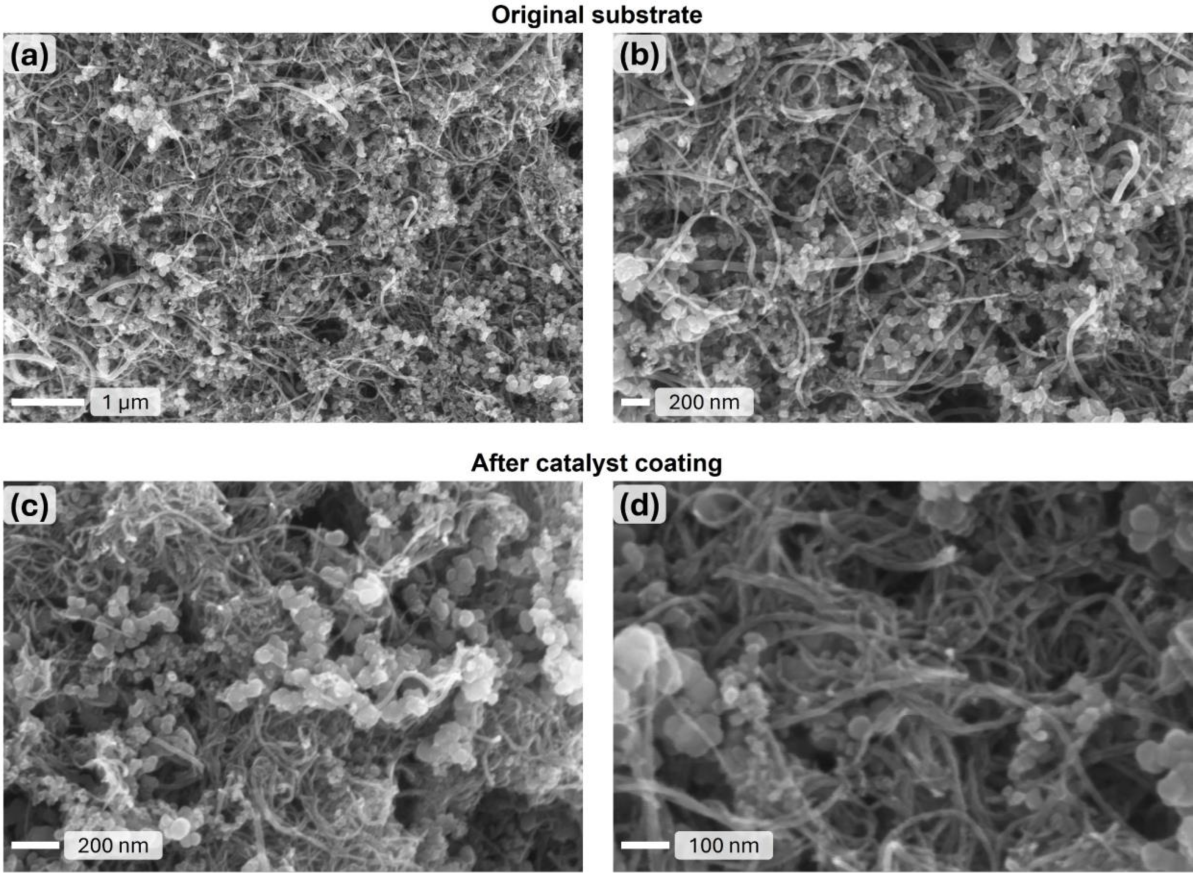


**Fig. S30. SEM images of the EM-1.** The original substrate, shown in (**a**) and (**b**), is a conductive layer consisting of carbon particles and CNTs on a PTFE membrane. The catalyst layer, shown in (**c**) and (**d**), consists of carbon particles and NiPc/CNT, with a NiPc/CNT loading density of 0.5 mg $cm^{-2}$.

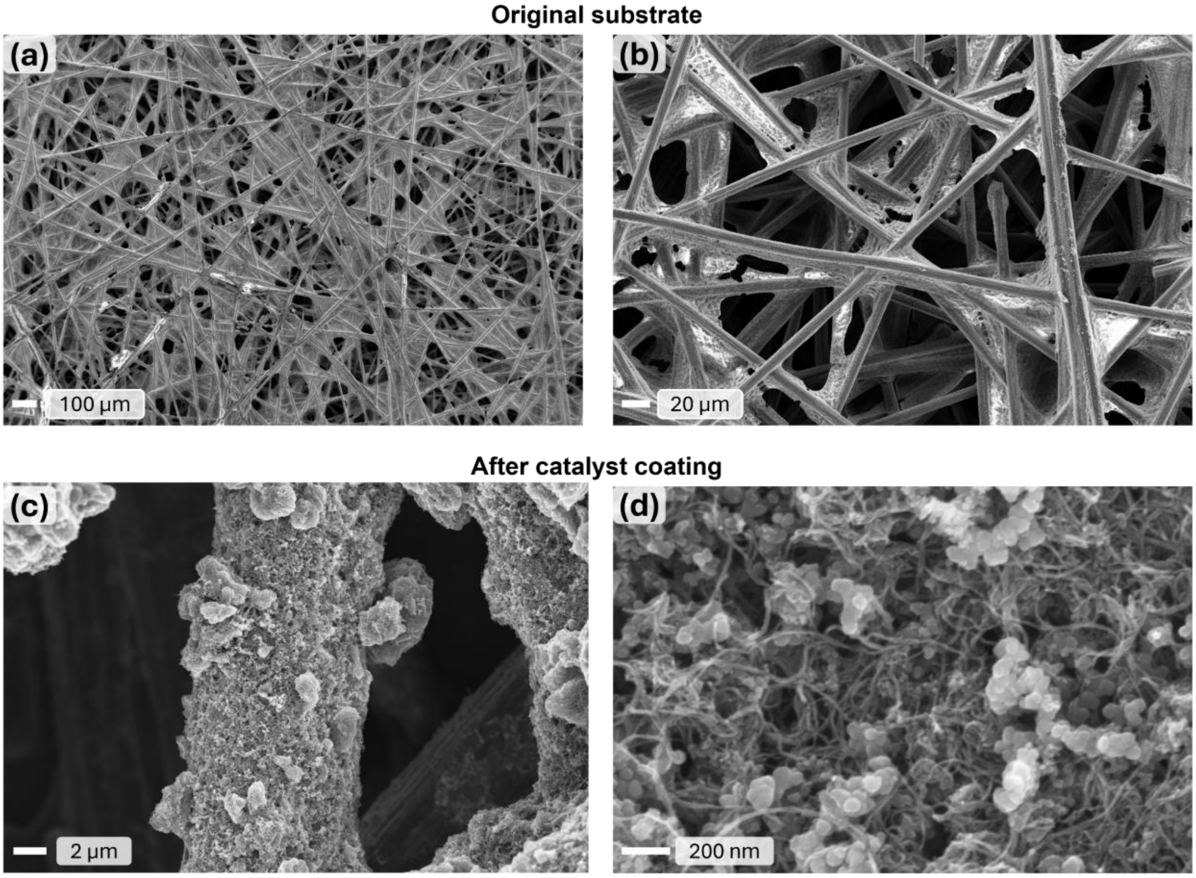


**Fig. S31. SEM images of the EM-2.** The original substrate, shown in (**a**) and (**b**), is a carbon fiber paper. The catalyst layer, shown in (**c**) and (**d**), consists of carbon particles and NiPc/CNT, with a NiPc/CNT loading density of 0.5 mg $cm^{-2}$.

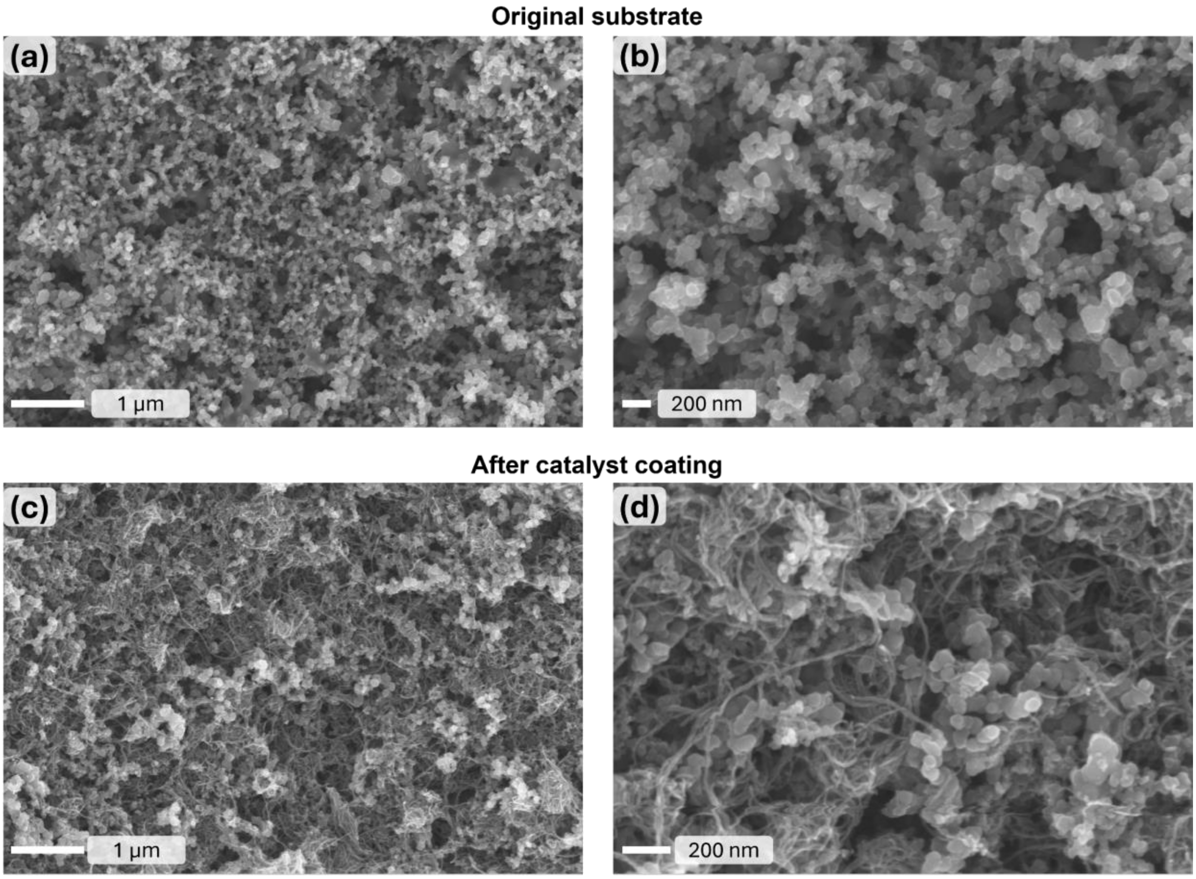


**Fig. S32. SEM images of the EM-3.** The original substrate, shown in (**a**) and (**b**), is a carbon fiber paper with a hydrophobic carbon microporous layer. The catalyst layer, shown in (**c**) and (**d**), consists of carbon particles and NiPc/CNT, with a NiPc/CNT loading density of 0.5 mg $cm^{-2}$.

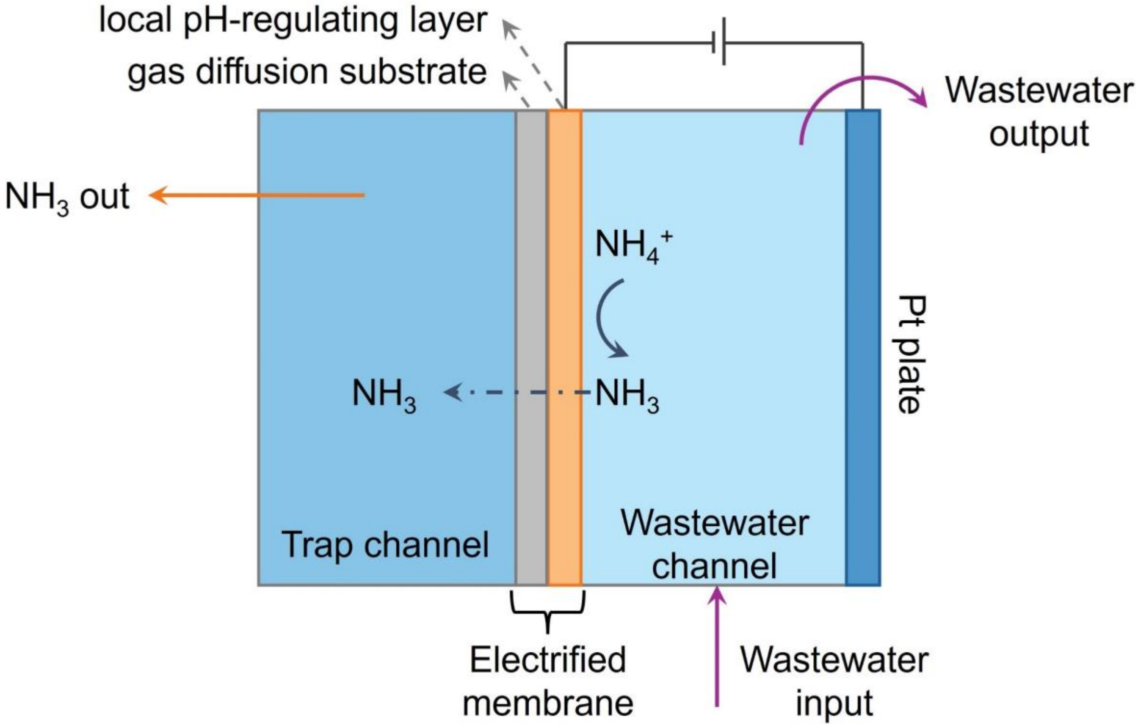


**Fig. S33. Illustration of flow-type electrolyzer for $NH_3$ separation.** Wastewater continuously flowed into and out of the wastewater chamber, while $NH_3$ separated from the wastewater was continuously transferred into the trap chamber.

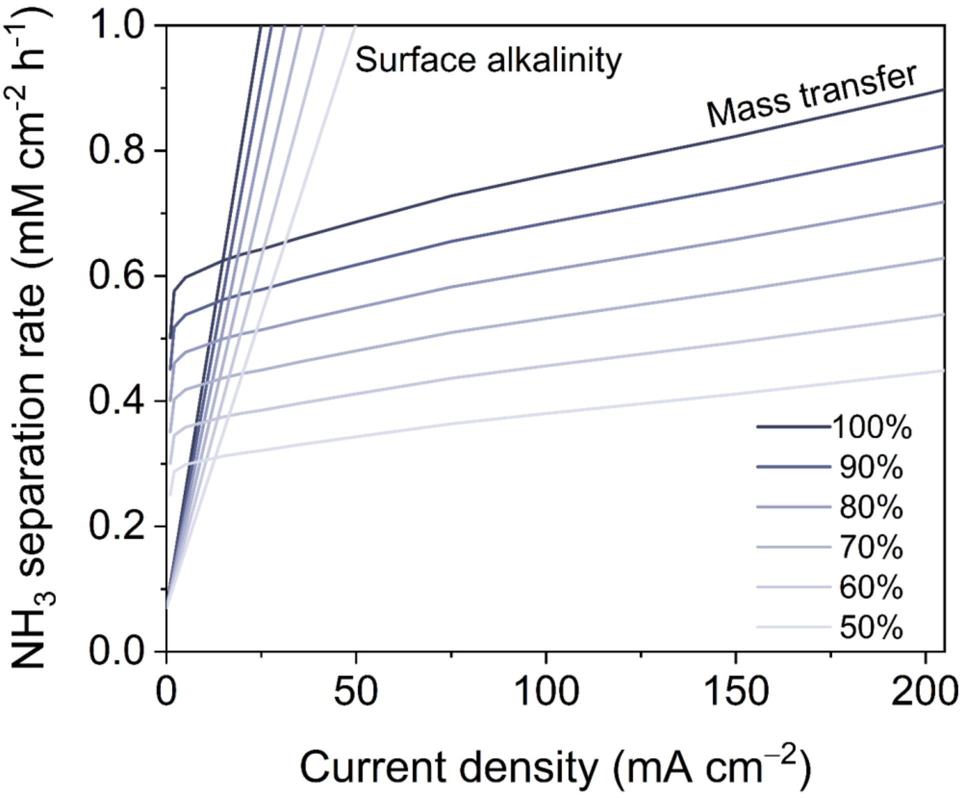


**Fig. S34. Theoretical model of $NH_3$ separation rate as a function of current density.** 100% towards surface alkalinity represents an ideal condition in which all locally generated interfacial alkalinity participates in $NH_4^+$-to-$NH_3$ conversion and all formed $NH_3$ is immediately separated from wastewater. 100% towards mass transfer represents the theoretical maximum $NH_4^+$ transferred to electrified membrane surface. Values from 90% to 50% indicate different degrees of interfacial alkalinity utilization efficiency or mass transfer efficiency.

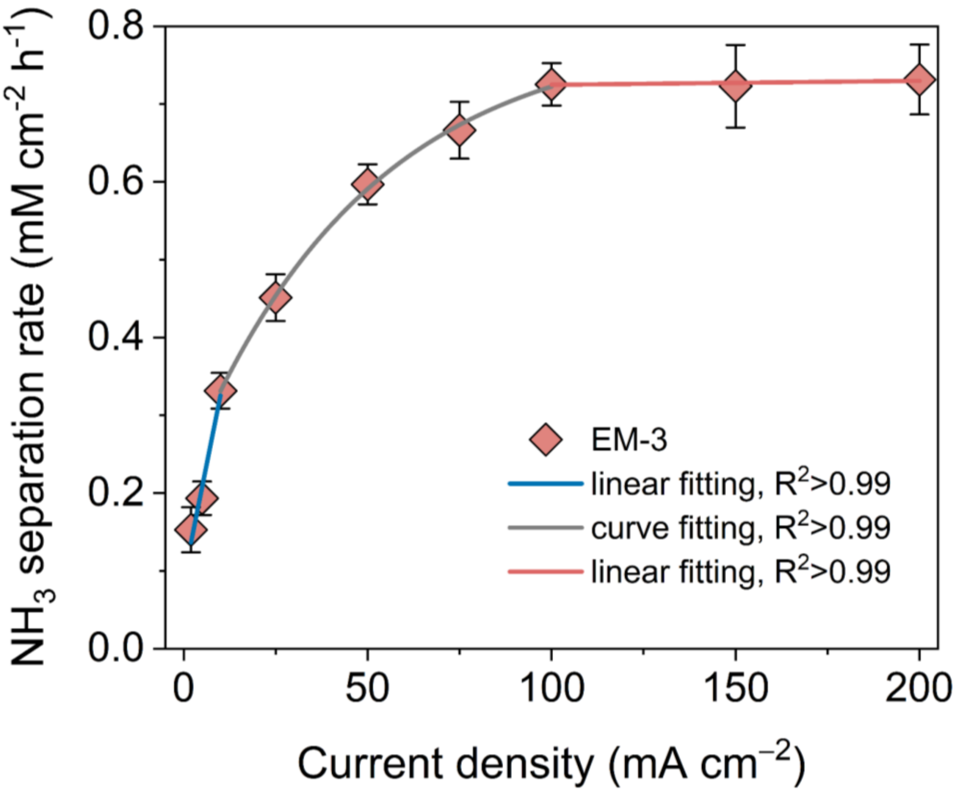


**Fig. S35. $NH_3$ separation rate of EM-3.** The total ammonium/ammonia concentration was 0.25 M. 0.1 M $Na_2SO_4$ aqueous electrolyte (pH=9.0) was used to simulate the ionic strength of environmentally relevant wastewater conditions. The experimental data are presented as the mean ± SD from at least three independent experiments.

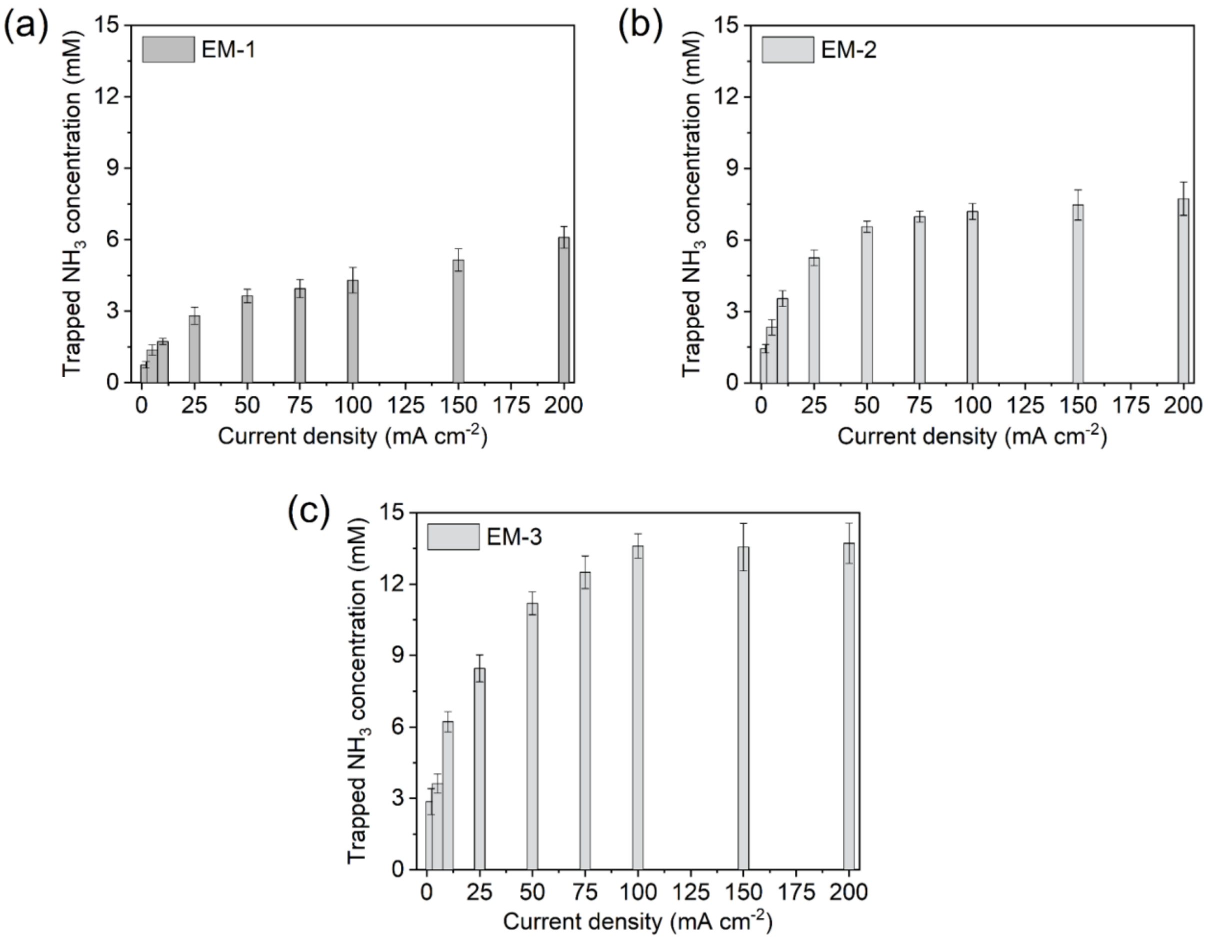


**Fig. S36. Comparison of EM1, EM2, and EM3.** Trapped $NH_3$ concentrations of EM-1 (**a**), EM-2 (**b**), EM-3 (**c**) under different applied current densities. The data correspond to **Fig. 3D** in the manuscript. The experimental data are presented as the mean ± SD from at least three independent experiments.

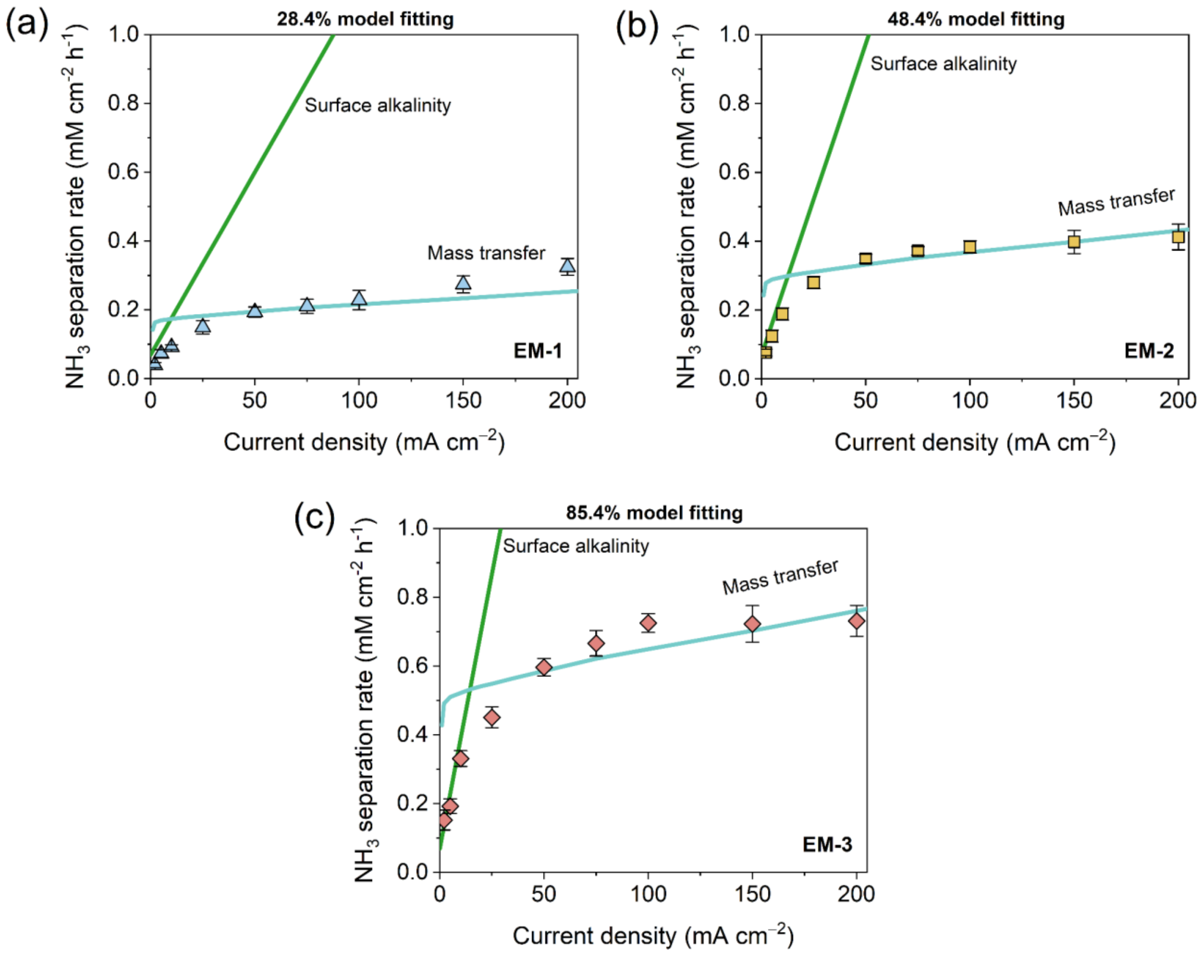


**Fig. S37. Measured $NH_3$ separation rates of different electrified membranes and corresponding theoretical model curves with different correction coefficients.** The experimental data are presented as the mean ± SD from at least three independent experiments. Correction coefficients of 28.4%, 48.4%, and 85.4% were applied to the predictive model of $NH_3$ separation flux to fit the experimentally measured $NH_3$ separation fluxes of EM-1, EM-2, and EM-3, respectively. Details of the coefficient calculations are provided in **Table S4**. These coefficients quantify the extent to which the measured $NH_3$ separation fluxes of EM-1, EM-2, and EM-3 approach the corresponding theoretical values.

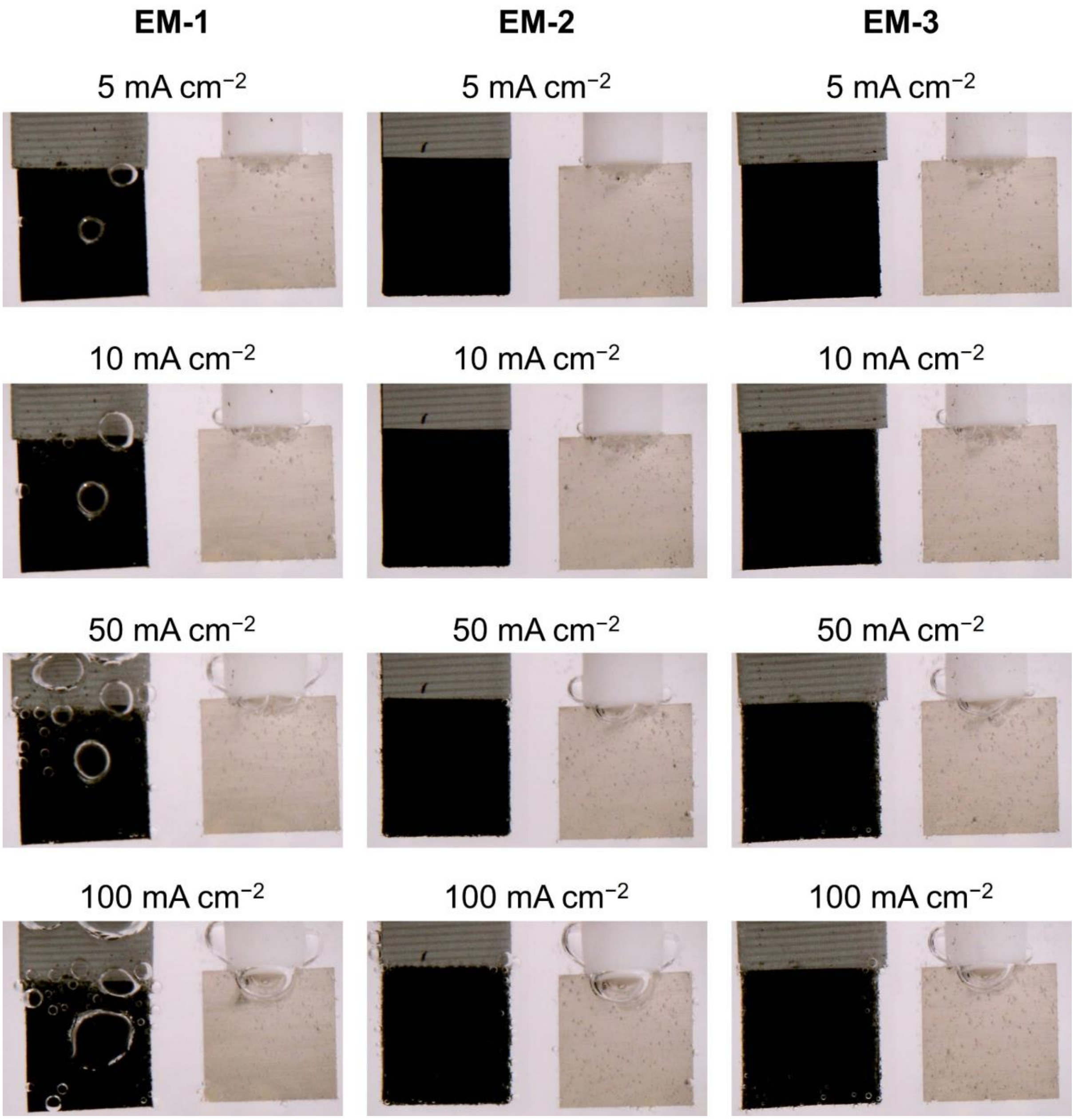


**Fig. S38. Photographs of the electrocatalyst layer side of electrified membranes under working conditions.** Different current densities were applied on the electrified membrane. In each photograph, the electrified membrane is located on the left, whereas the Pt plate serving as the counter electrode is located on the right.

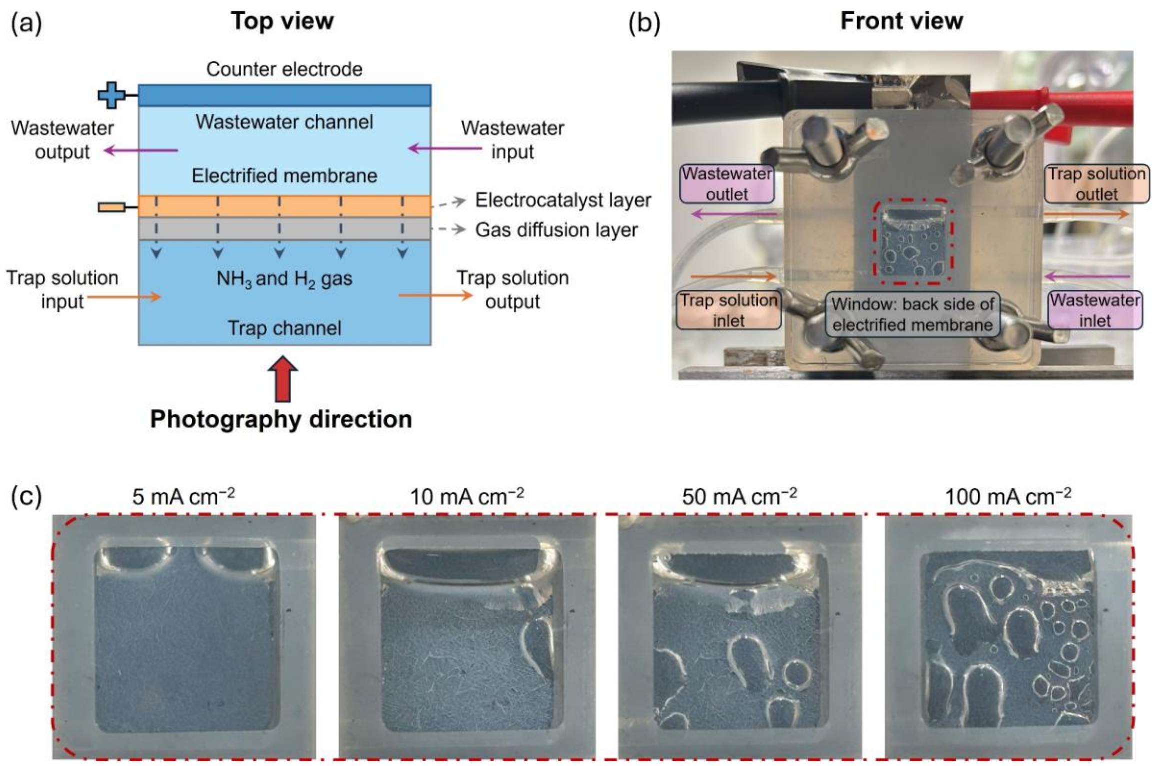


**Fig. S39. Illustration of gaseous products separation direction and real picture of electrified membrane under working conditions.** (**a**) The illustration of the configuration of flow-type electrolyzer and gaseous products separation direction. (**b**) The picture of electrified membrane under working conditions. (**c**) The picture of gas diffusion layer side of EM-2 under working conditions. Different current densities were applied on EM-2. The observed bubbles mainly correspond to separated $H_2$ gas, together with a small amount of undissolved $NH_3$ gas, from the wastewater.

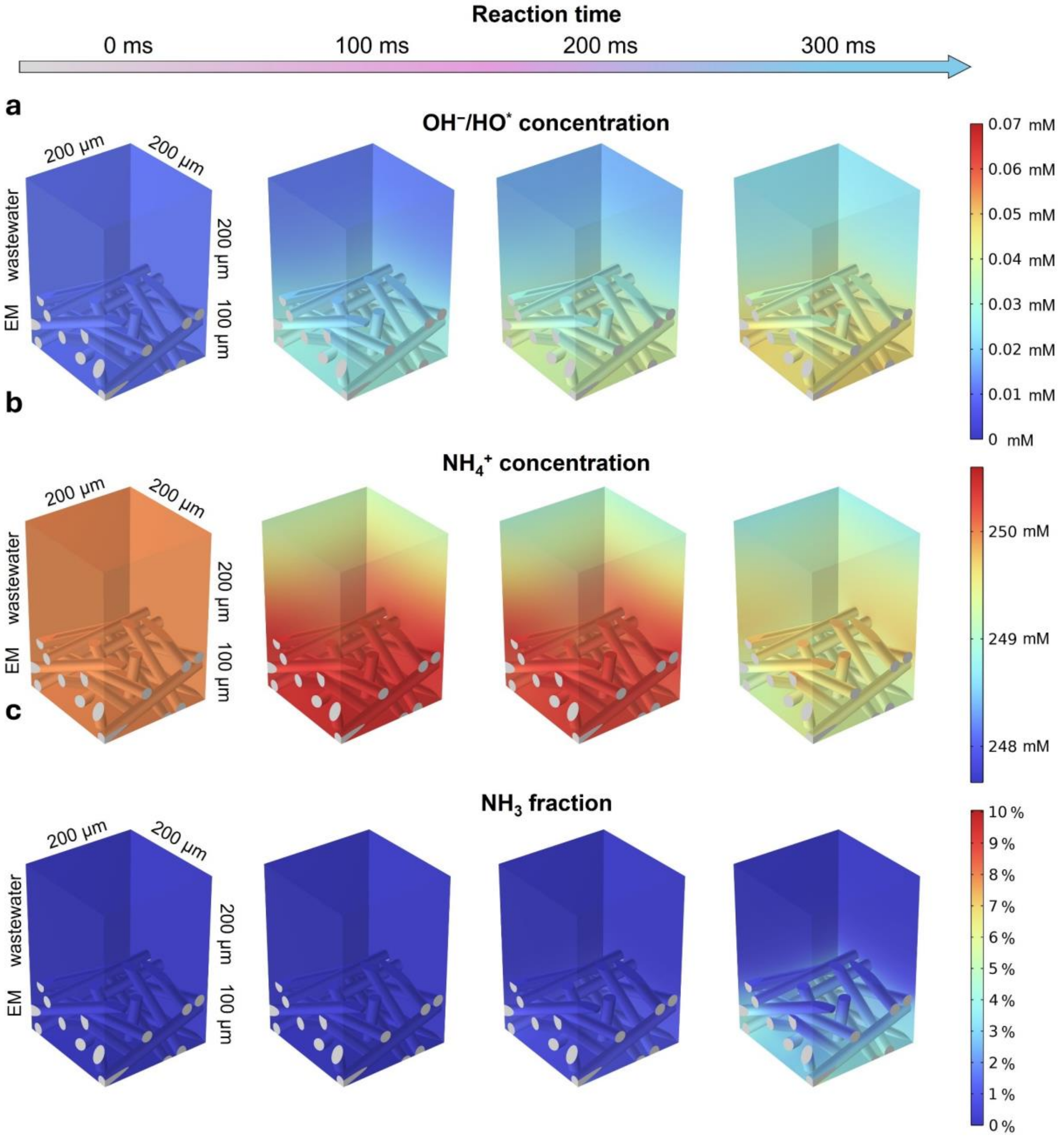


**Fig. S40. FEM simulations of EM-2.** (**a**) $OH^-/HO^*$ concentration, (**b**) $NH_4^+$ concentration, (**c**) $NH_3$ fraction at different distances from the membrane surface and reaction times (current density: 10 mA cm$^{-2}$, an electrified membrane area of 200 μm × 200 μm was employed for applied current density calculation). The initial $NH_4^+$ concentration in the simulated ammoniacal wastewater was 0.25 M.

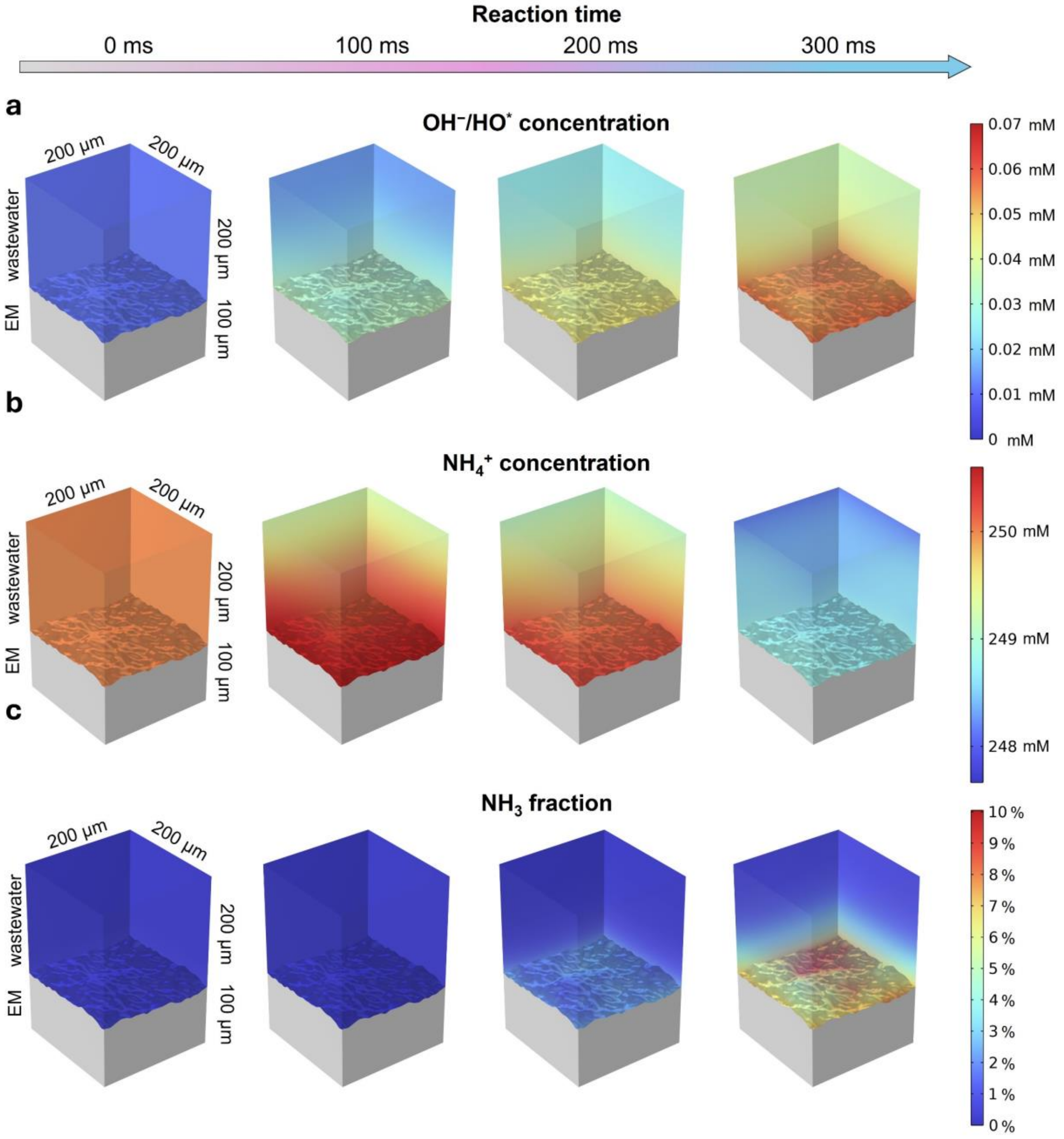


**Fig. S41. FEM simulations of EM-3.** (**a**) $OH^{-}/HO^{*}$ concentration, (**b**) $NH_4^+$ concentration, (**c**) $NH_3$ fraction at different distances from the membrane surface and reaction times (current density: 10 mA cm$^{-2}$, an electrified membrane area of 200 μm × 200 μm was employed for applied current density calculation). The initial $NH_4^+$ concentration in the simulated ammoniacal wastewater was 0.25 M.

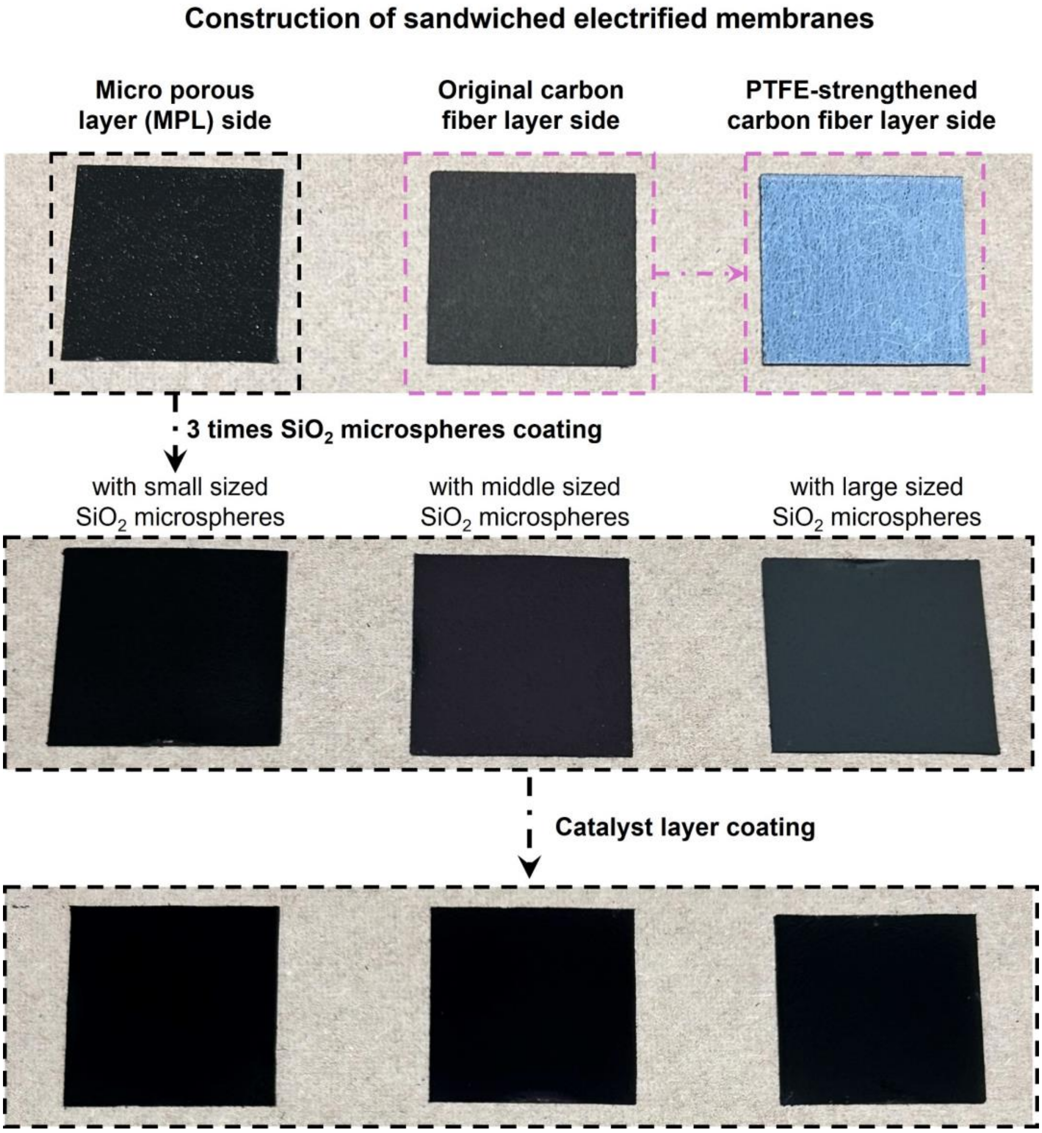


**Fig. S42. Illustration of the construction of sandwiched electrified membranes.** First, the carbon fiber paper side was hydrophobically treated (PTFE solution spray followed by calcination operation, detailed in Materials and Methods Section). Then, gas adsorption layers with $SiO_2$ microspheres of different sizes were loaded on micro porous layer side. Catalyst layer was a mixture of carbon particles and NiPc/CNT, with a NiPc/CNT loading density of 0.5 mg $cm^{-2}$.

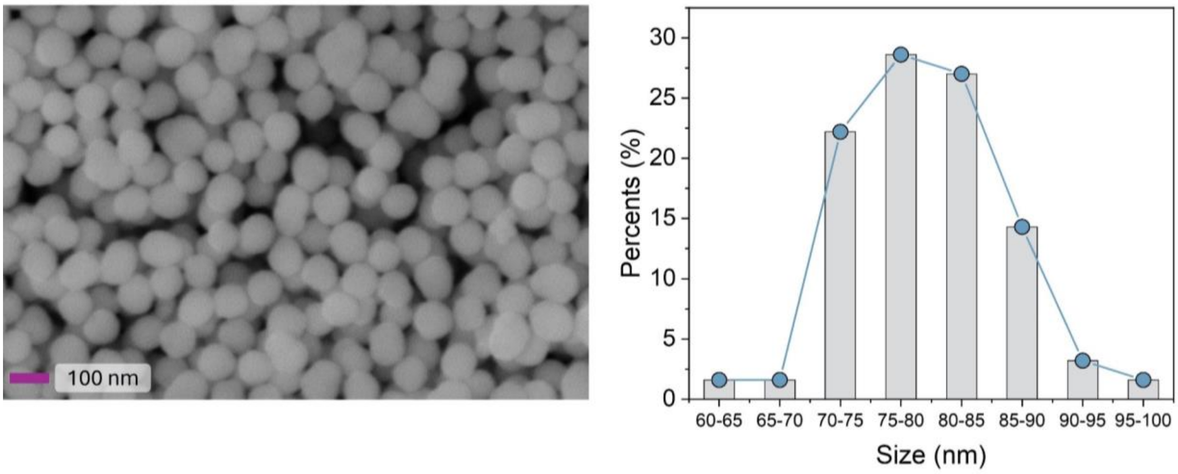


**Fig. S43. Size quantification of small sized $SiO_2$ microspheres.** SEM image (left) and particle size distribution (right) of small sized $SiO_2$ microspheres. The mean diameter (around 0.08±0.01 μm) was estimated based on the particles shown in the SEM image.

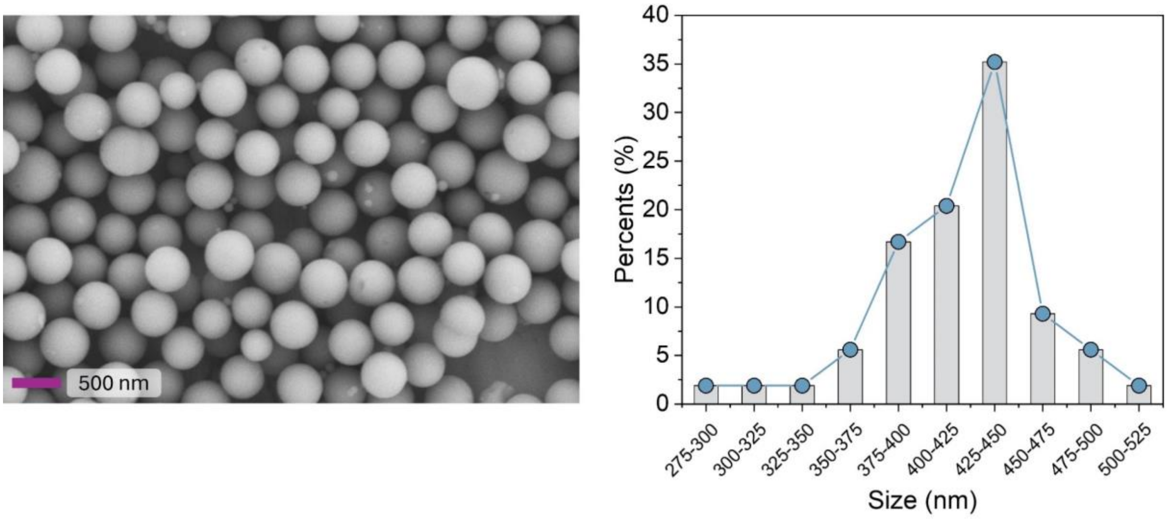


**Fig. S44. Size quantification of middle sized $SiO_2$ microspheres.** SEM image (left) and particle size distribution (right) of middle sized $SiO_2$ microspheres. The mean diameter (around 0.42±0.05 μm) was estimated based on the particles shown in the SEM image.

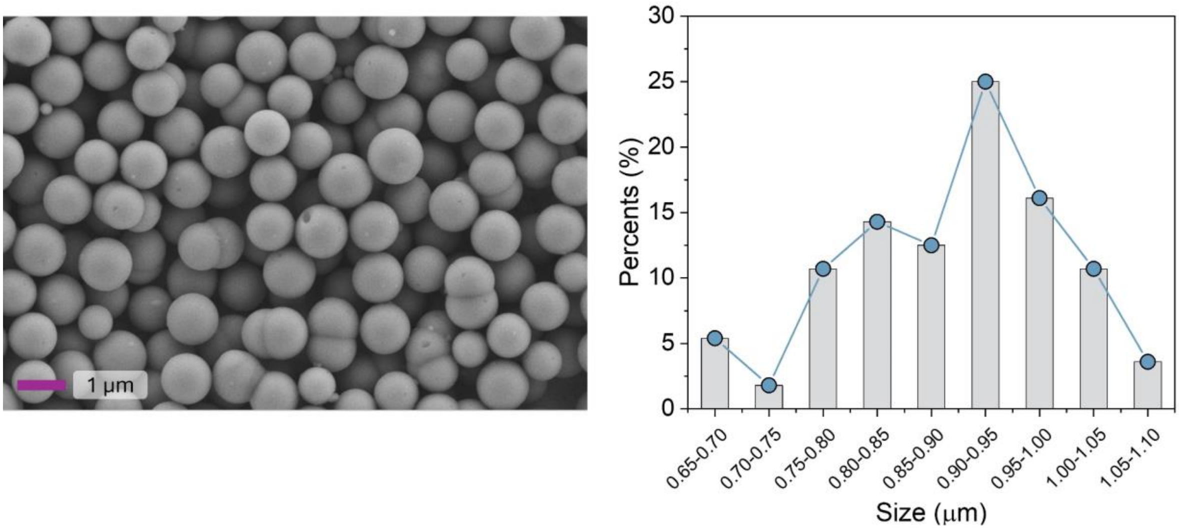


**Fig. S45. Size quantification of large sized $SiO_2$ microspheres.** SEM image (left) and particle size distribution (right) of large sized $SiO_2$ microspheres. The mean diameter (around 0.90±0.07 μm) was estimated based on the particles shown in the SEM image.

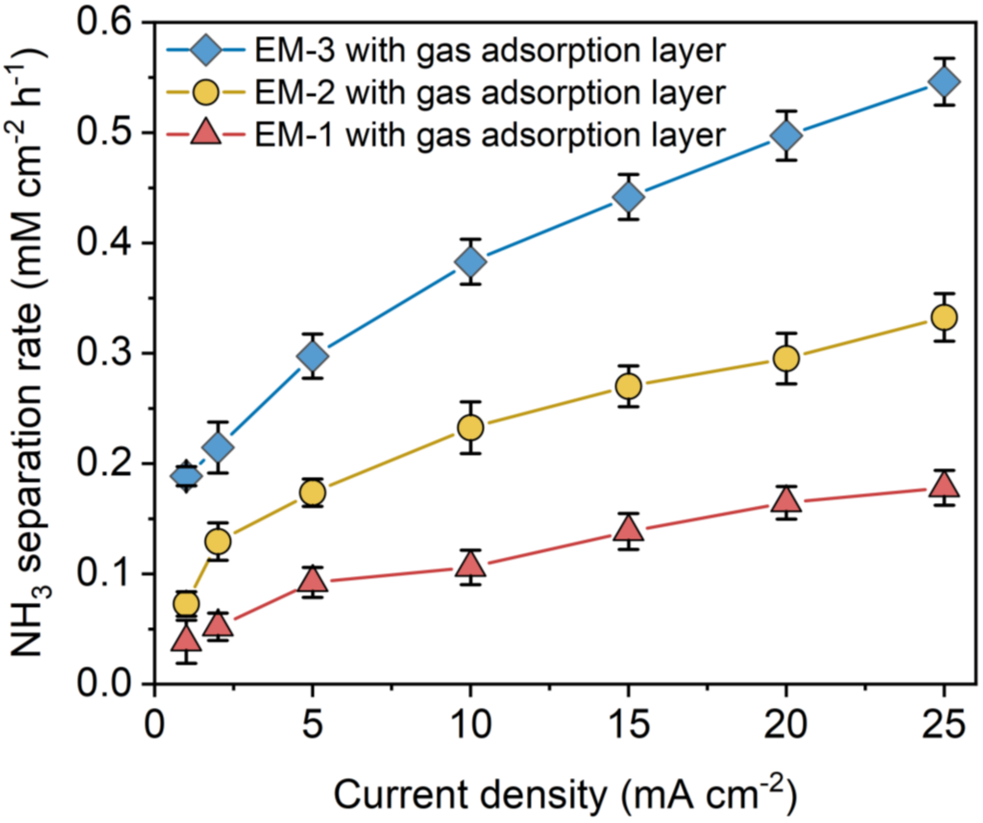


**Fig. S46. Comparison of the $NH_3$ separation rate of EM-1, EM-2, and EM-3 with gas adsorption layer.** The gas adsorption layer consists of $SiO_2$ microspheres, carbon particles, and CNTs conductive network. The loading density of middle sized $SiO_2$ microspheres (particle size: 0.42±0.05 μm) was 5 mg cm$^{-2}$. The experimental data are presented as the mean ± SD from at least three independent experiments.

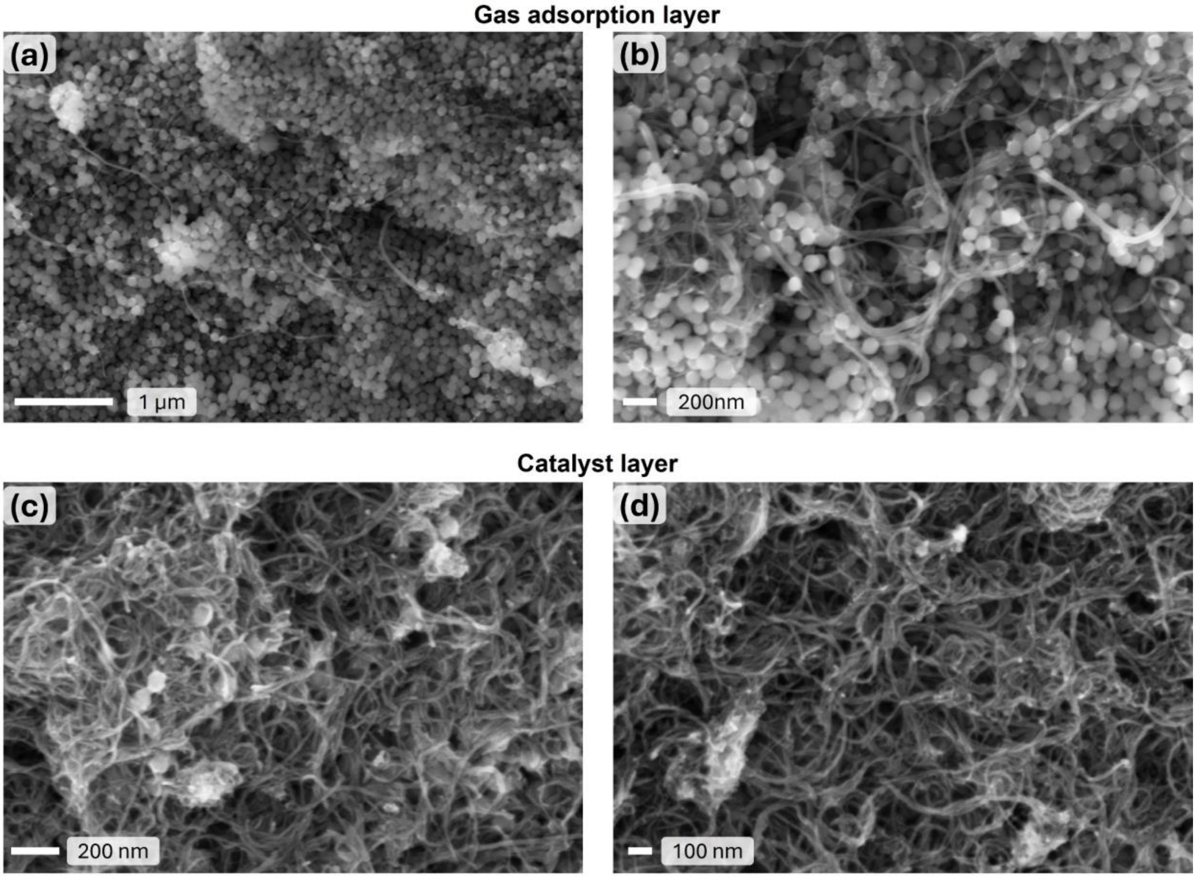


**Fig. S47. SEM images of EM-3 with a gas adsorption layer containing small sized $SiO_2$ microspheres.** The morphology of gas adsorption layer is shown in (**a**) and (**b**). The gas adsorption layer consists of small sized $SiO_2$ microspheres (particle size: 0.08±0.01 μm), carbon particles, and CNTs conductive network. The loading density of $SiO_2$ microspheres was 5 mg $cm^{-2}$. The morphology of catalyst layer is shown in (**c**) and (**d**). The catalyst layer consists of carbon particles and NiPc/CNT, with a NiPc/CNT loading density of 0.5 mg $cm^{-2}$.

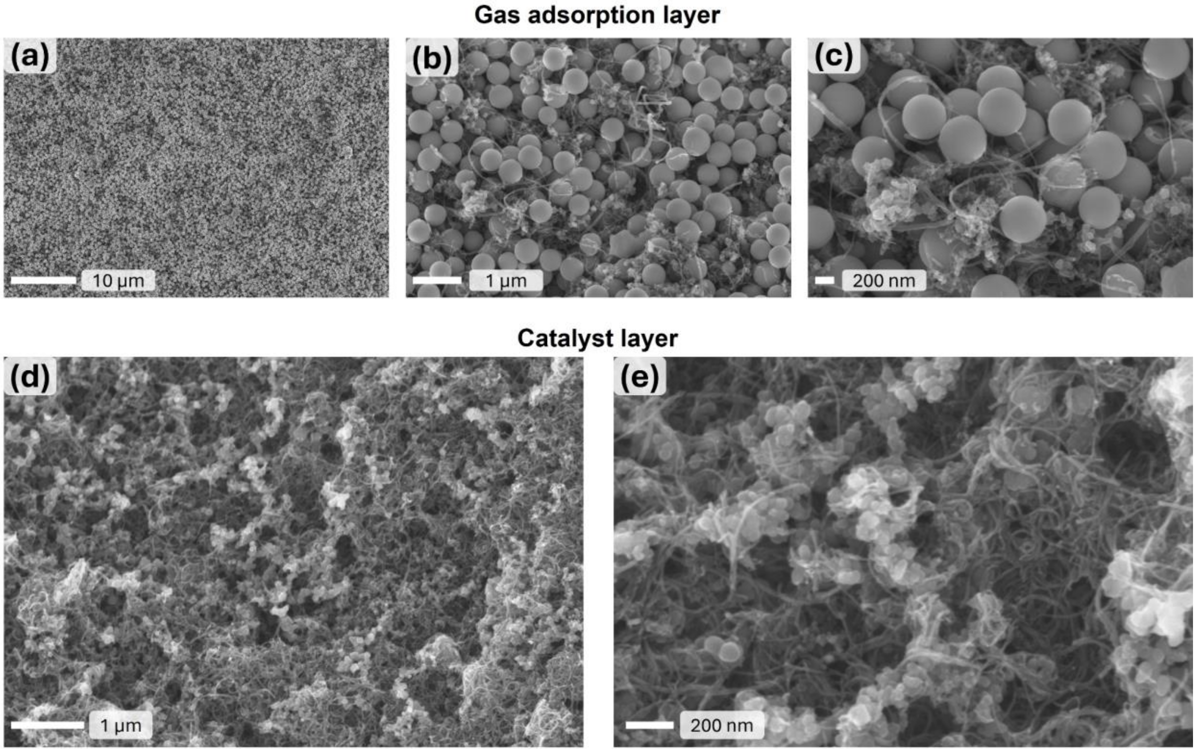


**Fig. S48. SEM images of EM-3 with a gas adsorption layer containing middle sized $SiO_2$ microspheres.** The morphology of gas adsorption layer is shown in (**a**)-(**c**). The gas adsorption layer consists of middle sized $SiO_2$ microspheres (particle size: 0.42±0.05 μm), carbon particles, and CNTs conductive network. The loading density of $SiO_2$ microspheres was 5 mg $cm^{-2}$. The morphology of catalyst layer is shown in (**d**) and (**e**). The catalyst layer consists of carbon particles and NiPc/CNT, with a NiPc/CNT loading density of 0.5 mg $cm^{-2}$.

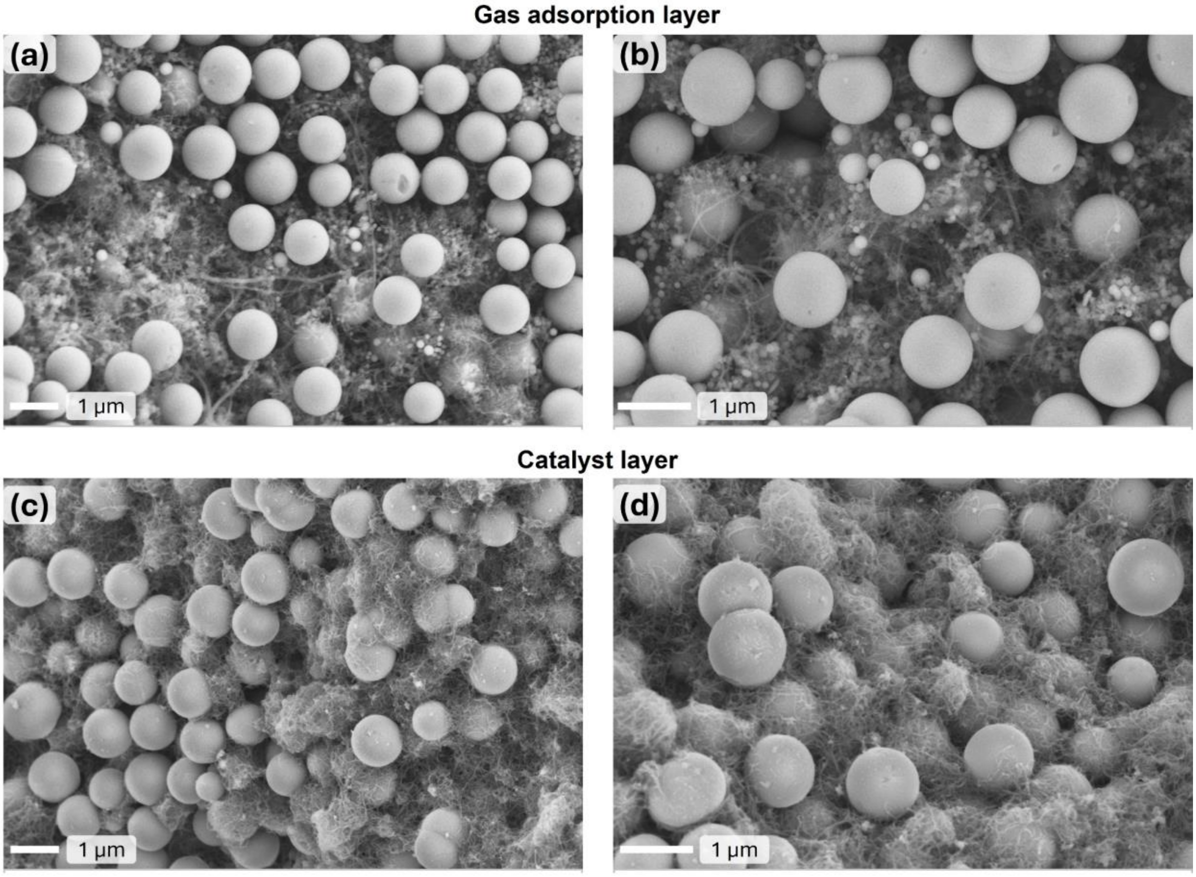


**Fig. S49. SEM images of EM-3 with a gas adsorption layer containing large sized $SiO_2$ microspheres.** The morphology of gas adsorption layer is shown in (**a**) and (**b**). The gas adsorption layer consists of large sized $SiO_2$ microspheres (particle size: 0.90±0.07 μm), carbon particles, and CNTs conductive network. The loading density of $SiO_2$ microspheres was 5 mg $cm^{-2}$. The morphology of catalyst layer is shown in (**c**) and (**d**). The catalyst layer consists of carbon particles and NiPc/CNT, with a NiPc/CNT loading density of 0.5 mg $cm^{-2}$.

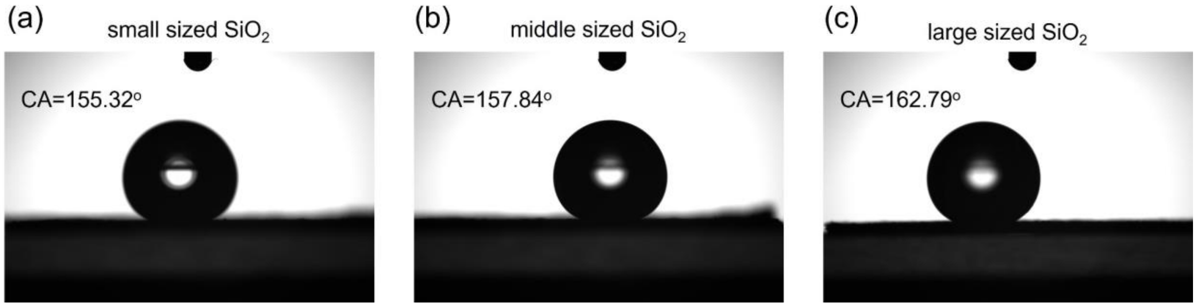


**Fig. S50. Effect of $SiO_2$ particle size on wettability.** EM-3 covered with small (**a**), middle (**b**), and large (**c**) sized $SiO_2$ microspheres. The loading density of $SiO_2$ microspheres in each case was 5 mg $cm^{-2}$. The test electrolyte was the simulated ammonium containing wastewater (0.25 M $(NH_4)_2SO_4$, 0.1 M $Na_2SO_4$). The drop value of electrolyte was 15 μL.

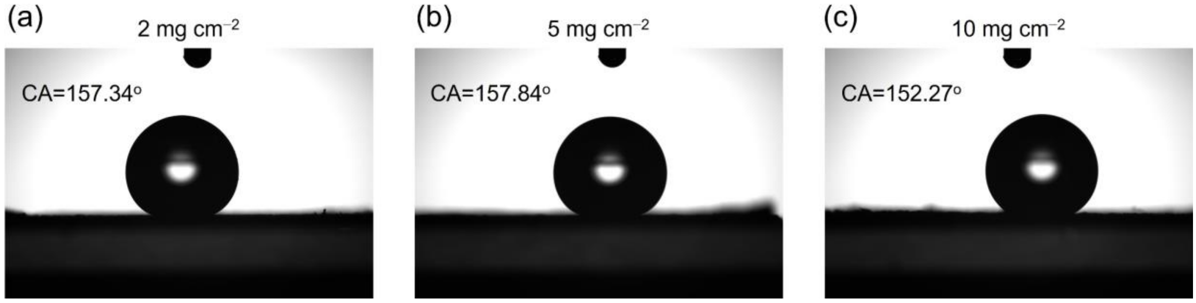


**Fig. S51. Effect of $SiO_2$ loading density on wettability.** EM-3 covered with 2 mg cm$^{-2}$ (**a**), 5 mg cm$^{-2}$ (**b**), and 10 mg cm$^{-2}$ (**c**) middle sized $SiO_2$ microspheres (particle size: 0.42±0.05 μm). The test electrolyte was the simulated ammonium containing wastewater (0.25 M $(NH_4)_2SO_4$, 0.1 M $Na_2SO_4$). The drop value of electrolyte was 15 μL. Fig. S51 (b) is the same as Fig. S50 (b) because it belong to middle sized $SiO_2$ particles covering EM-3 with a loading mass of 5 mg cm$^{-2}$.

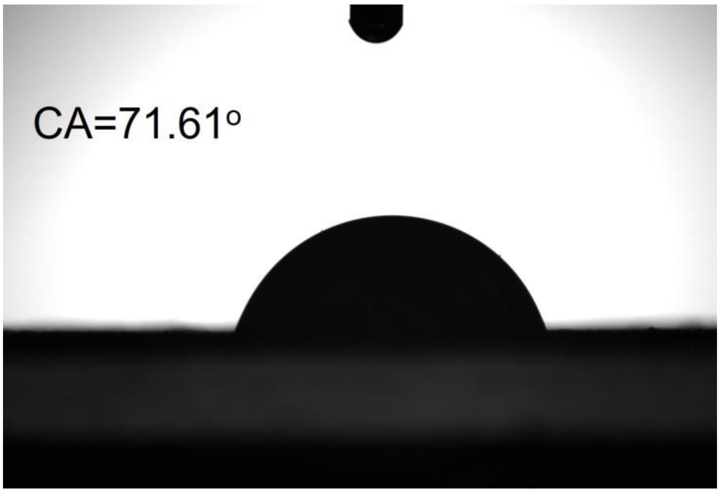


**Fig. S52. Effect of $SiO_2$ hydrophobicity on wettability.** The EM-3 was covered with middle sized $SiO_2$ microspheres (particle size: 0.42±0.05 μm) without hydrophobic treatment. The loading density of $SiO_2$ microspheres was 5 mg $cm^{-2}$. The test electrolyte was the simulated ammonium containing wastewater (0.25 M $(NH_4)_2SO_4$, 0.1 M $Na_2SO_4$). The drop value of electrolyte was 15 μL.

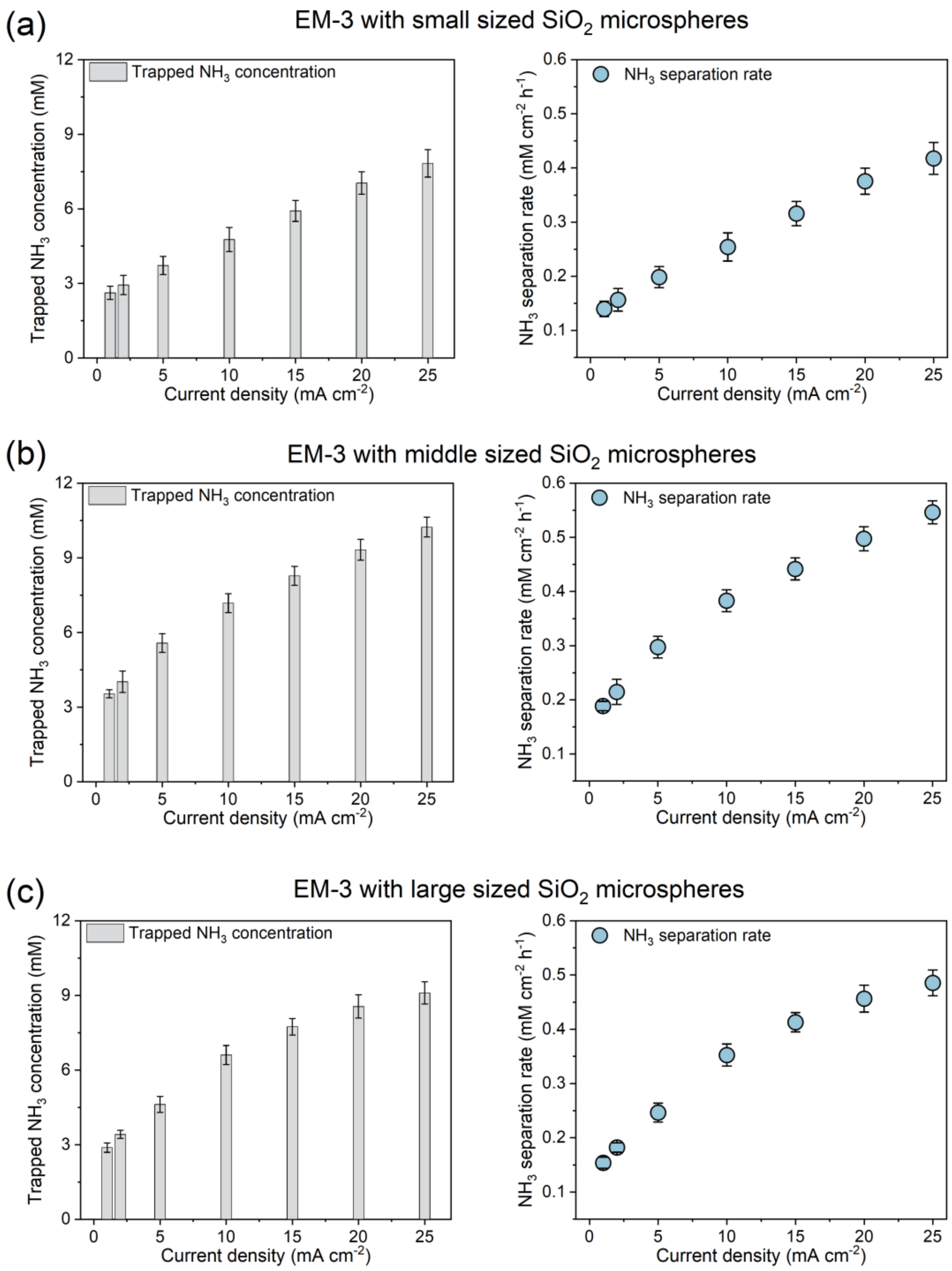


**Fig. S53. Optimization of $SiO_2$ particle size.** The concentrations of trapped $NH_3$ and $NH_3$ separation rate of gas adsorption layer on EM-3 with small (**a**), middle (**b**), and large (**c**) sized $SiO_2$ microspheres under different applied current densities. In each case, the loading densities of $SiO_2$ microspheres was 5 mg cm$^{-2}$. The experimental data are presented as the mean ± SD from at least three independent experiments.

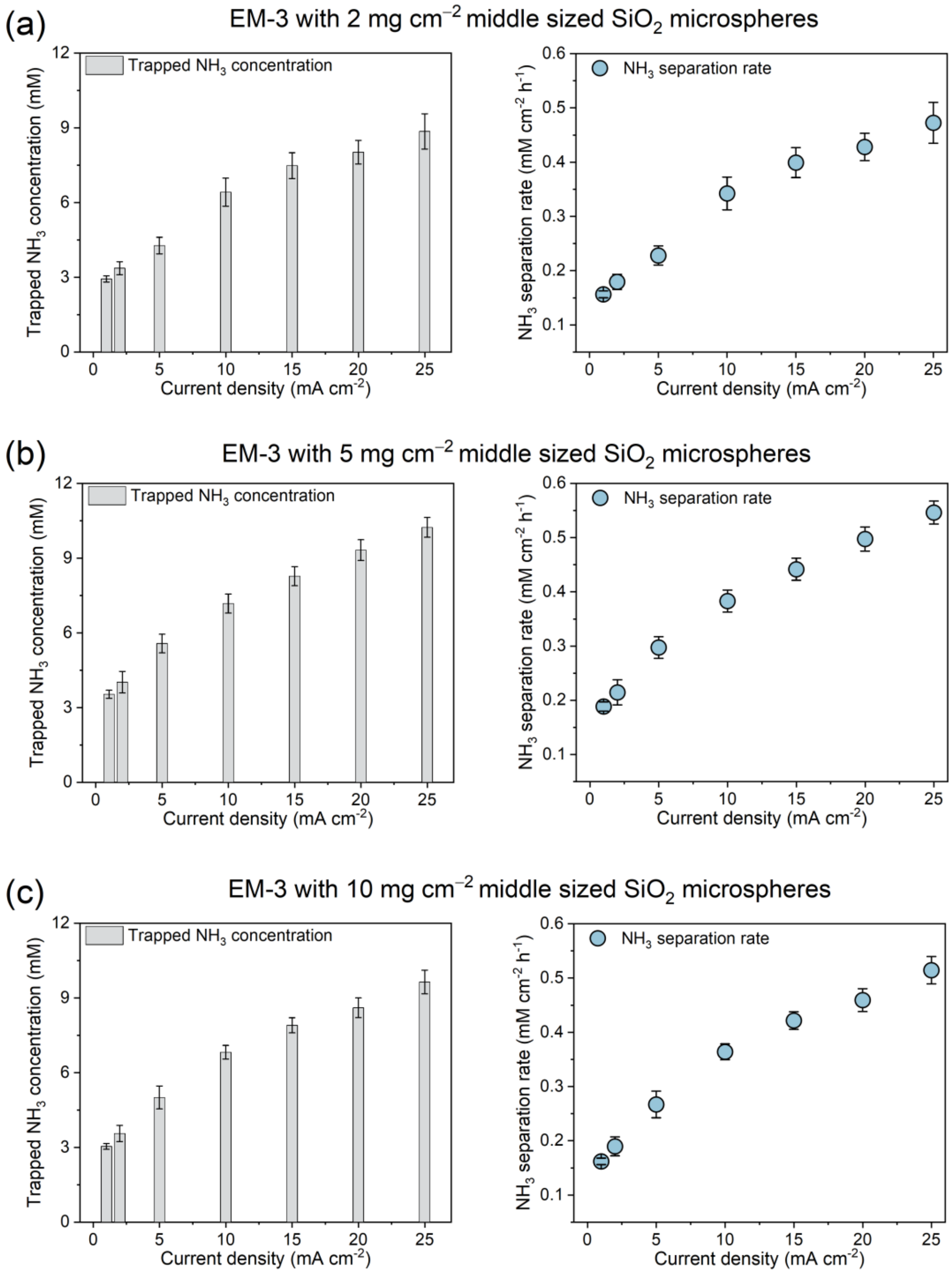


**Fig. S54. Optimization of $SiO_2$ loading density.** The concentrations of trapped $NH_3$ and $NH_3$ separation rate of gas adsorption layer on EM-3 with 2 mg cm$^{-2}$ (**a**), 5 mg cm$^{-2}$ (**b**), and 10 mg cm$^{-2}$ (**c**) $SiO_2$ microspheres under different applied current densities. The experimental data are presented as the mean ± SD from at least three independent experiments.

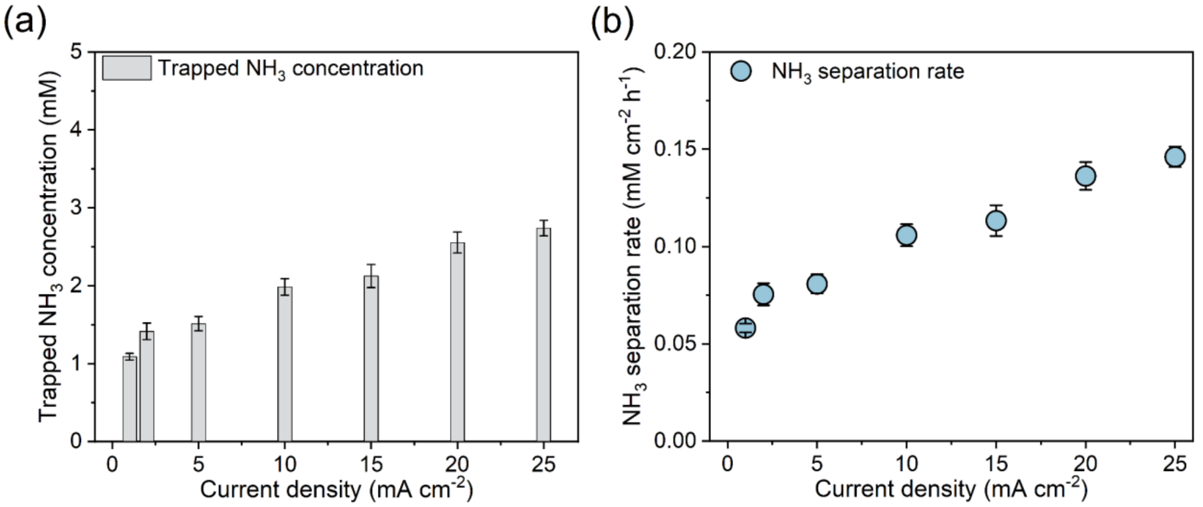


**Fig. S55. Effect of $SiO_2$ hydrophobicity on $NH_3$ separation.** The trapped $NH_3$ concentrations (**a**) and $NH_3$ separation rates (**b**) of gas adsorption layer (containing middle sized $SiO_2$ microspheres without hydrophobic treatment) on EM-3 under different applied current densities. The loading density of $SiO_2$ microspheres was 5 mg $cm^{-2}$. The experimental data are presented as the mean ± SD from at least three independent experiments.

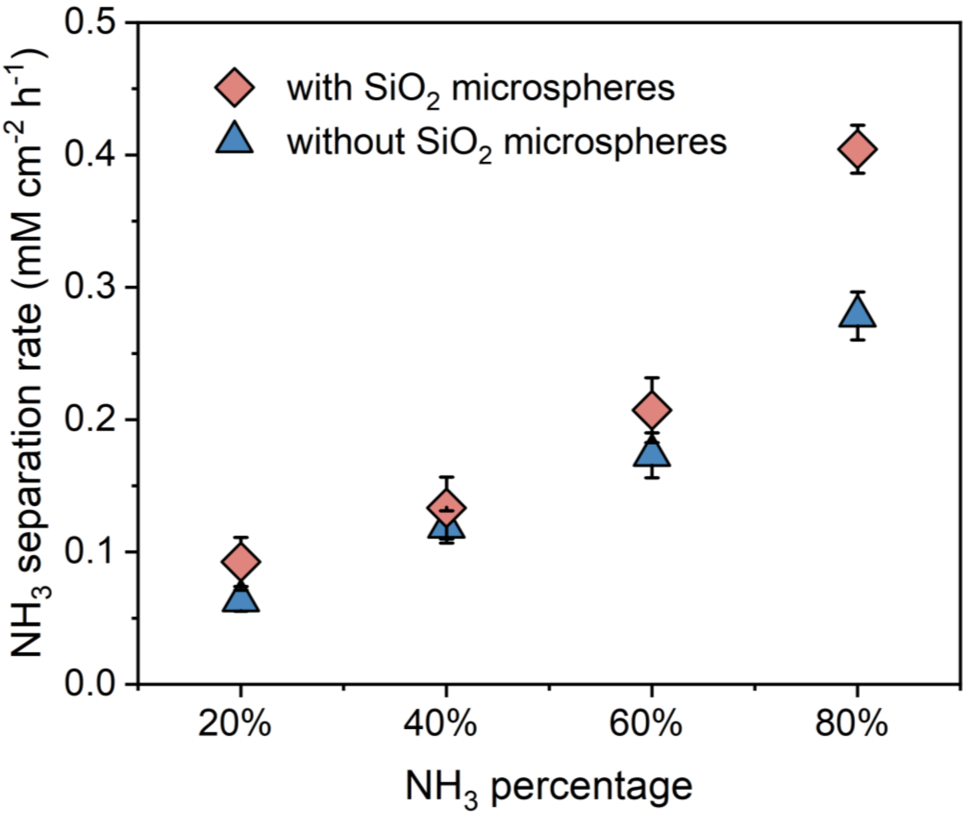


**Fig. S56. Effect of $SiO_2$ microspheres on $NH_3$ separation.** The $NH_3$ transfer rate of EM-3 with/without middle sized $SiO_2$ microspheres (without catalyst loading) vs. gaseous $NH_3$ percentage. The total ammonium/ammonia concentration was 0.25 M. 0.1 M $Na_2SO_4$ aqueous electrolyte was used to simulate the ionic strength of environmentally relevant wastewater conditions. The $NH_3$ percentage was controlled by adjusting the pH of simulated wastewater. The experimental data are presented as the mean ± SD from at least three independent experiments.

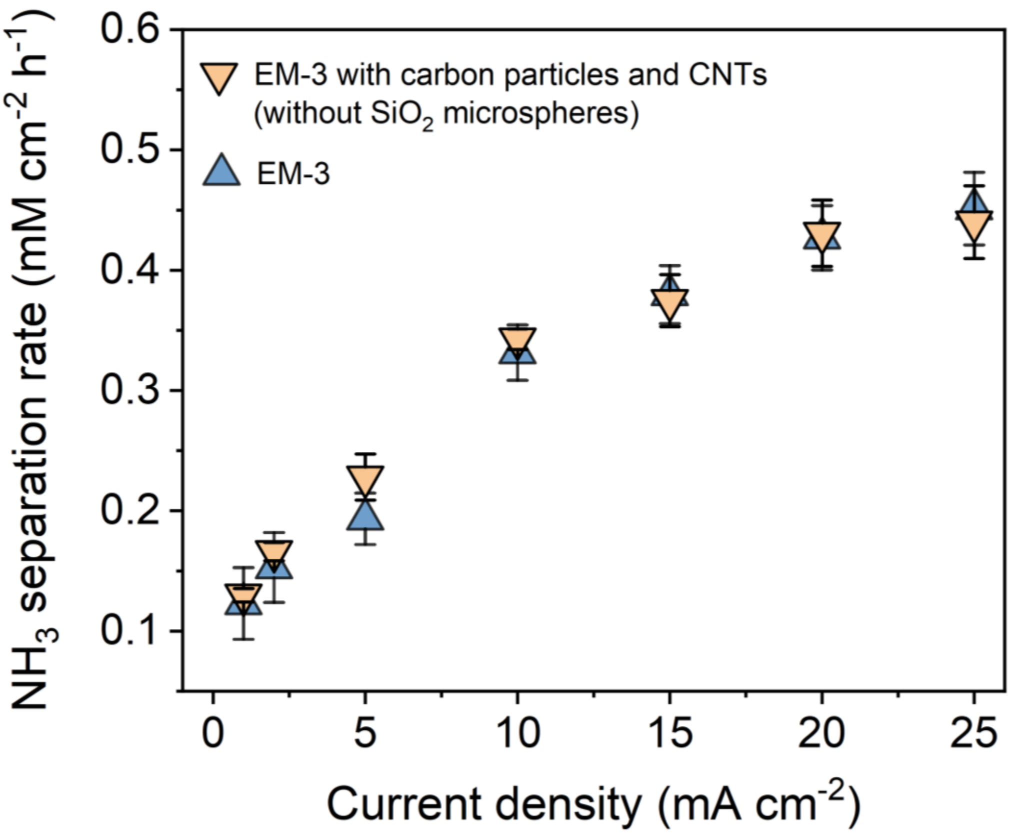


**Fig S57. Effect of carbon particles and CNTs on $NH_3$ separation.** The $NH_3$ separation rate of EM-3 with/without carbon particles and CNTs. The total ammonium/ammonia concentration was 0.25 M. 0.1 M $Na_2SO_4$ aqueous electrolyte (pH=9.0) was used to simulate the ionic strength of environmentally relevant wastewater conditions. The experimental data are presented as the mean ± SD from at least three independent experiments.

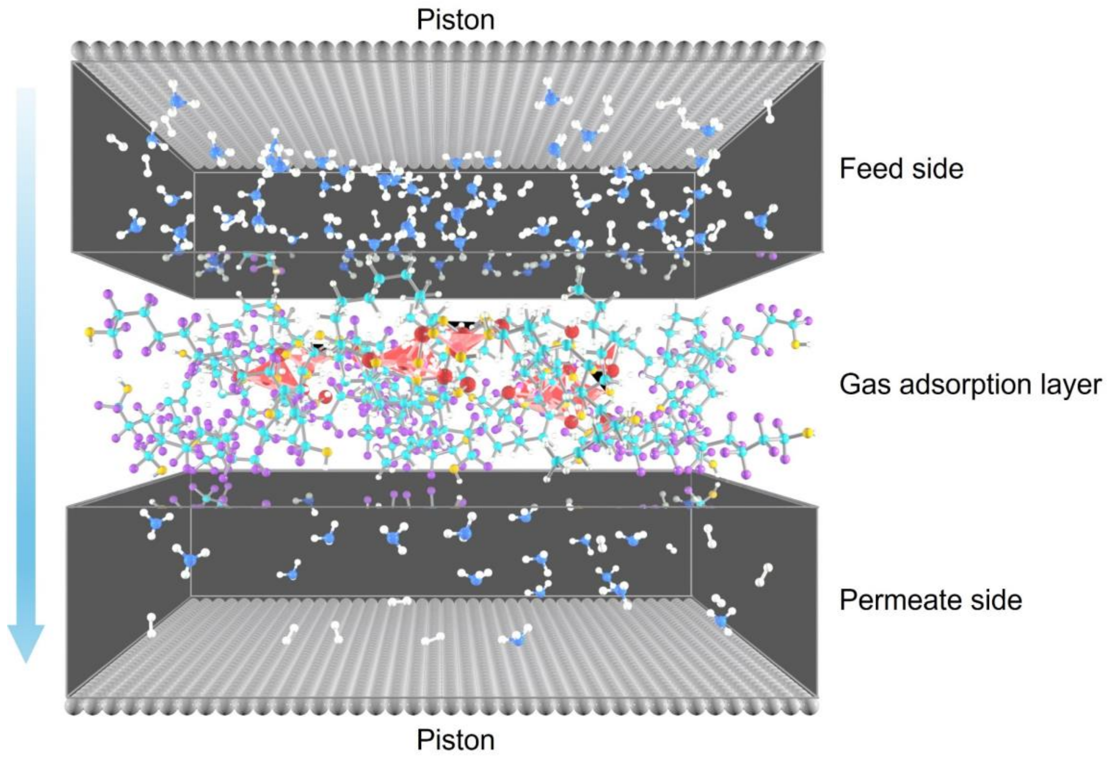


**Fig. S58. MD simulation model.** Illustration of MD simulation of $NH_3$ and $H_2$ gas molecules crossing gas adsorption layer.

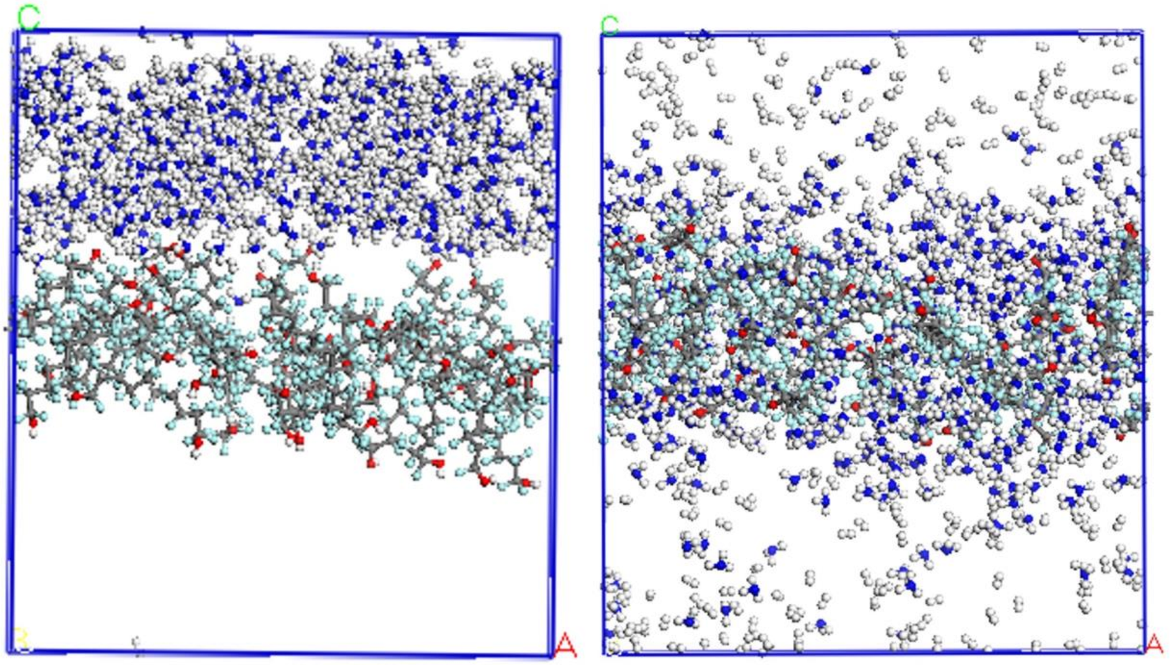

**Fig. S59. MD simulation of $NH_3$ and $H_2$ gas molecules crossing PTFE molecules.** The figures show the initial state (left) and finial state (right) of simulation.

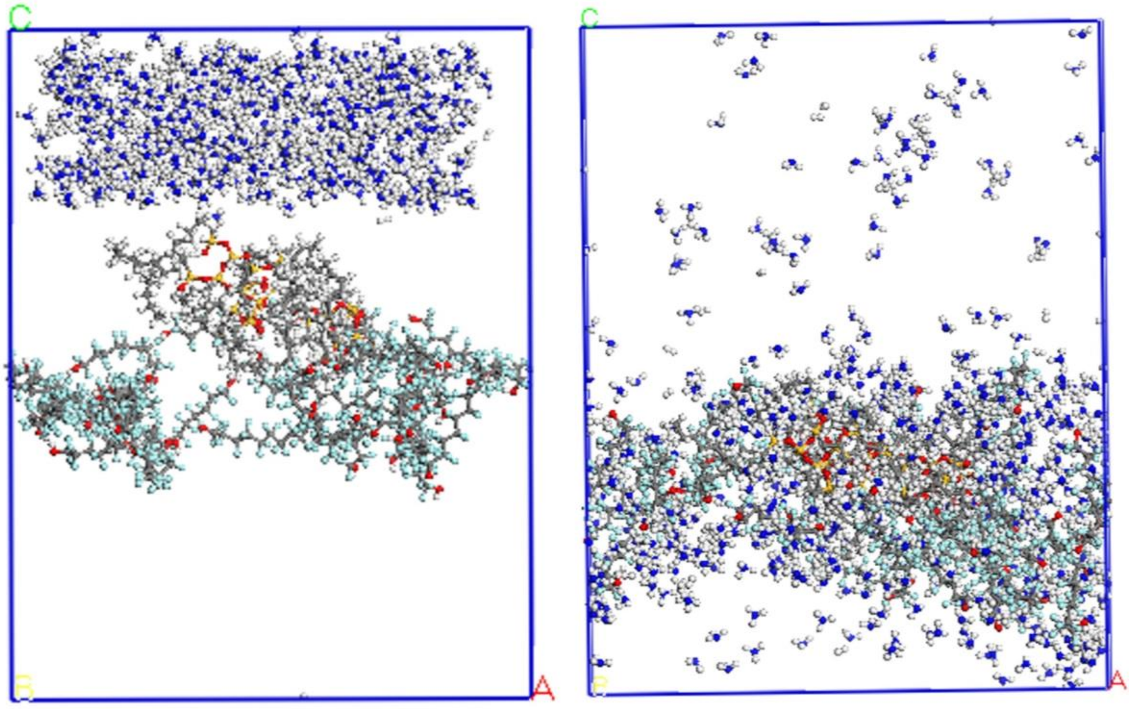


**Fig. S60. MD simulation of $NH_3$ and $H_2$ gas molecules crossing a mixture of $SiO_2$ and PTFE molecules.** The figures show the initial state (left) and final state (right) of simulation.

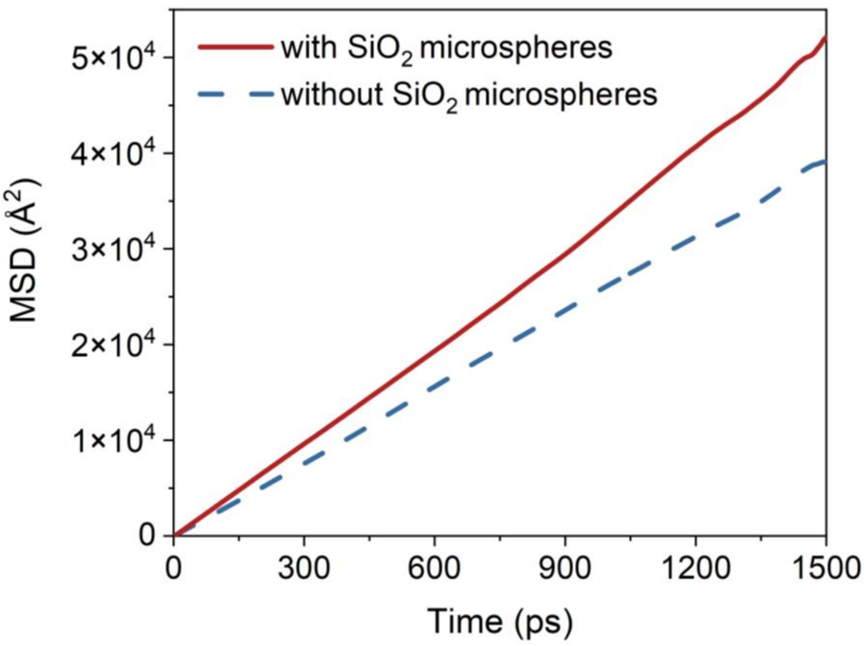


**Fig. S61. $NH_3$ diffusion in gas adsorption layers.** Mean square displacement (MSD) of $NH_3$ gas molecules crossing gas adsorption layers with/without $SiO_2$ microspheres.

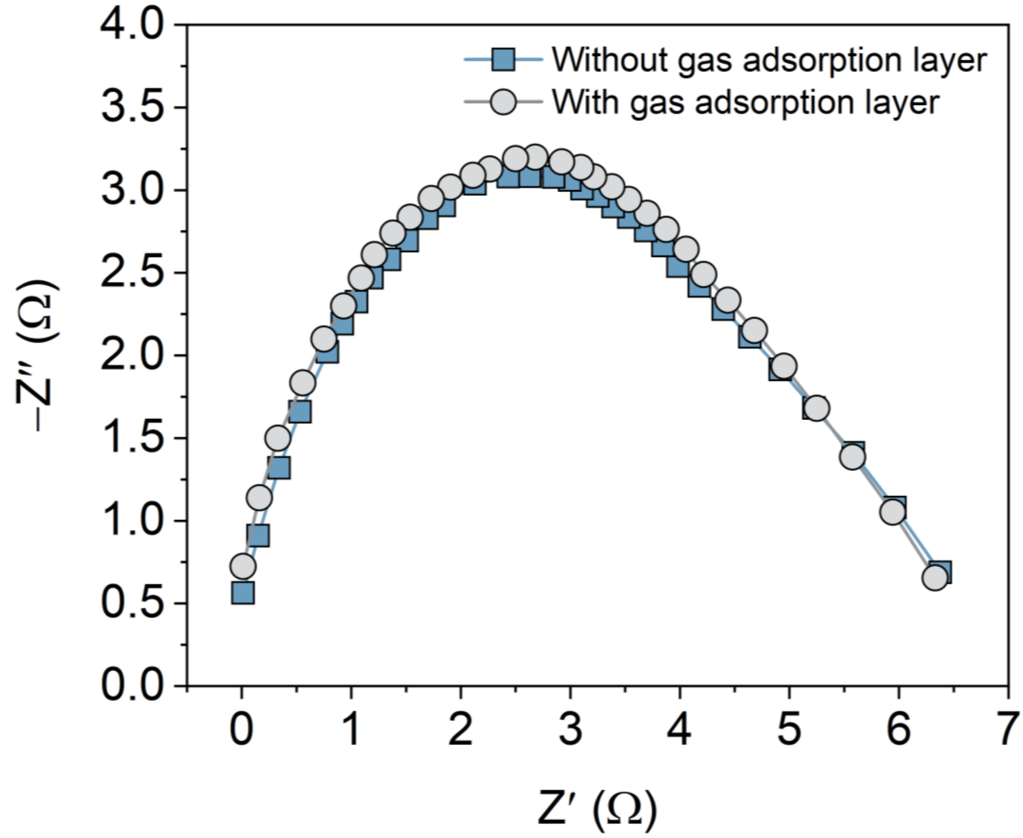


**Fig. S62. EIS of EM-3.** EIS measurements of EM-3 with/without gas adsorption layer (a mixture of middle sized $SiO_2$ microspheres, carbon particles, and CNTs conductive network). The loading density of $SiO_2$ microspheres was 5 mg $cm^{-2}$. The testing electrolyte was 0.25 M $(NH_4)_2SO_4$ and 0.1 M $Na_2SO_4$ solution at the open-circuit voltage.

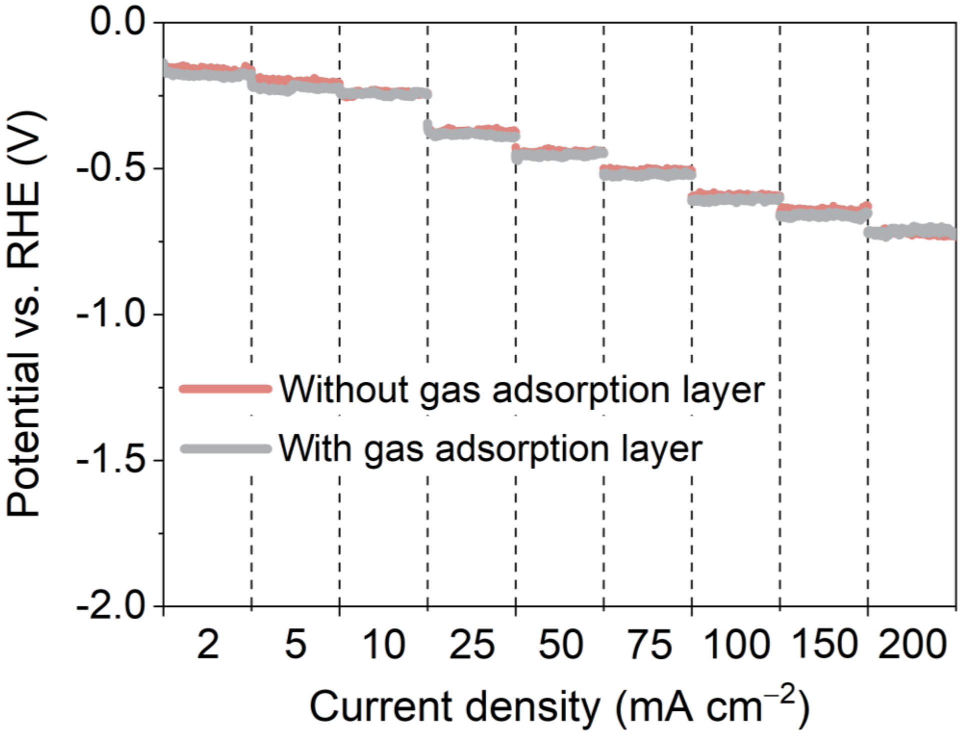


**Fig. S63. Cathodic potential of EM-3.** The recorded cathodic potential of EM-3 with/without gas adsorption layer (a mixture of middle sized $SiO_2$ microspheres, carbon particles, and CNTs conductive network) under different current densities. The loading density of $SiO_2$ microspheres was 5 mg $cm^{-2}$.

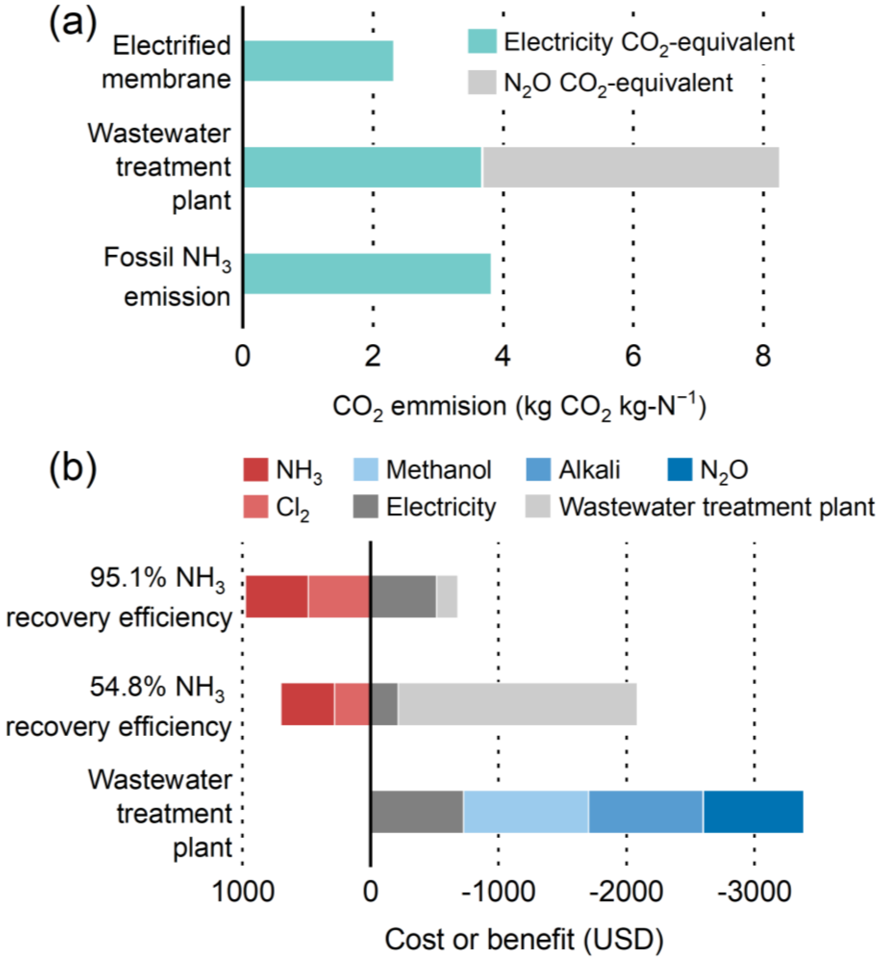


**Fig. S64. Building-scale LCA and TEA analysis of different routes for $NH_3$ removal or synthesis options.** (**a**) LCA towards $CO_2$ emission of different $NH_3$ removal and/or synthesis routes. (**b**) TEA of electrified membrane and wastewater treatment plant options for building-scale $NH_3$ treatment. The electricity-related $CO_2$ emissions and costs were calculated using the U.S. industrial mid-tariff at 0.348 kg-$CO_2$ $kWh^{-1}$ and USD 0.086 $kWh^{-1}$, respectively. The analysis was based on a representative building with 440 residents and the urine generated over one year.

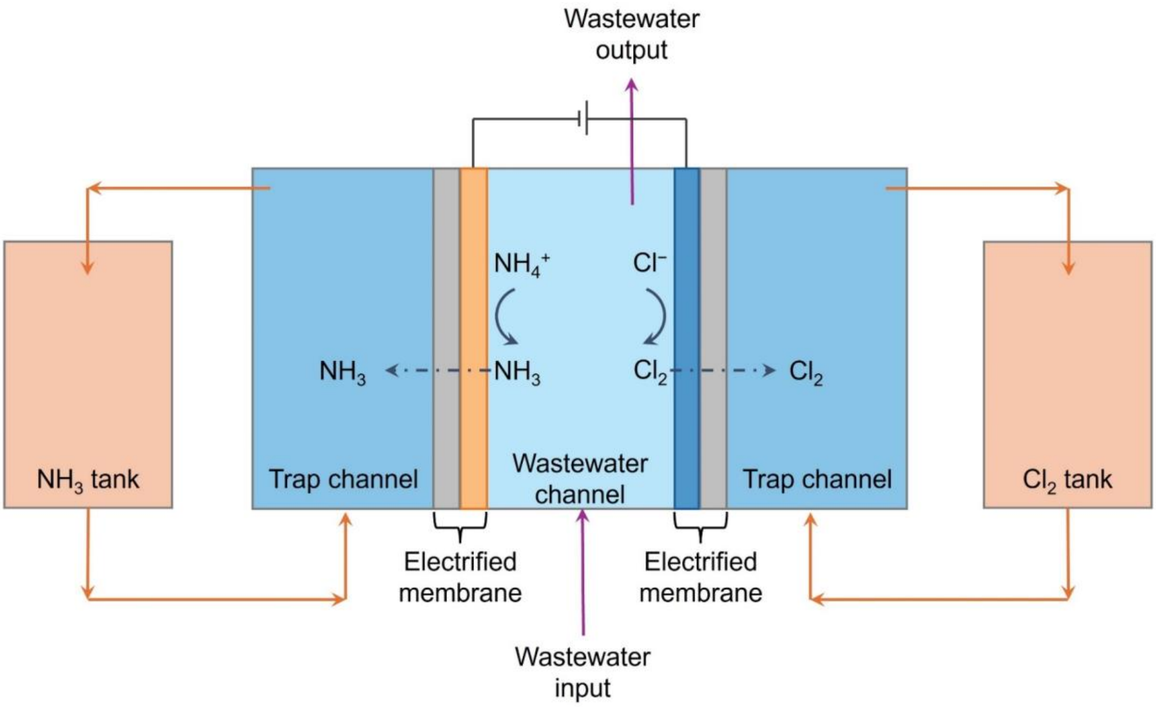


**Fig. S65. Illustration of electrified membrane reactor for $NH_3$ and $Cl_2$ separation from synthetic urine.** The $NH_4^+$ and $Cl^-$ concentrations in the feeding wastewater were 0.25 M and 0.085 M, respectively.

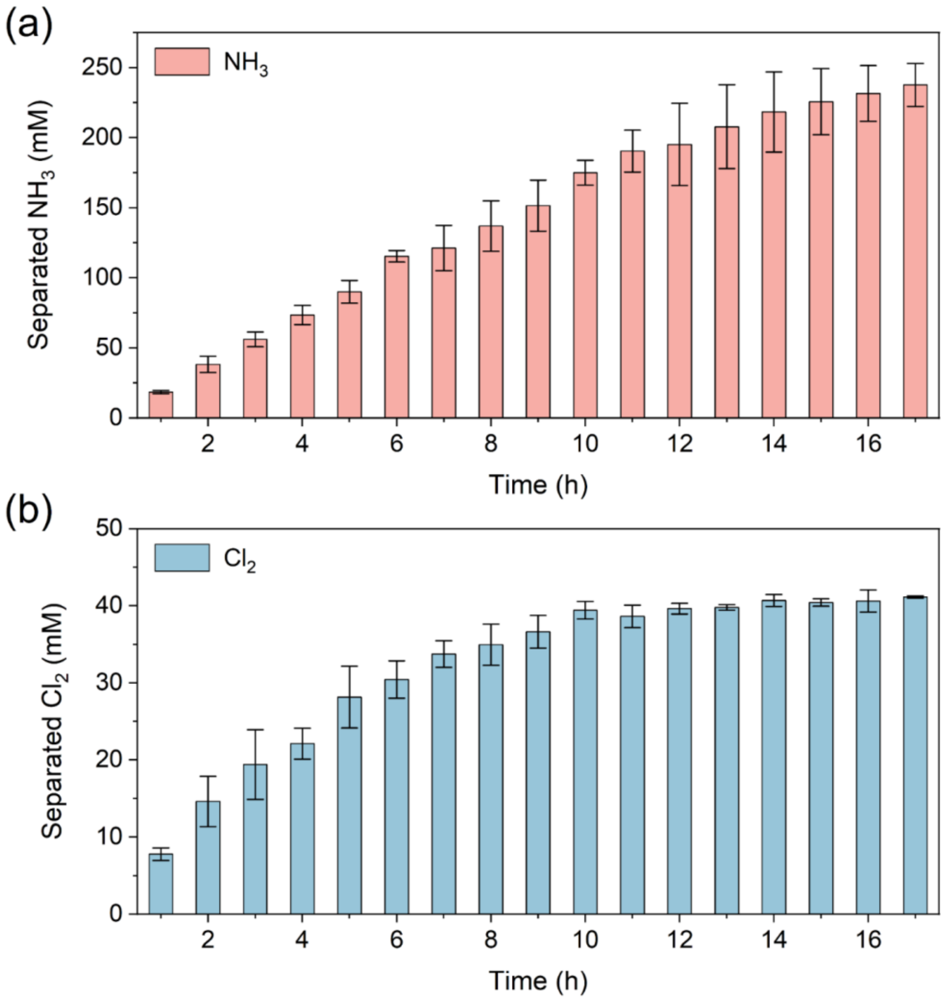


**Fig. S66. $NH_3$ and $Cl_2$ separation from synthetic urine.** The evolution of separated $NH_3$ (**a**) and $Cl_2$ (**b**) over time during the operation of electrified membrane for synthetic urine operation. The experimental data are presented as the mean ± SD from at least three independent experiments.

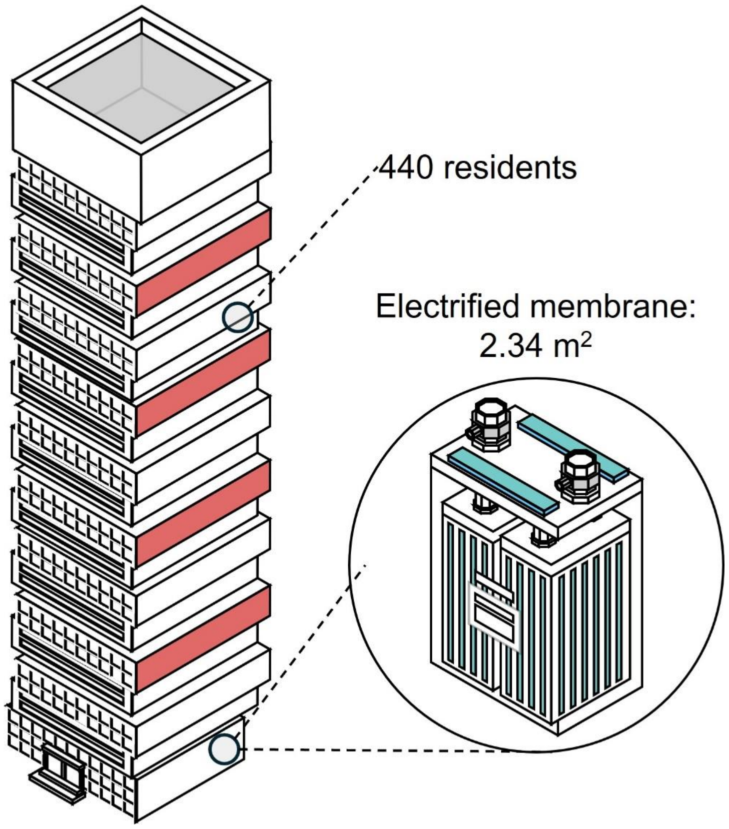


**Fig. S67. Building-scale deployment.** The illustration of deployment of electrified membrane reactor for one building with 440 residents.

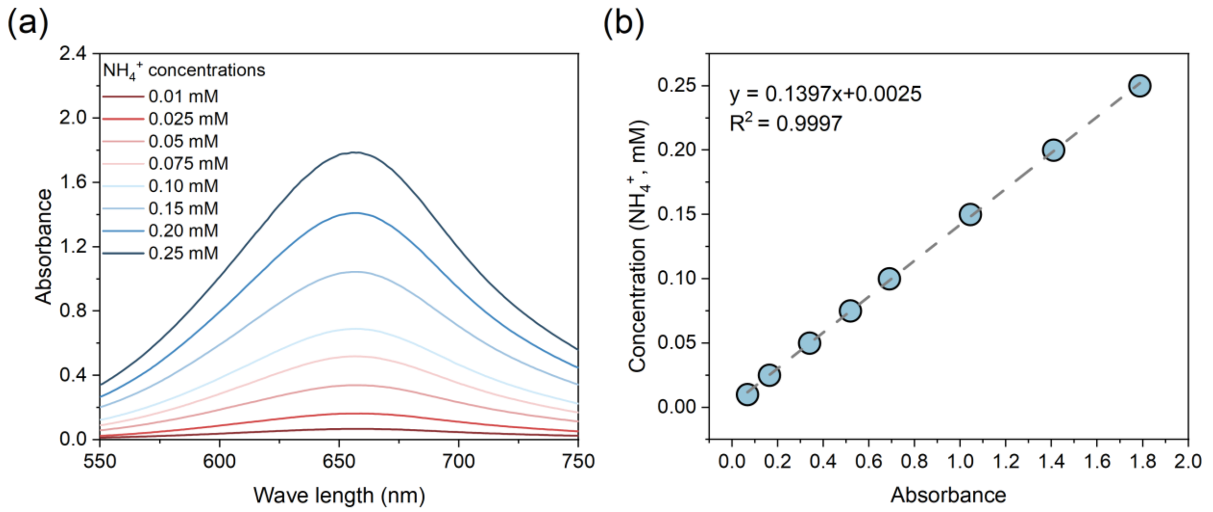


**Fig. S68. Determination of ammonia.** (**a**) The UV-Vis absorption spectra and (**b**) concentration-absorbance calibration curve of $(NH_4)_2SO_4$ solution with a series of standard concentrations using the indophenol blue method.

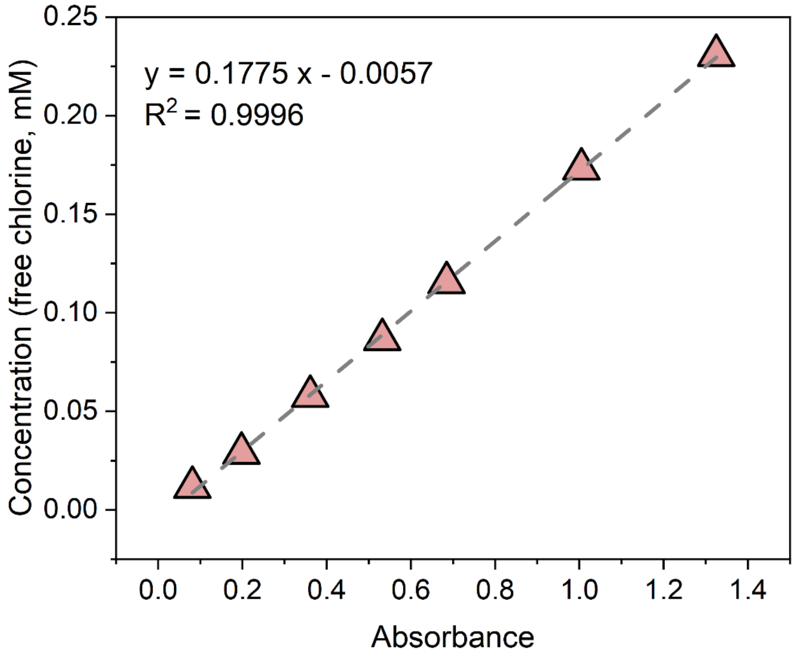


**Fig. S69. Determination of free chlorine.** Concentration-absorbance calibration curve of standard free chlorine solution obtained by the USEPA DPD method.

## Supplementary Tables

### Table S1. Summary of electrochemical-stripping processes for $NH_3$ separation.

| Process or material | Current density (mA cm$^{-2}$) | Energy consumption (kWh kg$^{-1}$-N) | $NH_3$ separation rate (μM cm$^{-2}$ h$^{-1}$) | Current density normalized $NH_3$ separation rate[1] (μM cm$^{-2}$ h$^{-1}$)/(mA cm$^{-2}$) | Ref |
|---|---|---|---|---|---|
| **Electrochemical Stripping** | | | | | |
| Electrochemical cell + Air stripping | 5.0 | 13.0 | 81.8 | 16.4 | *(30)* |
| | 1.0 | 6.7 | 29.5 | 29.5 | *(31)* |
| | 2.0 | 11.1 | 35.4 | 17.7 | |
| | 3.0 | 16.8 | 38.1 | 12.7 | |
| | 1.0 | 13.1 | 14.9 | 14.9 | |
| | 2.0 | 16.7 | 28.3 | 14.1 | |
| | 3.0 | 26.0 | 28.9 | 9.6 | |
| Electrochemical cell + Membrane stripping | 2.0 | 7.3 | 44.9 | 22.5 | *(70)* |
| | 5.0 | 15.6 | 99.7 | 19.9 | |
| | 4.0 | 7.9 | 17.9 | 4.5 | *(35)* |
| | 10 | 8.5 | 21.1 | 2.1 | *(33)* |
| | 5.3 | 28.9 | 10.4 | 2.0 | *(32)* |
| | 5.0 | 17.7 | 42.9 | 8.6 | *(34)* |
| | 5.0 | 11.8 | 53.2 | 10.7 | |
| | 5.0 | 6.6 | 12.5 | 2.5 | |
| | 5.0 | 7.5 | 12.4 | 2.5 | |
| | 7.5 | 35.8 | 57.5 | 7.7 | |
| | 7.5 | 6.5 | 22.2 | 2.9 | |
| | 7.5 | 6.3 | 22.3 | 3.0 | |
| | 10.0 | 13.3 | 26.7 | 2.7 | |
| **Average values** | | **13.9** | **35.0** | **10.3** | |
| **Electrified Membrane** | | | | | |
| Nickel + PVDF membrane | 0.7 | 17.2 | 8.2 | 11.8 | *(36)* |
| | 1.43 | 21.5 | 15.0 | 10.5 | |
| | 2.14 | 37.8 | 15.7 | 7.3 | |
| CNT + PTFE membrane | 0.1 | 2.02 | 11.1 | 111.3 | *(39)* |
| | 0.47 | 7.0 | 9.0 | 19.2 | *(37)* |

| | | | | | |
|---|---|---|---|---|---|
| Stainless steel + PTFE membrane | 1.5 | 9.7 | 61.9 | 41.3 | *(38)* |
| | 6.0 | 16.1 | 206.3 | 34.4 | |
| | 12.0 | 39.2 | 223.5 | 18.6 | |
| Carbon cloth + Hydrophobic polypropylene | 1.56 | 22.1 | 19.3 | 12.4 | *(40)* |
| | 6.25 | 69.2 | 42.1 | 6.7 | |
| **Average values** | | **24.2** | **61.2** | **27.4** | |

Note: [1] was calculated by $NH_3$ separation flux value dividing current density value.

**Table S2. Composition of real urine and real manure wastewater.**

| | **Real urine** (*33*) | **Real manure wastewater** (*71*) |
|---|---|---|
| Total ammonia nitrogen | 0.273 M | 0.497 M |
| Sodium | 0.074 M | 0.148 M |
| Potassium | 0.038 M | 0.183 M |
| Chloride | 0.086 M | 0.047 M |
| Sulfate | 0.017 M | 0.011 M |
| pH | 9.0 | 9.0-9.2 |

**Table S3. Detailed data of optical surface profiler measurements.**

| Labels | Units | EM-1 | EM-2 | EM-3 |
|---|---|---|---|---|
| Data points | | 307200 | 307200 | 307200 |
| Arithmetic average roughness (Ra) | µm | 0.1947 | 0.1957 | 0.1915 |
| Root mean square roughness (Rq) | µm | 0.2517 | 0.2515 | 0.2520 |
| Maximum profile peak height (Rp) | µm | 2.4268 | 2.4662 | 3.0147 |
| Maximum profile valley depth (Rv) | µm | -1.4591 | -1.7485 | -2.992 |
| Total height of the profile (Rt) | µm | 3.8859 | 4.2147 | 6.0067 |

**Table S4. Measured $NH_3$ separation rates and the corresponding percentages of the theoretical values.**

| Current density | EM-1 | | EM-2 | | EM-3 | |
|---|---|---|---|---|---|---|
| (mA cm$^{-2}$) | Separation rate (mM cm$^{-1}$ h$^{-1}$) | Percentage[1] | Separation rate (mM cm$^{-1}$ h$^{-1}$) | Percentage | Separation rate (mM cm$^{-1}$ h$^{-1}$) | Percentage |
| 2 | 0.04±0.01 | 28.6% | 0.08±0.01 | 57.1% | 0.15±0.03 | 107.1%[3] |
| 5 | 0.07±0.01 | 26.9% | 0.12±0.02 | 46.2% | 0.19±0.02 | 73.1% |
| 10 | 0.09±0.01 | 20.0% | 0.19±0.02 | 42.2% | 0.33±0.02 | 73.3% |
| 25 | 0.15±0.02 | 23.4% | 0.28±0.02 | 43.8% | 0.45±0.03 | 70.3% |
| 50 | 0.19±0.01 | 27.5% | 0.35±0.01 | 50.7% | 0.6±0.03 | 87.0% |
| 75 | 0.21±0.02 | 28.8% | 0.37±0.01 | 50.7% | 0.67±0.04 | 91.8% |
| 100 | 0.23±0.03 | 30.3% | 0.38±0.02 | 50.0% | 0.73±0.03 | 96.1% |
| 150 | 0.27±0.02 | 32.9% | 0.40±0.03 | 48.8% | 0.72±0.05 | 87.8% |
| 200 | 0.33±0.02 | 37.1% | 0.41±0.04 | 46.1% | 0.73±0.04 | 82.0% |
| Average[2] | | 28.4±5.0% | | 48.4±4.5% | | 85.4±12.1% |

[1] Percentage (%) was calculated by dividing the measured $NH_3$ separation rate by the corresponding theoretical $NH_3$ separation rate and multiplying the result by 100.

[2] The average value was obtained from the percentage values of EM-1, EM-2, or EM-3 across the current density range of 2-200 mA cm$^{-2}$. The corresponding average value was used as a correction coefficient in the predictive model for $NH_3$ separation value. The resulting corrected model curves were then fitted to the experimentally measured $NH_3$ separation values of EM-1, EM-2, and EM-3, respectively, as shown in **Fig. S37**.

[3] The value of 107.1% was obtained at very low current density and is likely due to minor experimental/measurement errors during sampling and $NH_3$ concentration measurements near the theoretical limit.

Note: The experimental data are presented as the mean ± SD from at least three independent experiments.

**Table S5. Average operating potentials at different current densities during $NH_3$ separation.**

| Current density | Measured potential (V vs. RHE) | | |
|---|---|---|---|
| (mA $cm^{-2}$) | **EM-1** | **EM-2** | **EM-3** |
| 2 | −0.18±0.01 | −0.17±0.01 | −0.16±0.01 |
| 5 | −0.27±0.01 | −0.21±0.00 | −0.20±0.01 |
| 10 | −0.37±0.01 | −0.25±0.00 | −0.24±0.01 |
| 25 | −0.60±0.01 | −0.36±0.00 | −0.37±0.01 |
| 50 | −0.77±0.02 | −0.41±0.01 | −0.44±0.01 |
| 75 | −1.01±0.02 | −0.49±0.00 | −0.51±0.01 |
| 100 | −1.29±0.03 | −0.56±0.00 | −0.59±0.00 |
| 150 | −1.70±0.04 | −0.59±0.00 | −0.64±0.01 |
| 200 | −2.09±0.06 | −0.65±0.00 | −0.72±0.01 |

Note: The experimental data are presented as the mean ± SD from at least three independent experiments.

**Table S6. Illustration and comparison of various routes for $NH_3$ synthesis.**

| Route | Brief description | Typical status | Direct $CO_2$ emissions ($t\text{-}CO_2$ per $t\text{-}NH_3$) | Indicative cost (USD per $t\text{-}NH_3$) | Main advantages | Main limitations or risks | Ref. |
|---|---|---|---|---|---|---|---|
| Natural gas steam methane reforming with Haber-Bosch | I. Hydrogen from steam reforming of natural gas. II. $NH_3$ synthesis. | Widely commercial | ~1.8 | ~250-600 | I. Lowest cost in gas-rich regions II. Very mature and scalable. | I. High carbon dioxide intensity II. Exposure to methane leakage and natural-gas price volatility. | (*72*) |
| Natural gas autothermal reforming with Haber-Bosch | I. Hydrogen from autothermal reforming natural gas. II. $NH_3$ synthesis. | Commercial / expanding | ~1.6 | ~250-600 | I. Process design integrates well with future carbon capture. II. Slightly lower inherent emissions than steam reforming. | I. Still fossil-based. II. Carbon dioxide intensity remains high without carbon capture and storage. | (*73*) |
| Coal gasification with Haber-Bosch | I. Hydrogen from coal gasification. II. $NH_3$ synthesis. | Commercial (concentrated in coal regions) | ~2.5-3.8 | ~300-500 | I. Uses domestic coal. II. Energy security where coal is abundant. | I. Highest carbon dioxide intensity and local air-pollution burden II. Large water use. | (*72*) |
| Natural gas steam methane reforming with carbon capture and storage, plus Haber-Bosch | I. Hydrogen generated by steam methane reforming of natural gas, with carbon dioxide captured from the process syngas and | First-of-a-kind to early commercial | ~0.1 | ~400-700 | I. Deep carbon dioxide reduction at relatively modest added cost II. Leverages existing infrastructure. | I. Residual emissions and methane leakage remain. II. Requires carbon dioxide transport and permanent storage. III. Capture energy penalty. | (*72-74*) |

| | | | | | | | 
|---|---|---|---|---|---|---|
| | the furnace flue gas.<br>II. $NH_3$ synthesis. | | | | | |
| Natural-gas autothermal reforming with carbon capture and storage, plus Haber-Bosch | I. Autothermal reforming with integrated carbon capture.<br>II. $NH_3$ synthesis. | Early commercial | ~0.1 | ~400-700 | II. High capture rates feasible<br>II. Suitable for new low-carbon buildings. | I. Availability of storage and long-term liability.<br>II. Added complexity and cost. | (*72-74*) |
| Coal gasification with carbon capture and storage, plus Haber-Bosch | I. Coal gasification with capture of concentrated and diluted carbon dioxide<br>II. $NH_3$ synthesis. | Pilot to early deployments | ~0.2 | ~500-800 | Large absolute carbon dioxide reductions from a very high baseline. | I. Costly.<br>II. Residual emissions and upstream coal impacts remain.<br>III. Water and air-pollution concerns. | (*72, 73*) |
| Water electrolysis using renewable electricity to hydrogen with Haber-Bosch (often called green ammonia) | I. Hydrogen from water electrolysis powered by renewable electricity.<br>II. $NH_3$ synthesis. | Demonstration of early commercial | ≈0 direct (life-cycle depends on electricity mix) | ~470-1,200 (best sites ~300-700 by 2030) | I. Near-zero direct emissions; modular and compatible with renewable build-out.<br>II. Oxygen co-product. | I. Capital intensive.<br>II. Depends on very low cost.<br>III. High capacity factor electricity and improved Haber-Bosch flexibility. | (*72-74*) |
| Methane pyrolysis to hydrogen and solid carbon with Haber-Bosch | I. Thermal splitting of methane into hydrogen and solid carbon.<br>II. $NH_3$ synthesis. | Pilot / early demonstration | ≈0 direct at the plant | Indicatively ~300-600 (project-specific) | I. Very low direct carbon dioxide.<br>II. Potential value from solid carbon products. | I. Limited track record.<br>II. Market and handling solid carbon.<br>III. Methane supply risks. | (*72, 75*) |

| | | | | | | | |
|---|---|---|---|---|---|---|---|
| Biomass gasification (optionally with carbon capture and storage) to hydrogen with Haber-Bosch | I. Hydrogen from biomass feedstocks.<br>II. $NH_3$ synthesis.<br>III. Carbon capture. | Pilot to early projects | Low to potentially net-negative with capture | ~450-970 (feedstock and scale dependent) | I. Potential for carbon-negative ammonia.<br>II. Diversifies feedstocks; rural value chains. | I. Feedstock logistics and land-use constraints.<br>II. Cost dispersion.<br>III. Limited scale. | (*72, 76*) |
| Electrochemical nitrogen reduction (direct electrochemical synthesis of ammonia) | Direct reduction of nitrogen to ammonia in electrochemical cells (low temperature). | Laboratory research | Not established at scale | / | I. Potential for distributed.<br>II. Low temperature synthesis. | I. Current rates and selectivity are far below industrial relevance.<br>II. Contamination and measurement rigor remain challenges. | (*77*) |
| Plasma-catalytic ammonia synthesis | Nitrogen activation in non-thermal plasma coupled with catalytic surfaces to form ammonia. | Laboratory to early pilots | Not established at scale | / | I. Potential dynamic operation with intermittent renewables.<br>II. Avoids high pressure. | I. High energy intensity to date.<br>II. Reactor durability and scale-up unproven. | (*78*) |
| Photocatalytic ammonia synthesis | Light driven catalytic conversion of nitrogen and water into ammonia. | Laboratory research | Not established at scale | / | I. Conceptually simple process.<br>II. Room-temperature operation. | I. Very low demonstrated rates.<br>II. Product verification issues.<br>III. Far from application. | (*77*) |

Note: emission and cost ranges are indicative and depend on site-specific factors (fuel or electricity price, carbon dioxide transport and storage availability, capacity factor, scale, and financing).

**Table S7. Core inputs for TEA/LCA (take U.S. as example).**

| Group | Parameter | Symbol | Value | Unit |
|---|---|---|---|---|
| **Process** | Specific electricity (reactor) | $SEC_N$ | 0.5888 | kWh kg-N$^{-1}$ |
| **Process** | Areal nitrogen recovery rate | $r_N$ | 0.05255 | kg-N m$^{-2}$ h$^{-1}$ |
| **Power** | US industrial electricity price | $p_{elec}$ | 0.082-0.089 | \$ kWh$^{-1}$ |
| **Market** | $NH_3$ price band | $p_{NH3}$ | 350-650 | \$ t$^{-1}$ |
| **LCA** | US grid $CO_2$ intensity | $CI_{grid}$ | 0.348 | kg-$CO_2$ kWh$^{-1}$ |

Note: Considering that the cathodic $NH_3$ separation process will be combined with anodic $Cl_2$ synthetic/separation process in the subsequent chapters of this manuscript, the $SEC_N$ was calculated based on average half-cell potential (vs. RHE) during the long-term $NH_3$ separation operation.

Public U.S. background inputs were taken from open government and commodity-market sources. The industrial electricity price ($p_{elec}$) was based on the U.S. Energy Information Administration *Electric Power Monthly* national industrial tariff series, for which 2025 monthly values fall within 0.082-0.089 \$ kWh$^{-1}$ (*https://www.eia.gov/electricity/monthly/epm_table_grapher.php?t=table_5_03*).

Ammonia market price ($p_{NH3}$) was set to 350-650 USD t$^{-1}$, based on public ammonia market benchmarks (*https://www.worldbank.org/en/research/commodity-markets*), spanning the lower end of recent US Gulf spot ranges (~355 USD t$^{-1}$ in 2024) and higher recent U.S. market quotes (~650 USD t$^{-1}$ in Dec 2025).

For grid emissions, the U.S. average electricity $CO_2$ intensity was taken from the U.S. EPA eGRID database (*https://www.epa.gov/egrid/summary-data*); using the latest eGRID2023 national total output $CO_2$ rate gives 767.2 lb MWh$^{-1}$, equivalent to 0.348 kg-$CO_2$ kWh$^{-1}$. By contrast, the process-specific electricity demand ($SEC_N$) and areal nitrogen recovery rate ($r_N$) were derived from the experimental performance of this work.

**Table S8. Operating economics per 1 kg-N.**

| $NH_3$ price | Revenue | Electricity cost of electrified membrane ($ kg-N$^{-1}$) | | |
|---|---|---|---|---|
| $ t$^{-1}$ | $ kg-N$^{-1}$ | low | mid | high |
| 300 | 0.3643 | 0.0977 | 0.1013 | 0.1048 |
| 500 | 0.6071 | | | |
| 550 | 0.6679 | | | |
| 786 | 0.9544 | | | |

**Table S9. Levelized cost example (calculated based on 100 kg-N d$^{-1}$).**

| **Metric** | **Value** | **Unit** |
|---|---|---|
| Daily capacity | 100 | kg-N d$^{-1}$ |
| Annual N | 36,500 | kg-N y$^{-1}$ |
| Installed area | 79.29 | m$^{-2}$ |
| Module capital expenditure | 101,593 | $ |
| Capital recovery factor | 0.149 | - |
| Annualized capital expenditure | 15,140 | $ y$^{-1}$ |
| Capital expenditure per kg-N | 0.415 | $ kg-N$^{-1}$ |
| Operating expenditure | 0.0506 | $ kg-N$^{-1}$ |
| Levelized cost of nitrogen | 0.466 | $ kg-N$^{-1}$ |
| Break-even $NH_3$ price | 383.5 | $ t$^{-1}$ |

**Table S10. Use-case scenarios.**

| Use-case | Range | $NH_4^+$ (kg-N $m^{-3}$) | Area ($m^2$) | Electricity (kWh $m^{-3}$) | Net change in greenhouse-gas emissions (kg-$CO_2$ $m^{-3}$) |
|---|---|---|---|---|---|
| **Source-separated urine (hydrolyzed)** | low | 1.8 | 1.43 | 1.06 | -4.75 |
| | high | 5.0 | 3.97 | 2.94 | -13.2 |
| **Wastewater treatment plant side streams (centrate or thermal hydrolysis process centrate)** | low | 0.3 | 0.24 | 0.18 | -0.791 |
| | high | 1.5 | 1.19 | 0.88 | -3.96 |
| **Livestock wastewater (lagoon/slurry)** | low | 0.16 | 0.13 | 0.094 | -0.422 |
| | high | 3.4 | 2.70 | 2.00 | -8.97 |
| **Landfill leachate** | low | 2.0 | 1.59 | 1.18 | -5.28 |
| | high | 4.0 | 3.17 | 2.36 | -10.6 |

Assumptions: (1) U.S. industrial mid-tariff electricity price (\$0.086 kWh$^{-1}$), (2) Net change in greenhouse-gas emissions accounts only for electricity use by the electrified membrane.

Because daily wastewater treatment volumes vary across locations and wastewater types, a uniform basis of **1 m$^3$ wastewater** was used in all calculations to facilitate comparison of ammonia-recovery costs across different wastewater streams.

The net change in greenhouse-gas emissions (kg-$CO_2$ $m^{-3}$) is calculated as: the emissions from $NH_3$ recovery by the electrified membrane minus the emissions that would have occurred if the same amount of $NH_3$ had been produced by conventional fossil-based Haber-Bosch synthesis. Negative values indicate net carbon savings relative to the fossil baseline, whereas positive values indicate net added emissions.

Interpretation: (i) At high ammonium concentration, such as urine, the process is both area- and revenue-dense, with a small footprint per $m^{-3}$ day$^{-1}$, strong product revenue per treated volume, and large net $CO_2$ benefits. (ii) Livestock wastewaters also remain attractive, although the required footprint and electricity demand scale approximately with ammonium concentration.

**Table S11. Electricity and chemicals required for downstream wastewater treatment plant.**

| Item | Conservative scenario range informed by literature | | | Units | Notes / Formula | Ref. |
|---|---|---|---|---|---|---|
| Electricity (aeration, mixing, pumping) | 6 | 10 | 14 | kWh per kg-N | Conventional nitrification/ denitrification energy; plant-to-plant variation. | (79) |
| Methanol for denitrification | 2.5 | 2.9 | 3.2 | kg methanol per kg-N | Stoichiometry ≈ 2.5-3.2 g MeOH per g-N removed. | (*80*) |
| Alkalinity demand (as $CaCO_3$-equiv.) | - | 7.14 | - | kg $CaCO_3$-eq per kg-N | Nitrification consumes ≈7.14 g as $CaCO_3$ per g $NH_4^+$-N. | (*81*) |
| If supplied as sodium bicarbonate ($NaHCO_3$) | - | 6.00 | - | kg $NaHCO_3$ per kg-N | Reagent mass = 1.68 × 7.14 × $f_{alk}$; mid assumes make-up fraction $f_{alk}$ = 0.5 → 1.68×7.14×0.5 ≈ 6.00. | / |
| If supplied as lime ($Ca(OH)_2$) | - | 2.64 | - | kg $Ca(OH)_2$ per kg-N | Reagent mass = 0.74 × 7.14 × $f_{alk}$; with $f_{alk}$ = 0.5 → 0.74×7.14×0.5 ≈ 2.64. | / |
| Dewatering polymer (yield-driven) | 0.001 | 0.0015 | 0.003 | kg polymer per kg-N | Typical for extra solids from denitrification. | / |

Note: Electricity (aeration, mixing, pumping) was modelled using a conservative literature-informed range of 6-14 kWh per kg-N, bracketed against reported values for conventional nitrification–denitrification from about 2.3-6.5 kWh per kg-N, with a high whole-plant benchmark of 14.66 kWh per kg-N.

Methanol for denitrification was based on the stoichiometric requirement of 2.47 kg methanol per kg-N, with practical full-scale values typically in the range of about 2.5-3.2 kg kg-$N^{-1}$; the adopted low/mid/high values were 2.5/2.9/3.2 kg kg-$N^{-1}$.

Alkalinity demand was taken as 7.14 kg as $CaCO_3$-equivalent per kg ammonium nitrogen nitrified, following standard nitrification stoichiometry.

$NaHCO_3$ demand was derived, not independently sourced: $NaHCO_3$ = 7.14 × (84/50) × 0.5, giving 6.00 kg $NaHCO_3$ kg-$N^{-1}$. The base alkalinity requirement of 7.14 kg $CaCO_3$-eq kg-$N^{-1}$ follows standard nitrification stoichiometry.

$Ca(OH)_2$ demand was derived from the same alkalinity requirement using equivalent-weight conversion: $Ca(OH)_2 = 7.14 \times (37/50) \times 0.5$, giving 2.64 kg $Ca(OH)_2$ kg-$N^{-1}$. The underlying alkalinity consumption of 7.14 kg $CaCO_3$-eq kg-$N^{-1}$ is from standard nitrification stoichiometry.

Dewatering polymer was derived from typical sludge-dewatering polymer doses of roughly 5-15 g per kg-dry solids and an assumed incremental solids production of 0.2 kg-dry solids per kg-N removed, yielding 0.001-0.003 kg-polymer per kg-N. This row is therefore a scenario assumption rather than a directly tabulated public constant.

**Table S12. Market price range of chemicals.**

| Commodity | Low | Mid | High | Units | Notes (basis) |
|---|---|---|---|---|---|
| Methanol | 0.40 | 0.65 | 0.90 | $ per kg | Global posted/spot spans: Asia posted ~$400-420/ton; North America posted contract ~$891/ton (non-discounted). |
| Calcium carbonate ($CaCO_3$) | 0.30 | 0.40 | 0.60 | $ per kg | Industrial GCC/PCC; North America ~$0.33/kg (Aug 2025); other sources track up to ~$0.59/kg in early 2025. |
| Sodium bicarbonate ($NaHCO_3$) | 0.35 | 0.56 | 1.25 | $ per kg | Technical/industrial ~$0.35-0.65/kg; U.S. Sep-2025 North America ~$0.56/kg; imported food/pharma grade averages up to $1.25/kg (2024 import price). |
| Lime (calcium hydroxide, $Ca(OH)_2$) | 0.19 | 0.21 | 0.24 | $ per kg | U.S. average hydrated lime at plant ~$240/t; quicklime ~$190/t. |

**Table S13. Nitrous oxide ($N_2O$) emission and its economic damage.**

| Assumption | Low | Mid | High | Units | Notes / Formula | Ref. |
|---|---|---|---|---|---|---|
| $N_2O$-N emission factor (share of influent N → $N_2O$-N) | 0.5% | 1.1% | 1.6% | kg $N_2O$-N per kg-N | Plant-wide factors from literature ranges. | (*82*) |
| $N_2O$ mass emitted | 0.00786 | 0.01729 | 0.02514 | kg $N_2O$ per kg-N | $N_2O$ = ($N_2O$-N) × 44/28. | / |
| Economic damage of $N_2O$ | \$0.42 | \$0.93 | \$1.36 | \$ per kg-N | Using U.S. EPA social cost of $N_2O$ = \$54,000 per t $N_2O$; cost = (kg $N_2O$) × 54,000/1,000. | (*83*) |

Note: The midpoint $N_2O$ emission factor was set to 1.1% of influent N emitted as $N_2O$-N, following the revised default proposed by de Haas et al. (*82*), whereas the high case used the IPCC 2019 default of 1.6% and the low case used a conservative 0.5%, consistent with full-scale plant-wide measurements near 0.58%. $N_2O$ mass was then obtained by multiplying $N_2O$-N by 44/28, and the economic damage was calculated using the U.S. EPA social cost of $N_2O$ of \$54,000 $t^{-1}$ $N_2O$.

**Table S14. $CO_2$-equivalent emissions of different processes (per kg-N).**

| Case | Electricity $CO_2$-equivalent | $N_2O$ $CO_2$-equivalent | Total | Notes (assumptions) |
|---|---|---|---|---|
| | (kg $CO_2$ kg-$N^{-1}$) | | | |
| $NH_3$ from Haber-Bosch | - | - | 3.04-4.61 | 2.5-3.8 t $CO_2$/t $NH_3$; per-N via ×17/14. |
| Wastewater treatment plant nitrogen removal (mid-case) | 3.68 | 4.72 | 8.40 | 10 kWh/kg-N × 0.368 kg-$CO_2$/kWh. $N_2O$ from 1.1% N→$N_2O$-N, $GWP_{100}(N_2O)$ is 273. |
| Wastewater treatment plant nitrogen removal (low-case) | 2.21 | 2.15 | 4.36 | 6 kWh/kg-N; 0.5% N→$N_2O$-N. |
| Wastewater treatment plant nitrogen removal (high-case) | 5.15 | 6.86 | 12.01 | 14 kWh/kg-N; 1.6% N→$N_2O$-N. |
| Source-separation, electrified membrane – 54.8% $NH_3$ recovery efficiency | 1.93 | ~0 | 1.93 | kWh/kg-N = 4.31×(17/14)=5.2336; $CO_2$e = 5.2336×0.368=1.926 No biological $N_2O$. |
| Source-separation, electrified membrane – 95.1% $NH_3$ recovery efficiency | 2.72 | ~0 | 2.72 | kWh/kg-N = 6.08×(17/14)=7.3829; $CO_2$e = 7.3829×0.368=2.717 No biological $N_2O$. |

**Table S15. Key operating parameters of the electrified membrane reactor.**

| Metric | 54.8% $NH_3$ recovery efficiency | 95.1% $NH_3$ recovery efficiency |
|---|---|---|
| $NH_4^+$ concentration | 0.25 mol $L^{-1}$ | |
| $Cl^-$ concentration | 0.085 mol $L^{-1}$ | |
| Synthetic urine volume | 1 L | |
| Electrified membrane area | 50 $cm^2$ for cathode, 50 $cm^2$ for anode | |
| Current value | 0.5 A | |
| Average cell voltage | 2.51±0.09 V | 2.89±0.14 V |
| Separated $NH_3$ | 136.91±17.96 mM | 237.68±15.33 mM |
| Separated $Cl_2$ | 34.95±2.67 mM | 41.13±0.15 mM |
| Energy consumption | 5.24 kWh $kg^{-1}$-N<br>4.31 kWh $kg^{-1}$-$NH_3$ | 7.38 kWh $kg^{-1}$-N<br>6.08 kWh $kg^{-1}$-$NH_3$ |
| Electricity price | \$0.086 $kWh^{-1}$ (U.S. industrial mid-tariff) | |
| Electric cost | 0.45 USD $kg^{-1}$-N<br>0.37 USD $kg^{-1}$-$NH_3$ | 0.63 USD $kg^{-1}$-N<br>0.52 USD $kg^{-1}$-$NH_3$ |

Note: The experimental data are presented as the mean ± SD from at least three independent experiments.

**Table S16. Building archetype and wastewater loading.**

| Metric | Value |
|---|---|
| Floors | 20 |
| Apartments | 200 |
| Residents | 440 |
| Urine capture | 1.5 L $person^{-1}$ $day^{-1}$ → 0.66 $m^3$ $day^{-1}$ captured at the building |
| Total ammoniacal nitrogen (TAN) | 3.5 g-N $L^{-1}$ → 2.31 kg-N $day^{-1}$ |
| Theoretical ammonia equivalent | 2.805 kg-$NH_3$ $day^{-1}$ (using 17/14 mass ratio) |

**Table S17. Treatment cost and potential economic loss associated with $N_2O$ emissions for urine wastewater from a single building (440 residents) treated at a wastewater treatment plant without source separation.**

| **Line item** | **$NaHCO_3$ option** (annual cost) | **$Ca(OH)_2$ option** (annual cost) |
|---|---|---|
| Electricity | $725 | $725 |
| Methanol | $978 | $978 |
| Alkalinity reagent | $1,517 | $270 |
| Dewatering polymer | $3 | $3 |
| $N_2O$ externality (economic loss) | $784 | $784 |
| **TOTAL** | **$4,008** | **$2,760** |

**Table S18. Calculated parameters at different $NH_3$ recovery efficiencies.**

| Metric | 54.8% $NH_3$ recovery efficiency | 95.1% $NH_3$ recovery efficiency |
|---|---|---|
| Specific electricity | 4.31 kWh $kg^{-1}$-$NH_3$ | 6.08 kWh $kg^{-1}$-$NH_3$ |
| Areal rate | 3.4 mol $m^{-2}$ $h^{-1}$ → 0.0578 kg-$NH_3$ $m^{-2}$ $h^{-1}$ | 2.8 mol $m^{-2}$ $h^{-1}$ → 0.0476 kg-$NH_3$ $m^{-2}$ $h^{-1}$ |
| Minimal electrified membrane area to hit target in 24 h | 1.112 $m^2$ | 2.335 $m^2$ |
| Electricity price | $0.086 $kWh^{-1}$ (U.S. industrial mid-tariff) | |
| Pumps and auxiliaries | 0.22 kWh $day^{-1}$ (allowance) | |
| Molar ratios of obtained $Cl_2$:$NH_3$ | 0.255 | 0.173 |

Note: Chlorine is oxidized at the anode and separated in the same skid. Batch measurements provide molar ratios of chlorine to ammonia produced. Chlorine mass is calculated as moles of $NH_3$ × ratio × 70.906 g $mol^{-1}$.

**Table S19. Cost and profit at different $NH_3$ recovery efficiencies.**

| Metric | 54.8% $NH_3$ recovery efficiency | 95.1% $NH_3$ recovery efficiency | Units |
|---|---|---|---|
| **Source separation cost** | | | |
| Energy use (daily: reactor, pumps) | 6.87 | 16.44 | kWh $day^{-1}$ |
| Energy use (annual) | 2507.3 | 6000.1 | kWh $year^{-1}$ |
| Power cost (annual at \$0.086 $kWh^{-1}$) | \$215.6 | \$516.0 | USD $year^{-1}$ |
| **Residual ammonium treatment cost** | | | |
| Wastewater treatment plant | \$1869.1 | \$165.9 | USD $year^{-1}$ |
| **Profits** | | | |
| $NH_3$ (daily) | 1.543 | 2.668 | kg $day^{-1}$ |
| $NH_3$ (annual) | 563.1 | 973.7 | kg $year^{-1}$ |
| Revenue: $NH_3$ (annual, base) | 281.6 | 486.8 | USD $year^{-1}$ |
| $Cl_2$ (daily) | 1.641 | 1.925 | kg $day^{-1}$ |
| $Cl_2$ (annual) | 598.9 | 702.6 | kg $year^{-1}$ |
| Revenue: $Cl_2$ (annual, base) | \$419.2 | \$491.8 | USD $year^{-1}$ |
| Revenue: Total (annual, base) | \$700.8 | \$978.6 | USD $year^{-1}$ |
| **Total** | **-\$1383.9** | **\$296.7** | **USD $year^{-1}$** |

Note:

• Ammonia (anhydrous): base \$500 $t^{-1}$; range \$350-\$650 $t^{-1}$.

• Chlorine (liquid): base \$700 $t^{-1}$; range \$630-\$780 $t^{-1}$.

• Optional conversions (not monetized here): chlorine → sodium hypochlorite; ammonia → ammonium salts; include if used on site or contracted.

**Table S20. Parameters used for theoretical calculation of interfacial pH and $NH_3$ fraction.**

| **Parameters** | **Valus** |
|---|---|
| Faraday constant (F) | 96485 C $mol^{-1}$ |
| Diffusion coefficients of $H^+$ ($D_{H^+}$) | $7.3\times10^{-9}$ $m^2$ $s^{-1}$ |
| Diffusion coefficients of $OH^-$ ($D_{OH^-}$) | $4.9\times10^{-9}$ $m^2$ $s^{-1}$ |
| Diffusion layer thicknesses of $H^+$ ($\delta_{H^+}$) | 24.5 μm |
| Diffusion layer thicknesses of $OH^-$ ($\delta_{OH^-}$) | 21.5 μm |
| $pK_w$ | 14 |
| Henry's constant ($k_H$) of $NH_3$ | $1.70\times10^3$ Pa $m^3$ $M^{-1}$ |

**Table S21. Calculation parameters of Finite-element multiphysics (FEM).**

| Parameters | Valus |
|---|---|
| Current density | 10 mA $cm^{-2}$ |
| Conductivity of electrode | 100 S $m^{-1}$ |
| Conductivity of liquid | 0.3 S $m^{-1}$ |
| Diffusion rates of hydroxide ions, $NH_4^+$, and $NH_3$ | $1\times10^{-7}$ $m^2$ $s^{-1}$ |
| Concentration of $NH_4^+$ | 0.25 M |
| Liquid pH | 9.0 |
| Liquid phase density | 1000 k $m^{-3}$ |
| Gas phase density | 1.25 kg $m^{-3}$ |
| Liquid phase dynamic viscosity | 0.01 Pa s |
| Gas phase dynamic viscosity | 0.00003.5 Pa s |

**Table S22. Input parameters used in the $NH_3$ separation model calculations (corresponding to Text S3).**

| **Parameters** | **Valus** |
|---|---|
| Elementary charge (e) | $-1.602\times10^{-19}$ C |
| Avogadro's constant ($N_A$) | $6.022\times10^{-23}$ $mol^{-1}$ |
| Exposed geometric area of the electrified membrane (A) | 2.25 $cm^2$ |
| Diffusion coefficient of $NH_4^+$ (D) | $1.76\times10^{-9}$ $m^2$ $s^{-1}$ |
| $NH_4^+$ concentration in bulk solution ($C_{bulk}$) | 0.25 mol $L^{-1}$ |
| $NH_4^+$ concentration on the electrified membrane surface ($C_{surface}$) | $1\times10^{-8}$ mol $L^{-1}$ |
| Charge of $NH_4^+$ | +1 |
| Ionic mobility of $NH_4^+$ ($u_{NH_4^+}$) | $7.6\times10^{-8}$ $m^2$ $V^{-1}$ $s^{-1}$ |
| Flow velocity (u) | $4.444\times10^{-4}$ m $s^{-1}$ |
| Wastewater density (ρ) | 1000 kg $m^{-3}$ |
| Viscosity (μ) | 0.0011 Pa $s^{-1}$ |
| Flow channel length (L) | 0.015 m |
| Hydraulic diameter ($d_h$) | 0.0075 m |

**Supplementary Movies.**

**Movie S1**

Bubbles observed on the surface of the pH-regulating material layer under working conditions of EM-1.

**Movie S2**

Bubbles observed on the surface of the pH-regulating material layer under working conditions of EM-3.

## REFERENCES


1. L.Cross, A. Gruère, J. de Sousa, H. Chtioui, E. Achard, "Public summary – Short-term fertilizer outlook 2024–2025" (IFA Strategic Forum, Madrid, 2024); www.fertilizer.org/resource/public-summary-short-term-fertilizer-outlook-2024-2025/.

2. D. R. MacFarlane, P. V. Cherepanov, J. Choi, B. H. R. Suryanto, R. Y. Hodgetts, J. M. Bakker, F. M. Ferrero Vallana, A. N. Simonov, A roadmap to the ammonia economy. *Joule* **4**, 1186–1205 (2020).

3. W. Verstraete, P. Van de Caveye, V. Diamantis, Maximum use of resources present in domestic "used water". *Bioresour. Technol.* **100**, 5537–5545 (2009).

4. M. Garrido-Baserba, D. L. Sedlak, M. Molinos-Senante, I. Barnosell, O. Schraa, D. Rosso, M. Verdaguer, M. Poch, Using water and wastewater decentralization to enhance the resilience and sustainability of cities. *Nat. Water* **2**, 953–974 (2024).

5. H. Tian, R. Xu, J. G. Canadell, R. L. Thompson, W. Winiwarter, P. Suntharalingam, E. A. Davidson, P. Ciais, R. B. Jackson, G. Janssens-Maenhout, M. J. Prather, P. Regnier, N. Pan, S. Pan, G. P. Peters, H. Shi, F. N. Tubiello, S. Zaehle, F. Zhou, A. Arneth, G. Battaglia, S. Berthet, L. Bopp, A. F. Bouwman, E. T. Buitenhuis, J. Chang, M. P. Chipperfield, S. R. S. Dangal, E. Dlugokencky, J. W. Elkins, B. D. Eyre, B. Fu, B. Hall, A. Ito, F. Joos, P. B. Krummel, A. Landolfi, G. G. Laruelle, R. Lauerwald, W. Li, S. Lienert, T. Maavara, M. MacLeod, D. B. Millet, S. Olin, P. K. Patra, R. G. Prinn, P. A. Raymond, D. J. Ruiz, G. R. van der Werf, N. Vuichard, J. Wang, R. F. Weiss, K. C. Wells, C. Wilson, J. Yang, Y. Yao, A comprehensive quantification of global nitrous oxide sources and sinks. *Nature* **586**, 248–256 (2020).

6. L. Höglund-Isaksson, A. Gómez-Sanabria, Z. Klimont, P. Rafaj, W. Schöpp, Technical potentials and costs for reducing global anthropogenic methane emissions in the 2050 timeframe –Results from the GAINS model. *Environ. Res. Commun.* **2**, 025004 (2020).

7. M. Maurer, P. Schwegler, T. A. Larsen, Nutrients in urine: Energetic aspects of removal and recovery. *Water Sci. Technol.* **48**, 37–46 (2003).

8. C. Smith, A. K. Hill, L. Torrente-Murciano, Current and future role of Haber–Bosch ammonia in a carbon-free energy landscape. *Energ. Environ. Sci.* **13**, 331–344 (2020).

9. K. Kitamori, T. Manders, R. Dellink, A. Tabeau, *OECD Environmental Outlook to 2050: The Consequences of Inaction* (OECD, 2012).

10. A. Aliahmad, P. Simha, B. Vinnerås, J. McConville, Comparative environmental assessment of three urine recycling scenarios: Influence of treatment configurations and life cycle modeling approaches. *Environ. Sci. Technol.* **59**, 21160–21173 (2025).

11. J. T. Trimmer, J. S. Guest, Recirculation of human-derived nutrients from cities to agriculture across six continents. *Nat. Sustain.* **1**, 427–435 (2018).

12. S. P. Hilton, G. A. Keoleian, G. T. Daigger, B. Zhou, N. G. Love, Life cycle assessment of urine diversion and conversion to fertilizer products at the city scale. *Environ. Sci. Technol.* **55**, 593–603 (2021).

13. K. A. Landry, T. H. Boyer, Life cycle assessment and costing of urine source separation: Focus on nonsteroidal anti-inflammatory drug removal. *Water Res.* **105**, 487–495 (2016).

14. S. K. L. Ishii, T. H. Boyer, Life cycle comparison of centralized wastewater treatment and urine source separation with struvite precipitation: Focus on urine nutrient management. *Water Res.* **79**, 88–103 (2015).

15. E. Igos, M. Besson, T. Navarrete Gutiérrez, A. B. Bisinella de Faria, E. Benetto, L. Barna, A. Ahmadi, M. Spérandio, Assessment of environmental impacts and operational costs of the implementation of an innovative source-separated urine treatment. *Water Res.* **126**, 50–59 (2017).

16. A. Aliahmad, P. de Morais Lima, H. Kjerstadius, P. Simha, B. Vinnerås, J. McConville, Consequential life cycle assessment of urban source-separating sanitation systems complementing centralized wastewater treatment in Lund, Sweden. *Water Res.* **268**, 122741 (2025).

17. A. M. Michalak, J. Xia, D. Brdjanovic, A.-N. Mbiyozo, D. Sedlak, T. Pradeep, U. Lall, N. Rao, J. Gupta, The frontiers of water and sanitation. *Nat. Water* **1**, 10–18 (2023).

18. T. A. Larsen, S. Hoffmann, C. Lüthi, B. Truffer, M. Maurer, Emerging solutions to the water challenges of an urbanizing world. *Science* **352**, 928–933 (2016).

19. National Academies of Sciences, Engineering, and Medicine, Division on Earth and Life Studies, Water Science and Technology Board, Committee on the Beneficial Use of Graywater and Stormwater: An Assessment of Risks, Costs, and Benefits, "Using graywater and stormwater to enhance local water supplies: An assessment of risks, costs, and benefits" (National Academies Press, 2016).

20. M. Garrido-Baserba, I. Barnosell, M. Molinos-Senante, D. L. Sedlak, K. Rabaey, O. Schraa, M. Verdaguer, D. Rosso, M. Poch, The third route: A techno-economic evaluation of extreme water and wastewater decentralization. *Water Res.* **218**, 118408 (2022).

21. O. Kavvada, W. A. Tarpeh, A. Horvath, K. L. Nelson, Life-cycle cost and environmental assessment of decentralized nitrogen recovery using ion exchange from source-separated urine through spatial modeling. *Environ. Sci. Technol.* **51**, 12061–12071 (2017).

22. W. A. Tarpeh, K. M. Udert, K. L. Nelson, Comparing ion exchange adsorbents for nitrogen recovery from source-separated urine. *Environ. Sci. Technol.* **51**, 2373–2381 (2017).

23. H. Gong, Z. Wang, X. Zhang, Z. Jin, C. Wang, L. Zhang, K. Wang, Organics and nitrogen recovery from sewage via membrane-based pre-concentration combined with ion exchange process. *Chem. Eng. J.* **311**, 13–19 (2017).

24. H. Ray, F. Perreault, T. H. Boyer, Ammonia recovery from hydrolyzed human urine by forward osmosis with acidified draw solution. *Environ. Sci. Technol.* **54**, 11556–11565 (2020).

25. M. M. Zico, B. C. Ricci, B. G. Reis, N. C. Magalhães, M. C. S. Amaral, Sustainable ammonia resource recovery from landfill leachate by solar-driven modified direct contact membrane distillation. *Sep. Purif. Technol.* **264**, 118356 (2021).

26. S. P. Munasinghe-Arachchige, N. Nirmalakhandan, Nitrogen-fertilizer recovery from the centrate of anaerobically digested sludge. *Environ. Sci. Technol. Lett.* **7**, 450–459 (2020).

27. J. Gao, N. Shi, X. Guo, Y. Li, X. Bi, Y. Qi, J. Guan, B. Jiang, Electrochemically selective ammonia extraction from nitrate by coupling electron- and phase-transfer reactions at a three-phase interface. *Environ. Sci. Technol.* **55**, 10684–10694 (2021).

28. J. Gao, N. Shi, Y. Li, B. Jiang, T. Marhaba, W. Zhang, Electrocatalytic upcycling of nitrate wastewater into an ammonia fertilizer via an electrified membrane. *Environ. Sci. Technol.* **56**, 11602–11613 (2022).

29. O. Z. Coombs, T. Joo, A. B. Botelho Junior, D. Chalise, W. A. Tarpeh, Prototyping and modelling a photovoltaic–thermal electrochemical stripping system for distributed urine nitrogen recovery. *Nat. Water* **3**, 913–926 (2025).

30. A. K. Luther, J. Desloover, D. E. Fennell, K. Rabaey, Electrochemically driven extraction and recovery of ammonia from human urine. *Water Res.* **87**, 367–377 (2015).

31. J. Desloover, A. Abate Woldeyohannis, W. Verstraete, N. Boon, K. Rabaey, Electrochemical resource recovery from digestate to prevent ammonia toxicity during anaerobic digestion. *Environ. Sci. Technol.* **46**, 12209–12216 (2012).

32. J. He, J. Zhou, K. Yang, L. Luo, P. Wang, Z. Wang, J. Ma, Pulsed electric field drives chemical-free membrane stripping for high ammonia recovery from urine. *Water Res.* **251**, 121129 (2024).

33. W. A. Tarpeh, J. M. Barazesh, T. Y. Cath, K. L. Nelson, Electrochemical stripping to recover nitrogen from source-separated urine. *Environ. Sci. Technol.* **52**, 1453–1460 (2018).

34. F. Ferrari, M. Pijuan, S. Molenaar, N. Duinslaeger, T. Sleutels, P. Kuntke, J. Radjenovic, Ammonia recovery from anaerobic digester centrate using onsite pilot scale bipolar membrane electrodialysis coupled to membrane stripping. *Water Res.* **218**, 118504 (2022).

35. L. Xu, S. Liu, S. Zhao, K. Li, A. Cao, J. Wang, A novel electrocoagulation-membrane stripping hybrid system for simultaneous ammonia recovery and contaminant removal. *Sep. Purif. Technol.* **296**, 121377 (2022).

36. K.-Y. Kim, D. A. Moreno-Jimenez, H. Efstathiadis, Electrochemical ammonia recovery from anaerobic centrate using a nickel-functionalized activated carbon membrane electrode. *Environ. Sci. Technol.* **55**, 7674–7680 (2021).

37. A. Iddya, D. Hou, C. M. Khor, Z. Ren, J. Tester, R. Posmanik, A. Gross, D. Jassby, Efficient ammonia recovery from wastewater using electrically conducting gas stripping membranes. *Environ. Sci. Nano* **7**, 1759–1771 (2020).

38. Z. Wang, J. Zhang, Z. Zhang, Q. Zhang, B. Deng, N. Zhang, Z. Cao, G. Wei, S. Xia, Gas permeable membrane electrode assembly with in situ utilization of authigenic acid and base for transmembrane electro-chemisorption to enhance ammonia recovery from wastewater. *Water Res.* **258**, 121655 (2024).

39. S. Qi, W. Zhang, G. Z. Ramon, A. Ronen, Sustainable ammonia recovery in electrochemical membranes: The critical role of electromigration. *J. Membr. Sci.* **726**, 124018 (2025).

40. X. Wang, S. Im, B. Jung, J. Wu, A. Iddya, Q.-R. A. Javier, M. Xiao, S. Ma, S. Lu, B. Jaewon, J. Zhang, Z. J. Ren, C. T. Maravelias, E. M. V. Hoek, D. Jassby, Simple and low-cost electroactive membranes for ammonia recovery. *Environ. Sci. Technol.* **57**, 9405–9415 (2023).

41. A. Angulo, P. van der Linde, H. Gardeniers, M. Modestino, D. Fernández Rivas, Influence of bubbles on the energy conversion efficiency of electrochemical reactors. *Joule* **4**, 555–579 (2020).

42. J. Su, C. B. Musgrave, Y. Song, L. Huang, Y. Liu, G. Li, Y. Xin, P. Xiong, M. M.-J. Li, H. Wu, M. Zhu, H. M. Chen, J. Zhang, H. Shen, B. Z. Tang, M. Robert, W. A. Goddard, R. Ye, Strain enhances the activity of molecular electrocatalysts via carbon nanotube supports. *Nat. Catal.* **6**, 818–828 (2023).

43. Y. Wu, Z. Jiang, X. Lu, Y. Liang, H. Wang, Domino electroreduction of $CO_2$ to methanol on a molecular catalyst. *Nature* **575**, 639–642 (2019).

44. B. Zhou, H. Liu, G. Su, H. Shin, X.-Y. Li, H. Ze, Y. Liang, B. Peng, W. Ni, Y. Chen, W. Zhu, C. Yu, Y. Chen, P. Ou, K. Xie, E. H. Sargent, Electrosynthesis of CO from an electrically pH-shifted DAC post-capture liquid using a catalyst:support amide linkage. *Joule* **9**, 101883 (2025).

45. G. Lee, D. Kim, J.-I. Han, Gas-diffusion-electrode based direct electro-stripping system for gaseous ammonia recovery from livestock wastewater. *Water Res.* **196**, 117012 (2021).

46. C. Choi, X. Wang, S. Kwon, J. L. Hart, C. L. Rooney, N. J. Harmon, Q. P. Sam, J. J. Cha, W. A. Goddard, M. Elimelech, H. Wang, Efficient electrocatalytic valorization of chlorinated organic water pollutant to ethylene. *Nat. Nanotechnol.* **18**, 160–167 (2023).

47. X. Zhang, Z. Wu, X. Zhang, L. Li, Y. Li, H. Xu, X. Li, X. Yu, Z. Zhang, Y. Liang, H. Wang, Highly selective and active $CO_2$ reduction electrocatalysts based on cobalt phthalocyanine/carbon nanotube hybrid structures. *Nat. Commun.* **8**, 14675 (2017).

48. Y. Wu, Z. Jiang, Z. Lin, Y. Liang, H. Wang, Direct electrosynthesis of methylamine from carbon dioxide and nitrate. *Nat. Sustain.* **4**, 725–730 (2021).

49. W. Xu, J. Xiao, Y. Chen, Y. Chen, X. Ling, J. Zhang, Graphene-veiled gold substrate for surface-enhanced raman spectroscopy. *Adv. Mater.* **25**, 928–933 (2013).

50. J. Guo, Y. Zheng, Z. Hu, C. Zheng, J. Mao, K. Du, M. Jaroniec, S.-Z. Qiao, T. Ling, Direct seawater electrolysis by adjusting the local reaction environment of a catalyst. *Nat. Energy* **8**, 264–272 (2023).

51. R. Wang, Y. Yang, J. Guo, Q. Zhang, F. Cao, Y. Wang, L. Han, T. Ling, Cathode catalyst layers modified with Brønsted acid oxides to improve proton exchange membrane electrolysers for impure water splitting. *Nat. Energy* **10**, 880–889 (2025).

52. Y. Yokoyama, K. Miyazaki, Y. Kondo, Y. Miyahara, T. Fukutsuka, T. Abe, In situ local pH measurements with hydrated iridium oxide ring electrodes in neutral pH aqueous solutions. *Chem. Lett.* **49**, 195–198 (2020).

53. S. Zhu, X. Qin, Y. Yao, M. Shao, pH-dependent hydrogen and water binding energies on platinum surfaces as directly probed through surface-enhanced infrared absorption spectroscopy. *J. Am. Chem. Soc.* **142**, 8748–8754 (2020).

54. W. Deng, L. Zhang, L. Li, S. Chen, C. Hu, Z.-J. Zhao, T. Wang, J. Gong, Crucial role of surface hydroxyls on the activity and stability in electrochemical $CO_2$ reduction. *J. Am. Chem. Soc.* **141**, 2911–2915 (2019).

55. X. Yang, J. Nash, N. Oliveira, Y. Yan, B. Xu, Understanding the pH dependence of underpotential deposited hydrogen on platinum. *Angew. Chem. Int. Ed. Engl.* **58**, 17718–17723 (2019).

56. US Environmental Protection Agency (EPA), "Understanding global warming potentials" (EPA, 2025); www.epa.gov/ghgemissions/understanding-global-warming-potentials.

57. J. He, C. Zhang, Y. Yang, J. Kang, C. Zhang, D. He, J. Ma, Chlorine-mediated ammonia and organics transformation during electrochemical ammonia recovery from human urine. *Environ. Sci. Technol.* **59**, 13096–13107 (2025).

58. K. P. Kuhl, E. R. Cave, D. N. Abram, T. F. Jaramillo, New insights into the electrochemical reduction of carbon dioxide on metallic copper surfaces. *Energ. Environ. Sci.* **5**, 7050–7059 (2012).

59. G. Zhu, S. Zhao, Y. Yu, X. Fan, K. Liu, X. Quan, Y. Liu, Tuning local proton concentration and *OOH intermediate generation for efficient acidic $H_2O_2$ electrosynthesis at ampere-level current density. *Angew. Chem. Int. Ed. Engl.* **64**, e202503626 (2025).

60. K. Nagita, S. Nakanishi, Y. Mukouyama, Finite element analysis of local pH variations in electrolysis with porous electrodes, considering water self-ionization. *ACS Appl. Mater. Interfaces* **16**, 59443–59451 (2024).

61. S. Anantharaj, S. R. Ede, K. Karthick, S. Sam Sankar, K. Sangeetha, P. E. Karthik, S. Kundu, Precision and correctness in the evaluation of electrocatalytic water splitting: Revisiting activity parameters with a critical assessment. *Energ. Environ. Sci.* **11**, 744–771 (2018).

62. F. Yin, H. Liu, The j–pH diagram of interfacial reactions involving $H^+$ and $OH^-$. *J. Energy Chem.* **50**, 339–343 (2020).

63. J. Gao, Q. Ma, Y. Zhang, S. Xue, G. Guo, B. Pan, H. Q. Yu, W. Zhang, Direct electrosynthesis and separation platform for chlorine from saline water. *Environ. Sci. Technol.* **59**, 9837–9848 (2025).

64. Y. Liang, Y. Li, H. Wang, J. Zhou, J. Wang, T. Regier, H. Dai, $Co_3O_4$ nanocrystals on graphene as a synergistic catalyst for oxygen reduction reaction. *Nat. Mater.* **10**, 780–786 (2011).

65. S. Niu, S. Li, Y. Du, X. Han, P. Xu, How to reliably report the overpotential of an electrocatalyst. *ACS Energy Lett.* **5**, 1083–1087 (2020).

66. H. Zhang, D. Raciti, A. S. Hall, Disordered interfacial $H_2O$ promotes electrochemical C–C coupling. *Nat. Chem.* **17**, 1161–1168 (2025).

67. W. Stöber, A. Fink, E. Bohn, Controlled growth of monodisperse silica spheres in the micron size range. *J. Colloid Interface Sci.* **26**, 62–69 (1968).

68. G. H. Bogush, M. A. Tracy, C. F. Zukoski, Preparation of monodisperse silica particles: Control of size and mass fraction. *J. Non Cryst. Solids* **104**, 95–106 (1988).

69. A. Van Blaaderen, J. Van Geest, A. Vrij, Monodisperse colloidal silica spheres from tetraalkoxysilanes: Particle formation and growth mechanism. *J. Colloid Interface Sci.* **154**, 481–501 (1992).

70. L. Zhao, Z. Du, X. Tai, Y. Ma, One-step facile fabrication of hydrophobic $SiO_2$ coated super-hydrophobic/super-oleophilic mesh via an improved Stöber method to efficient oil/water separation. *Colloids Surf. A* **623**, 126404 (2021).

71. R. Wang, K. Yang, C. Wong, H. Aguirre-Villegas, R. Larson, F. Brushett, M. Qin, S. Jin, Electrochemical ammonia recovery and co-production of chemicals from manure wastewater. *Nat. Sustain.* **7**, 179–190 (2024).

72. The International Energy Agency (IEA), "Ammonia technology roadmap" (IEA, 2021); www.iea.org/reports/ammonia-technology-roadmap.

73. International Renewable Energy Agency (IRENA), "Innovation outlook: Renewable ammonia" (978-92-9260-423-3, IRENA and AEA, 2022); www.irena.org/publications/2022/May/Innovation-Outlook-Renewable-Ammonia.

74. M. Mersch, N. Sunny, R. Dejan, A. Y. Ku, G. Wilson, S. O'Reilly, G. Soloveichik, J. Wyatt, N. Mac Dowell, A comparative techno-economic assessment of blue, green, and hybrid ammonia production in the United States. *Sustain. Energy Fuels* **8**, 1495–1508 (2024).

75. J. Holler, D. Vine, "Methane pyrolysis for hydrogen production" (Center for Climate and Energy Solutions, 2025); www.c2es.org/document/methane-pyrolysis-for-hydrogen-production.

76. J. Lundgren, "Biomass gasification for hydrogen production" (International Energy Agency Bioenergy, 2025); www.ieabioenergy.com/blog/publications/biomass-gasification-for-hydrogen-production.

77. L. Collado, A. H. Pizarro, M. Barawi, M. García-Tecedor, M. Liras, Light-driven nitrogen fixation routes for green ammonia production. *Chem. Soc. Rev.* **53**, 11334–11389 (2024).

78. Y. Zhang, J. Niu, S. Chen, Y. Chen, H. Chen, X. Fan, Ammonia synthesis by nonthermal plasma catalysis: A review on recent research progress. *J. Phys. D Appl. Phys.* **57**, 323001 (2024).

79. B. Wett, Development and implementation of a robust deammonification process. *Water Sci. Technol.* **56**, 81–88 (2007).

80. T. L. Theis, A. Hicks, “Methanol use in wastewater denitrification” (Exponent, University of Illinois at Chicago, 2012); www.methanol.org/wp-content/uploads/2016/06/Exponent-Methanol-Denitrification-Report-July-2012-1.pdf.

81. US Environmental Protection Agency (EPA), “Nutrient control design manual” (EPA/600/R-09/012, 2009); www.epa.gov/sites/default/files/2019-02/documents/nutrient-control-design-manual-state-tech.pdf.

82. D. de Haas, J. Andrews, Nitrous oxide emissions from wastewater treatment-Revisiting the IPCC 2019 refinement guidelines. *Environ. Chall.* **8**, 100557 (2022).

83. US Environmental Protection Agency (EPA), “Report on the social cost of greenhouse gases” (EPA-HQ-OAR-2021-0317, 2023); www.epa.gov/system/files/documents/2023-12/epa_scghg_2023_report_final.pdf.
